# Beyond the Mean: A Flexible Framework for Studying Causal Effects Using Linear Models

**DOI:** 10.1007/s11336-021-09811-z

**Published:** 2021-12-11

**Authors:** Christian Gische, Manuel C. Voelkle

**Affiliations:** Department of Psychology, Humboldt University Berlin, Rudower Chaussee 18, 12489 Berlin, Germany

**Keywords:** causal inference, structural equation modeling, graph-based causal models, acyclic directed mixed graphs

## Abstract

Graph-based causal models are a flexible tool for causal inference from observational data. In this paper, we develop a comprehensive framework to define, identify, and estimate a broad class of causal quantities in linearly parametrized graph-based models. The proposed method extends the literature, which mainly focuses on causal effects on the mean level and the variance of an outcome variable. For example, we show how to compute the probability that an outcome variable realizes within a target range of values given an intervention, a causal quantity we refer to as the probability of treatment success. We link graph-based causal quantities defined via the *do*-operator to parameters of the model implied distribution of the observed variables using so-called causal effect functions. Based on these causal effect functions, we propose estimators for causal quantities and show that these estimators are consistent and converge at a rate of $N^{-1/2}$ under standard assumptions. Thus, causal quantities can be estimated based on sample sizes that are typically available in the social and behavioral sciences. In case of maximum likelihood estimation, the estimators are asymptotically efficient. We illustrate the proposed method with an example based on empirical data, placing special emphasis on the difference between the interventional and conditional distribution.

**Supplementary Information:**

The online version contains supplementary material available at 10.1007/s11336-021-09811-z.

## Graph-Based Models for Causal Inference

The graph-based approach to causal inference was primarily formalized by Judea Pearl (1988, 1995, 2009) and Spirtes, Glymour, and Scheines (2001). A causal graph represents a researcher’s theory about the causal structure of the data-generating mechanism. Based on a causal graph, causal inference can be conducted using the interventional distribution, from which standard causal quantities such as average treatment effects (ATEs) can be derived. In the most general formulation, a causal graph is accompanied by a set of nonparametric structural equations. Thus, a common acronym for Pearl’s general nonparametric model is NPSEM, which stands for *n*on-*p*arametric *s*tructural *e*quation *m*odel (Pearl, 2009; Shpitser, Richardson, & Robins, 2020).

Graph-based causal models share many common characteristics with the traditional literature on structural equation models (SEM) prevalent in the social and behavioral sciences and economics (Bollen & Pearl, 2013; Heckman & Pinto, 2015; Pearl, 2009, 2012). However, these two approaches also differ in several aspects including the underlying assumptions (e.g., graph-based models assume modularity), notational conventions (e.g., the meaning of bidirected edges in graphical representations), research focus (e.g., nonparametric identification in graph-based models vs. parametric estimation in traditional SEM), and standard procedures.

Graph-based procedures often focus on a single causal quantity of interest (e.g., ATE) and establishing its causal identification based on a minimal set of assumptions (e.g., without making parametric assumptions). Causal quantities are well defined via the *do*-operator and the resulting interventional distribution and causal identification can be established based on graphical tools such as the back-door criterion (Pearl, 1995) or a set of algebraic rules called *do*-calculus (Shpitser & Pearl, 2006; Tian & Pearl, 2002a). The central insights developed within the graph-based approach relate to causal identification, whereas less attention has been devoted to the estimation of causal quantities.^1^

On the other hand, the traditional literature on SEM frequently assumes parametrized (often linear) models and usually focuses on identification and estimation of the *entire* model.^2^ Causal quantities such as direct, indirect and total effects can be defined based on reduced-form equations and partial derivatives (Alwin & Hauser, 1975; Bollen, 1987; Stolzenberg, 1980). A main focus within the traditional SEM literature lies on the model implied joint distribution of observed variables and its statistical modeling. A considerable body of literature is available on model identification (Bekker, Merckens, & Wansbeek, 1994; Bollen, 1989; Fisher, 1966; Wiley, 1973) and estimation (Browne, 1984; Jöreskog, 1967; Satorra & Bentler, 1994) for parametrized SEM.

In this paper, we combine causal quantities from graph-based models with identification and estimation results from the traditional literature on *linear* SEM. For this purpose, we formalize the *do*-operator using matrix algebra in the section on “Graph-Based Causal Models with Linear Equations.” Based on this matrix representation, we derive a closed-form parametric expression of the interventional distribution and several causal quantities in the section entitled “Interventional Distribution.” Linear graph-based models imply a parametrized joint distribution of the observed variables. We define causal effect functions as a mapping from the parameters of the joint distribution of observed variables onto the causal quantities defined via the *do*-operator in the section entitled “Causal Effect Functions.” Methods for identifying parametrized causal quantities are discussed in the section entitled “Identification of Parametrized Causal Quantities”. Estimators of causal quantities that are consistent and converge at a rate of $N^{-1/2}$ are proposed in the section on "Estimation of Causal Quantities." We show that the proposed estimators are asymptotically efficient in case of maximum likelihood estimation.

Our work extends the literature on traditional SEM by providing closed-form expressions of graph-based causal quantities in terms of model parameters of linear SEM. Furthermore, we extend the literature on linear graph-based models by providing a unifying estimation framework for (multivariate) causal quantities that also allows estimation of causal quantities beyond the mean and the variance. We illustrate the method using simulated data based on an empirical application and provide a thorough discussion of the differences between conditional and interventional distributions in the illustration section.

Throughout this paper, we focus on situations in which direct causal effects are functionally independent of the values of variables in the system. In other words, direct causal effects are constant. In such situations, the data-generating mechanisms can be adequately represented by linear structural equations and the use of *linear* graph-based causal models is justified. A priori knowledge that suggests constant direct causal effects sometimes allows identifying causal quantities that would not be identified under the more flexible assumptions of the NPSEM (see illustration section for an example). However, scientific theories that suggest *constant* direct causal effects might be incorrect and consequently, linear models might be misspecified. We will discuss issues related to model misspecification in the discussion section, where we will also point to future research directions.

## Graph-Based Causal Models with Linear Equations

Linear graph-based causal models are an appropriate tool in situations in which a priori scientific knowledge suggests that each of the following statements is true:^3^

1. The causal ordering of observed variables and unobserved confounders is known.
2. Interventions only alter the mechanisms that are directly targeted (modularity).^4^
3. The treatment status of a unit (e.g., person) does not affect the treatment status or the outcome of other units (no interference).
4. Direct causal effects are constant across units (homogeneity).
5. Direct causal effects are constant across value combinations of observed variables and unobserved error terms (no effect modification).
6. Omitted direct causes as comprised in the error terms follow a multivariate normal distribution.^5^

The first three assumptions listed above are generic to the graph-based approach to causal inference and need to hold in its most general nonparametric formulation. Assumptions 4 and 5 justify the use of linear structural equations. Assumptions 6 justifies the use of multivariate normally distributed error terms. We further assume that variables are measured on a continuous scale and are observed without measurement error. Throughout this paper, we assume that the model is correctly specified. In the discussion section, we briefly point to the literature on statistical tests of model assumptions and methods for analyzing the sensitivity of causal conclusions with respect to violations of untestable assumptions. Furthermore, we briefly discuss possible ways to relax the model assumptions (e.g., measurement errors, unobserved heterogeneity, effect modification, excess kurtosis in the error terms).

A linear graph-based causal models over the set ${\mathcal {V}}=\{V_1,...,V_n\}$ of observed variables are defined by the following set of equations (Brito & Pearl, 2006, p.2):^6^

1 $$\begin{aligned} V_j=\sum _i^nc_{ji}V_i+\varepsilon _j, \quad j=1,...,n \end{aligned}$$

We assume that all variables are deviations from their means and no intercepts are included in Eq. (1). A nonzero structural coefficient ($c_{ji}\ne 0$) expresses the assumption that $V_i$ has a direct causal influence on $V_j$. Restricting a structural coefficient to zero ($c_{ji}=0$) indicates the assumption that $V_i$ has *no* direct causal effect on $V_j$. The parameter $c_{ji}$ quantifies the magnitude of a direct effect. The $q \times 1$ parameter vector ${\varvec{\theta }}_{{\mathcal {F}}}\in {\varvec{\Theta }}_{{\mathcal {F}}}\subseteq {\mathbb {R}}^q$ contains all distinct, functionally unrelated and unknown structural coefficients $c_{ji}$. ${\varvec{\Theta }}_{{\mathcal {F}}}$ denotes the parameter space, and it is a subspace of the *q*-dimensional Euclidean space. Restating Eqs. (1) in matrix notation yields:

2 $$\begin{aligned} {\mathbf {V}}={\mathbf {C}}{\mathbf {V}}+{\varvec{\varepsilon }} \quad \Leftrightarrow \quad {\mathbf {V}}&=({\mathbf{I}}_n-{\mathbf {C}})^{-1}{\varvec{\varepsilon }} \end{aligned}$$

The $n \times n$ identity matrix is denoted as ${\mathbf{I}}_n$. The $n \times n$ matrix of structural coefficients is denoted as ${\mathbf {C}}$, and we sometimes use the notation ${\mathbf {C}}({\varvec{\theta }}_{{\mathcal {F}}})$ to emphasize that ${\mathbf {C}}$ is a function of ${\varvec{\theta }}_{{\mathcal {F}}}$. We restrict our attention to recursive systems for which the variables ${\mathbf {V}}$ can be ordered in such a way that the matrix ${\mathbf {C}}$ is strictly lower triangular (which ensures the existence of the inverse in Eq. (2); Bollen, 1989). The set of error terms is denoted by ${\mathcal {E}}=\{\varepsilon _1,...,\varepsilon _n\}$. Each error term $\varepsilon _i$, $i=1,...,n$, comprises variables that determine the level of $V_i$ but are not explicitly included in the model. Typically the following assumptions (or a subset thereof) are made (Brito & Pearl, 2002; Kang & Tian, 2009; Koster, 1999):

- $\mathrm {E}({\varvec{\varepsilon }})={\mathbf {0}}_n$, where ${\mathbf {0}}_n$ is an $n\times 1$ vector that contains only zeros.
- $\mathrm {E}({\varvec{\varepsilon }}{\varvec{\varepsilon }}^\intercal )={\varvec{\Psi }}$, where the $n\times n$ matrix ${\varvec{\Psi }}$ is finite, symmetric and positive definite.
- ${\varvec{\varepsilon }}\sim {{N}}_n({\mathbf {0}}_n,{\varvec{\Psi }})$, where ${{N}}_n$ denotes the *n*-dimensional normal distribution.

A nonzero covariance $\psi _{ij}$ indicates the existence of an unobserved common cause of the variables $V_i$ and $V_j$. The $p \times 1$ parameter vector ${\varvec{\theta }}_{{\mathcal {P}}}\in {\varvec{\Theta }}_{{\mathcal {P}}}\subseteq {\mathbb {R}}^p$ contains all distinct, functionally unrelated and unknown parameters from the error term distribution. ${\varvec{\Theta }}_{{\mathcal {P}}}$ denotes the parameter space, and it is a subspace of the *p*-dimensional Euclidean space. We sometimes use the notation ${\varvec{\Psi }}({\varvec{\theta }}_{{\mathcal {P}}})$ to emphasize that ${\varvec{\Psi }}$ is a function of ${\varvec{\theta }}_{{\mathcal {P}}}$. The resulting model implied joint distribution of the observed variables is denoted by $\{P({\mathbf {v}},{\varvec{\theta }})\mid {\varvec{\theta }} \in {\varvec{\Theta }}\}$, where ${\varvec{\Theta }}={\varvec{\Theta }}_{{\mathcal {F}}}\times {\varvec{\Theta }}_{{\mathcal {P}}}$, and *P* is the family of *n*-dimensional multivariate normal distributions.

The graph ${\mathcal {G}}$ is constructed by drawing a directed edge from $V_i$ pointing to $V_j$ if and only if the corresponding coefficient is not restricted to zero (i.e., $c_{ji}\ne 0$). A bidirected edge between vertices $V_i$ and $V_j$ is drawn if and only if $\psi _{ij}\ne 0$ (bidirected edges are often drawn using dashed lines). The absence of a bidirected edge between $V_i$ and $V_j$ reflects the assumption that there is no unobserved variable that has a direct causal effect on both $V_i$ and $V_j$ (no unobserved confounding).^7^ For recursive systems, the resulting graph belongs to the class of acyclical directed *mixed* graphs (ADMG), whereas *mixed* refers to the fact that graphs in this class contain directed edges as well as bidirected edges (Richardson, 2003; Shpitser, 2018). An example model with $n=6$ variables and the corresponding causal graph is introduced in the illustration section.

At the heart of the graph-based approach to causal inference lies a hypothetical experiment in which the values of a subset of observed variables are controlled by an intervention. This exogenous intervention is formally denoted via the *do*-operator, namely $do({\mathbf {x}})$, where ${\mathbf {x}}$ denotes the interventional levels and ${\mathcal {X}}\subseteq {\mathcal {V}}$ denotes the subset of variables that are controlled by the experimenter. The system of equation under the intervention $do({\mathbf {x}})$ is obtained from the original system by replacing the equation for each variable $V_i\in {\mathcal {X}}$ (i.e., for each variable that is subject to the $do({\mathbf {x}})$-intervention) with the equation , where  is a constant interventional level (Pearl, 2009; Spirtes et al., 2001). Note that the $do({\mathbf {x}})$-intervention does not alter the equations for variables that are *not* subject to intervention, an assumption known as autonomy or modularity (Pearl, 2009; Peters, Janzing, & Schölkopf, 2017; Spirtes et al., 2001).

The probability distribution of the variables ${\mathbf {V}}$ one would observe had the intervention $do({\mathbf {x}})$ been uniformly applied to the entire population is called the interventional distribution, and it is denoted as $P({\mathbf {V}}\mid do({\mathbf {x}}))$.^8^ The interventional distribution differs formally and conceptually from the conditional distribution $P({\mathbf {V}}\mid {\mathbf {X}}={\mathbf {x}})$. The former describes a situation where the data-generating mechanism has been altered by an external $do({\mathbf {x}})$-type intervention in an (hypothetical) experiment. The latter describes a situation where the data-generating mechanism of ${\mathbf {V}}$ has *not* been altered, but the evidence ${\mathbf {X}}={\mathbf {x}}$ about the values of a subset of variables ${\mathcal {X}}\subseteq {\mathcal {V}}$ is available. These differences will be further discussed in the illustration section (see also, e.g., Gische, West, & Voelkle, 2021; Pearl, 2009).

In the remainder of this section, we translate the changes in the data-generating mechanism induced by the $do({\mathbf {x}})$-intervention into matrix notation (see Hauser and Bühlmann (2015) for a similar approach). The following definition introduces the required notation.

### Definition 1

(interventions in linear graph-based models)

1. Variables ${\mathcal {X}}\subseteq {\mathcal {V}}$ are subject to an external intervention, where $|{\mathcal {X}}|=K_x \le n$ denotes the set size. The $K_x\times 1$ vector of interventional levels is denoted by ${\mathbf {x}}$. The external intervention is denoted by $do({\mathbf {x}})$.
2. Let ${\mathcal {I}} \subseteq \{1,2,...,n\},\, |{\mathcal {I}}|=K_x$ denote the index set of variables that are subject to intervention. The index set of all variables that are *not* subject to intervention is denoted by ${\mathcal {N}}$, namely ${\mathcal {N}}:=\{1,2,...,n\}\setminus {\mathcal {I}},\, |{\mathcal {N}}|=n-K_x$, where the operator $\setminus$ denotes the set complement.
3. Let $\varvec{{{\imath }}}_i \in {\mathbb {R}}^n$ be the *i*-th unit vector, namely a (column) vector with entry 1 on the *i*-th component and zeros elsewhere. The $n \times K_x$ matrix ${\mathbf {1}}_{{\mathcal {I}}}:=(\varvec{{{\imath }}}_i)_{i\in {\mathcal {I}}}$ contains all unit vectors with an interventional index. The $n \times (n-K_x)$ matrix ${\varvec{1}}_{{\mathcal {N}}}$ is defined analogously, namely ${\mathbf {1}}_{{\mathcal {N}}}:=(\varvec{{{\imath }}}_i)_{i\in {\mathcal {N}}}$. The matrices ${\mathbf {1}}_{{\mathcal {I}}}$ and ${\mathbf {1}}_{{\mathcal {N}}}$ are called selection matrices.
4. Let ${\mathbf{I}}_ {{\mathcal {N}}}$ be an $n \times n$ diagonal matrix with zeros and ones as diagonal values. The *i*-th diagonal value is equal to one if $i \in {\mathcal {N}}$ and zero otherwise.

Note that all of the elements of the matrices ${\mathbf {1}}_{{\mathcal {I}}},{\mathbf {1}}_{{\mathcal {N}}},$ and ${\mathbf{I}}_ {{\mathcal {N}}}$ are either zero or unity. The variables ${\mathbf {V}}$ in a linear graph-based model under the intervention $do({\mathbf {x}})$ are determined by the following set of structural equations:^9^

3 $$\begin{aligned} \text {given} \ do({\mathbf {x}}):\quad {\mathbf {V}}&={\mathbf{I}}_ {{\mathcal {N}}}{\mathbf {C}}{\mathbf {V}}+{\mathbf{I}}_ {{\mathcal {N}}}{\varvec{\varepsilon }}+{\mathbf {1}}_{{\mathcal {I}}}{\mathbf {x}} \end{aligned}$$

The corresponding interventional reduced form equation is given by:

4 $$\begin{aligned} {\mathbf {V}}\mid do({\mathbf {x}})&=({\mathbf{I}}_ {n}-{\mathbf{I}}_ {{\mathcal {N}}}{\mathbf {C}})^{-1}({\mathbf{I}}_ {{\mathcal {N}}}{\varvec{\varepsilon }}+{\mathbf {1}}_{{\mathcal {I}}} {\mathbf {x}})=\underbrace{({\mathbf{I}}_n-{\mathbf{I}}_ {{\mathcal {N}}}{\mathbf {C}})^{-1}{\mathbf{I}}_ {{\mathcal {N}}}}_{=:{\mathbf {T}}_1 \ \ n \times n}{\varvec{\varepsilon }}+\underbrace{({\mathbf{I}}_n-{\mathbf{I}}_ {{\mathcal {N}}}{\mathbf {C}})^{-1} {\mathbf {1}}_{{\mathcal {I}}}}_{=:{\mathbf {a}}_1 \ \ n \times K_x}{\mathbf {x}} \end{aligned}$$

The matrix ${\mathbf{I}}_ {{\mathcal {N}}}{\mathbf {C}}$ is obtained from ${\mathbf {C}}$ by replacing its rows with interventional indexes by rows of zeros, and consequently $({\mathbf{I}}_ {n}-{\mathbf{I}}_ {{\mathcal {N}}}{\mathbf {C}})$ is non-singular. Equation (4) states that ${\mathbf {V}}\mid do({\mathbf {x}})$ is a linear transformation of the random vector ${\varvec{\varepsilon }}$. The corresponding transformation matrix is labeled as ${\mathbf {T}}_1$, and the additive constant is ${\mathbf {a}}_1{\mathbf {x}}$.

The target quantity of interest is the interventional distribution of those variables that are *not* subject to intervention, denoted by ${\mathbf {V}}_{{\mathcal {N}}}$. The reduced form equation of all non-interventional variables is given by:

5 $$\begin{aligned} {\mathbf {V}}_{{\mathcal {N}}}\mid do({\mathbf {x}})={\mathbf {1}}_{{\mathcal {N}}}^{\intercal }{\mathbf {V}}\mid do({\mathbf {x}})={\mathbf {1}}_{{\mathcal {N}}}^{\intercal } ({\mathbf {T}}_1{\varvec{\varepsilon }}+{\mathbf {a}}_1{\mathbf {x}}) =\underbrace{{\mathbf {1}}_{{\mathcal {N}}}^{\intercal } {\mathbf {T}}_1}_{=:{\mathbf {T}}_2}{\varvec{\varepsilon }} +\underbrace{{\mathbf {1}}_{{\mathcal {N}}}^{\intercal } {\mathbf {a}}_1}_{=:{\mathbf {a}}_2}{\mathbf {x}} \end{aligned}$$

Important characteristics of the distribution of a linear transformation of a random vector depend on the rank of the transformation matrix.

### Lemma 2

(rank of transformation matrices) The $n \times n$ transformation matrix ${\mathbf {T}}_1:=({\mathbf{I}}_n-{\mathbf{I}}_ {{\mathcal {N}}}{\mathbf {C}})^{-1}{\mathbf{I}}_ {{\mathcal {N}}}$ has reduced rank $n-K_x$. The $(n-K_x)\times n$ transformation matrix ${\mathbf {T}}_2:={\mathbf {1}}_{{\mathcal {N}}}^{\intercal }({\mathbf{I}}_n-{\mathbf{I}}_ {{\mathcal {N}}}{\mathbf {C}})^{-1}{\mathbf{I}}_ {{\mathcal {N}}}$ has full row rank $n-K_x$.

### Proof

See Appendix. $$\square $$

Based on the reduced form equations, we derive the interventional distribution and its features in the following section.

## Interventional Distribution

Combining the reduced form stated in Eq. (4) with the assumptions on the first- and second-order moments of the error term distribution yields the following moments of the interventional distribution:

6a $$\begin{aligned} \mathrm {E}({\mathbf {V}} \mid do({\mathbf {x}}))&=\mathrm {E}({\mathbf {T}}_1\mathbf {{\varvec{\varepsilon }}} +{\mathbf {a}}_1{\mathbf {x}})={\mathbf {a}}_1{\mathbf {x}} =({\mathbf{I}}_n-{\mathbf{I}}_{{\mathcal {N}}}{\mathbf {C}})^{-1}{\mathbf {1}}_{{\mathcal {I}}} {\mathbf {x}} \end{aligned}$$

6b $$\begin{aligned} \mathrm {V}({\mathbf {V}}\mid do({\mathbf {x}}))&=\mathrm {V}({\mathbf {T}}_1{\varvec{\varepsilon }} +{\mathbf {a}}_1{\mathbf {x}})={\mathbf {T}}_1{\varvec{\Psi }} {\mathbf {T}}_1^\intercal =({\mathbf{I}}_n-{\mathbf{I}}_ {{\mathcal {N}}}{\mathbf {C}})^{-1}{\mathbf{I}}_ {{\mathcal {N}}}{\varvec{\Psi }}{\mathbf{I}}_ {{\mathcal {N}}}({\mathbf{I}}_n-{\mathbf{I}}_ {{\mathcal {N}}}{\mathbf {C}})^{-\intercal } \end{aligned}$$

The results are obtained via a direct application of the rules for the computation of moments of linear transformations of random variables. Note that these results do *not* require multivariate normality of the error terms. The interventional mean vector is functionally dependent on the vector of interventional levels ${\mathbf {x}}$, whereas the interventional covariance matrix is functionally independent of ${\mathbf {x}}$. The interventional distribution in linear graph-based models with multivariate normal error terms is given as:

### Result 3

(interventional distribution for Gaussian linear graph-based models)

7a $$\begin{aligned} {\mathbf {V}} \mid do({\mathbf {x}})&\sim {{N}}^{n-K_x}_n (\ {\mathbf {a}}_1{\mathbf {x}} \ , \ {\mathbf {T}}_1{\varvec{\Psi }} {\mathbf {T}}_1^{\intercal } \ ) \end{aligned}$$

7b $$\begin{aligned} {\mathbf {V}}_{{\mathcal {N}}} \mid do({\mathbf {x}})&\sim {{N}}_{n-K_x} \quad ( \ {\mathbf {a}}_2{\mathbf {x}} \ , \ {\mathbf {T}}_2{\varvec{\Psi }}{\mathbf {T}}_2^{\intercal } \ ) \end{aligned}$$

### Proof

Both results follow from the fact that linear transformations of multivariate normal vectors are also multivariate normal (Rao, 1973). Results on the rank of the transformation matrices ${\mathbf {T}}_1$ and ${\mathbf {T}}_2$ can be found in Lemma 2. $\square$

Equation (7a) states that the interventional distribution of all variables is a singular normal distribution in ${\mathbb {R}}^n$ with reduced rank $n-K_x$ as denoted by the superscript $n-K_x$. Singularity follows from the fact that the $K_x$ interventional variables are no longer random given the $do({\mathbf {x}})$-intervention, but are fixed to the constant interventional levels ${\mathbf {x}}$. Therefore, the random vector ${\mathbf {V}}\mid do({\mathbf {x}})$ satisfies the restriction ${\mathbf {1}}_{{\mathcal {I}}}^{\intercal }({\mathbf {V}}\mid do({\mathbf {x}}))={\mathbf {x}}$ with a probability of one. Equation (7b) states that the vector of all non-interventional variables follows a $(n-K_x)$-dimensional normal distribution.

Typically, one is interested in a subset ${\mathcal {Y}}\subseteq {\mathcal {V}}_{{\mathcal {N}}}$ of outcome variables. The marginal interventional distribution $P({\mathbf {y}}\mid do({\mathbf {x}}))$ can be obtained as follows:

### Result 4

(marginal interventional distribution for Gaussian linear graph-based models) Let the outcome variables ${\mathcal {Y}}$ be a subset of the non-interventional variables (i.e., ${\mathcal {Y}}\subseteq {\mathcal {V}}_{{\mathcal {N}}}, \, |{\mathcal {Y}}|=K_y$). The index set of the outcome variables is denoted as ${\mathcal {I}}_y$. Then, the following result holds:

8 $$\begin{aligned} P({\mathbf {y}}\mid do({\mathbf {x}}))&\sim {N}_{K_y} ( \ {\mathbf {1}}_{{\mathcal {I}}_y}^\intercal {\mathbf {a}}_1{\mathbf {x}} \ , \ {\mathbf {1}}_{{\mathcal {I}}_y}^\intercal {\mathbf {T}}_1 {\varvec{\Psi }}{\mathbf {T}}_1^{\intercal }{\mathbf {1}}_{{\mathcal {I}}_y} \ ) \end{aligned}$$

The result follows from the fact that the family of multivariate normal distributions is closed with respect to marginalization (Rao, 1973). An important special case of Result 4 is the ATE of a single variable $V_i$ on another variable $V_j$, which is obtained by the setting ${\mathcal {Y}}=\{V_j\}$ and ${\mathcal {X}}=\{V_i\}$ (and consequently ${\mathcal {I}}_x=\{i\}$, ${\mathcal {I}}_y=\{j\}$, $K_y=K_x=1$). The ATE of the intervention *do*(*x*) relative to the intervention $do(x')$ (where *x* and $x'$ are distinct treatment levels) on *Y* is defined as the mean difference $\mathrm {E}(y\mid do(x))-\mathrm {E}(y\mid do(x'))$. For a single outcome variable $\{V_j\}$, the selection matrix ${\mathbf {1}}_{{\mathcal {I}}_y}$ simplifies to the unit vector $\varvec{{{\imath }}}_j$ and $\mathrm {E}(y\mid do(x))-\mathrm {E}(y\mid do(x'))$ can be expressed as $\varvec{{{\imath }}}_j^\intercal {\mathbf {a}}_1(x-x')$ (using the mean expression from the normal distribution in Eq. [8]).

The probability density function (pdf) of the interventional distribution of all non-interventional variables is given as follows:

9 $$\begin{aligned} f({\mathbf {v}}_{{\mathcal {N}}}\mid do({\mathbf {x}}))=(2\pi )^{-\frac{n-K_x}{2}}|{\mathbf {T}}_{2} {\varvec{\Psi }}{\mathbf {T}}_{2}|^{-\frac{1}{2}}\exp \left( -\frac{1}{2}({\mathbf {v}}_{{\mathcal {N}}} -{\mathbf {a}}_{2}{\mathbf {x}})^{\intercal }({\mathbf {T}}_{2} {\varvec{\Psi }}{\mathbf {T}}_{2}^{\intercal })^{-1}({\mathbf {v}}_{{\mathcal {N}}} -{\mathbf {a}}_{2}{\mathbf {x}})\right) \end{aligned}$$

Many features of the interventional distribution that hold substantive interest in applied research (e.g., probabilities of interventional events, quantiles of the interventional distribution) can be calculated from the pdf via integration. For example, a physician would like a patient’s blood glucose level (outcome) to fall into a predefined range of values (e.g., to avoid hypo- or hyperglycemia) given an injection of insulin (intervention). More formally, let $[{\mathbf {y}}^{\textit{low}},{\mathbf {y}}^{\textit{up}}]$ denote a predefined range of values of a set of outcome variables ${\mathcal {Y}}\subseteq {\mathcal {V}}_{{\mathcal {N}}}$. The interventional probability $P({\mathbf {y}}^{{low}}\le {\mathbf {y}} \le {\mathbf {y}}^{{up}} \mid do({\mathbf {x}}))$ is given by:

10 $$\begin{aligned} P({\mathbf {y}}^{{low}}\le {\mathbf {y}} \le {\mathbf {y}}^{{up}} \mid do({\mathbf {x}})) =\int _{{\mathbf {y}}^{{low}}}^{{\mathbf {y}}^{{up}}} f({\mathbf {y}}\mid do({\mathbf {x}})) \textsf {d}{\mathbf {y}} \end{aligned}$$

The interventional distribution and its features will be used to formally define parametric causal quantities in the following section.

## Causal Effect Functions

**Fig. 1:**
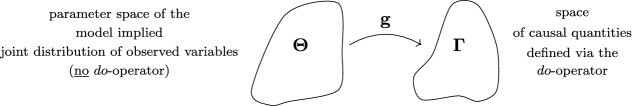
*Causal Effect Functions*. Figure 1 displays the mapping ${\mathbf {g}}:{\varvec{\Theta }}\mapsto {\varvec{\Gamma }}$ that corresponds to a causal effect function ${\varvec{\gamma }}={\mathbf {g}}({\varvec{\theta }})$. The domain ${\varvec{\Theta }}\subseteq {\mathbb {R}}^{q+p}$ (left-hand side) contains the parameters of the model implied joint distribution of observed variables (no *do*-operator). The co-domain ${\varvec{\Gamma }}\subseteq {\mathbb {R}}^{r}$ (right-hand side) contains causal quantities ${\varvec{\gamma }}$ that are defined via the *do*-operator.

In this section, we formally define terms containing the *do*-operator as *causal quantities* denoted by ${\varvec{\gamma }}$. According to this definition, any feature of the interventional distribution that can be expressed using the *do*-operator is a causal quantity. Let the space of causal quantities be denoted as ${\varvec{\Gamma }}$. As discussed in earlier in the section on “Graph-Based Causal Models with Linear Equations,” linear causal models imply a joint distribution of observed variables that is parametrized by ${\varvec{\theta }}\in {\varvec{\Theta }}\subseteq {\mathbb {R}}^{q+p}$ and denoted by $\{P({\mathbf {v}},{\varvec{\theta }})\mid {\varvec{\theta }} \in {\varvec{\Theta }}\}$. A function ${\mathbf {g}}$ that maps the parameters ${\varvec{\theta }}$ of the model implied joint distribution onto a causal quantity ${\varvec{\gamma }}$ is called *causal effect function*. This idea is illustrated in Fig. 1 and stated in Definition 5.

### Definition 5

(causal quantity and causal effect function) Let ${\varvec{\gamma }}$ be an *r*-dimensional feature of the interventional distribution. Let ${\varvec{\Theta }}_{\varvec{\gamma }}\subseteq {\varvec{\Theta }}$ be an *s*-dimensional subspace of the parameter space of the model implied joint distribution of observed variables. A mapping ${\mathbf {g}}$

11 $$\begin{aligned} {\mathbf {g}}: {\varvec{\Theta }}_{{\varvec{\gamma }}} \mapsto {\mathbb {R}}^{r}, \quad \text {with} \ {\varvec{\gamma }}={\mathbf {g}}({\varvec{\theta }}_{\varvec{\gamma }}), \quad {\varvec{\theta }}_{\varvec{\gamma }}\in {\varvec{\Theta }}_{\varvec{\gamma }}\subseteq {\mathbb {R}}^s, {\varvec{\gamma }}\in {\mathbb {R}}^{r} \end{aligned}$$

is called a causal effect function. The image ${\varvec{\gamma }}$ of a causal effect function is called a causal quantity which is parametrized by ${\varvec{\theta }}_{\varvec{\gamma }}$. If the value of a causal quantity depends on other variables (e.g., the interventional level ${\mathbf {x}}\in {\mathbb {R}}^{K_x}$, the values ${\mathbf {v}}_{{\mathcal {N}}}\in {\mathbb {R}}^{n-K_x}$ of non-interventional variables), we include these variables as auxiliary arguments in the causal effect function separated by a semicolon (e.g., ${\mathbf {g}}({\varvec{\theta }}_{\varvec{\gamma }};{\mathbf {x}}, {\mathbf {v}}_{{\mathcal {N}}})$).

This idea can be applied to the interventional mean from Eq. (6a) by defining it as a causal quantity ${\varvec{\gamma }}_1$ as follows:

12a $$\begin{aligned} {\varvec{\gamma }}_1&:=\mathrm {E}({\mathbf {V}} \mid do({\mathbf {x}})) ={\mathbf {g}}_1({\varvec{\theta }}_{{\mathcal {F}}};{\mathbf {x}})=({\mathbf{I}}_n -{\mathbf{I}}_{{\mathcal {N}}}{\mathbf {C}}({\varvec{\theta }}_{{\mathcal {F}}}))^{-1} {\mathbf {1}}_{{\mathcal {I}}}{\mathbf {x}} \end{aligned}$$

12b $$\begin{aligned} {\mathbf {g}}_1&:{\varvec{\Theta }} \supseteq {\varvec{\Theta }}_{{\mathcal {F}}}\mapsto {\mathbb {R}}^{n}\subseteq {\varvec{\Gamma }} \end{aligned}$$

The right-hand side of Eq. (12a) is free of the *do*-operator and contains the parameter vector ${\varvec{\theta }}_{{\mathcal {F}}}$ (structural coefficients ) as a main argument and the interventional level ${\mathbf {x}}$ as an auxiliary argument. Thus, the causal effect function ${\mathbf {g}}_1$ maps the parameter vector ${\varvec{\theta }}_{{\mathcal {F}}}$ onto the interventional mean. The interventional mean is an $n\times 1$ vector and therefore the co-domain of ${\mathbf {g}}_1$ is ${\mathbb {R}}^{n}$ (i.e., $r=n$), as stated in Eq. (12b). Note that the causal effect function ${\mathbf {g}}_1$ depends on the distinct and functionally unrelated structural coefficients ${\varvec{\theta }}_{{\mathcal {F}}}$ but is independent of the parameters from the error term distribution ${\varvec{\theta }}_{{\mathcal {P}}}$. Therefore, the domain of ${\mathbf {g}}_1$ is ${\varvec{\Theta }}_{{\mathcal {F}}}$ and $s=q$.

The interventional covariance matrix from Eq. (6b) can be expressed using the notation from Definition 5 as follows:

13 $$\begin{aligned} {\varvec{\gamma }}_2:&={{{\,\mathrm{\textsf {vech}}\,}}}(\mathrm {V}({\mathbf {V}} \mid do({\mathbf {x}})))={\mathbf {g}}_2({\varvec{\theta }})\nonumber \\&={{{\,\mathrm{\textsf {vech}}\,}}}\left( ({\mathbf{I}}_n-{\mathbf{I}}_{{\mathcal {N}}}{\mathbf {C}} ({\varvec{\theta }}_{{\mathcal {F}}}))^{-1}{\mathbf{I}}_{{\mathcal {N}}} {\varvec{\Psi }}({\varvec{\theta }}_{{\mathcal {P}}}){\mathbf{I}}_{{\mathcal {N}}} ({\mathbf{I}}_n-{\mathbf{I}}_{{\mathcal {N}}}{\mathbf {C}} ({\varvec{\theta }}_{{\mathcal {F}}}))^{-\intercal }\right) \end{aligned}$$

To avoid matrix valued causal effect functions, we defined ${\varvec{\gamma }}_2$ as the half-vectorized interventional covariance matrix, which is of dimension $r=n(n+1)/2$ (the operator ${{{\,\mathrm{\textsf {vech}}\,}}}$ stands for half-vectorization). The interventional covariance matrix is a function of both the structural coefficients ${\varvec{\theta }}_{{\mathcal {F}}}$ and the entries of the covariance matrix ${\varvec{\theta }}_{{\mathcal {P}}}$. Thus, ${\varvec{\theta }}_{{{\varvec{\gamma }}}_2}={\varvec{\theta }}$ and $s=q+p$. No auxiliary arguments are included in the causal effect function ${\mathbf {g}}_2$, since the value of ${\varvec{\gamma }}_2$ only depends on the values of ${\varvec{\theta }}$ (recall that ${\mathbf{I}}_n,{\mathbf{I}}_{{\mathcal {N}}},{\mathbf {1}}_{{\mathcal {I}}}$ are constant zero-one matrices).

The interventional pdf $f({\mathbf {v}}_{{\mathcal {N}}}\mid do({\mathbf {x}}))$ from Eq. (9) can be formally defined as a causal effect function as follows:

14 $$\begin{aligned} \gamma _3:=&g_3({\varvec{\theta }};{\mathbf {x}},{\mathbf {v}}_{{\mathcal {N}}}) =(2\pi )^{-\frac{n-K_x}{2}}|{\mathbf {T}}_{2}({\varvec{\theta }}_{{\mathcal {F}}}) {\varvec{\Psi }}({\varvec{\theta }}_{{\mathcal {P}}}){\mathbf {T}}_{2} ({\varvec{\theta }}_{{\mathcal {F}}})^{\intercal }|^{-\frac{1}{2}}\nonumber \\ \times&\exp \left( -\frac{1}{2}({\mathbf {v}}_{{\mathcal {N}}}-{\mathbf {a}}_{2} ({\varvec{\theta }}_{{\mathcal {F}}}){\mathbf {x}})^{\intercal }({\mathbf {T}}_{2} ({\varvec{\theta }}_{{\mathcal {F}}}){\varvec{\Psi }}({\varvec{\theta }}_{{\mathcal {P}}}) {\mathbf {T}}_{2}({\varvec{\theta }}_{{\mathcal {F}}})^{\intercal })^{-1} ({\mathbf {v}}_{{\mathcal {N}}}-{\mathbf {a}}_{2} ({\varvec{\theta }}_{{\mathcal {F}}}){\mathbf {x}})\right) \end{aligned}$$

The interventional density depends on both the structural coefficients and the parameters of the error term distribution, yielding ${\varvec{\theta }}_{\gamma _3}={\varvec{\theta }}$, ${\varvec{\Theta }}_{\gamma _3}={\varvec{\Theta }}$ and $s=q+p$. The interventional density is scalar-valued and thus $r=1$. Since the value of the interventional pdf depends on ${\mathbf {x}}$ and ${\mathbf {v}}_{{\mathcal {N}}}$, both are included as auxiliary arguments in the causal effect function $g_3$, namely $g_3({\varvec{\theta }};{\mathbf {x}},{\mathbf {v}}_{{\mathcal {N}}})$.

Probabilities of interventional events can be understood as a causal quantity in the following way:

15 $$\begin{aligned} \gamma _4:=P({\mathbf {y}}^{{low}}\le {\mathbf {y}} \le {\mathbf {y}}^{{up}} \mid do({\mathbf {x}}))=g_4({\varvec{\theta }}_{\gamma _4};{\mathbf {x}}, {\mathbf {y}}^{{low}},{\mathbf {y}}^{{up}}) =\int _{{\mathbf {y}}^{{low}}}^{{\mathbf {y}}^{{up}}} f({\mathbf {y}}\mid do({\mathbf {x}})) \textsf {d}{\mathbf {y}} \end{aligned}$$

Where ${\varvec{\theta }}_{\gamma _4}$ is the subset of parameters that appear in the marginal interventional pdf $f({\mathbf {y}}\mid do({\mathbf {x}}))$. The causal effect function $g_4$ is a scalar-valued and thus $r=1$. The value of the interventional probability depends on ${\mathbf {x}}$, ${\mathbf {y}}^{{low}}$, and ${\mathbf {y}}^{{up}}$ (${\mathbf {y}}$ integrates out), which are included as auxiliary arguments in the causal effect function $g_4$.

## Identification of Parametrized Causal Quantities

The meaning of the term “identification” as used in the nonparametric graph-based approach slightly differs from the meaning in the field of traditional SEM. A graph-based causal quantity is said to be identified if it can be expressed as a functional of joint, marginal, or conditional distributions of observed variables (Pearl, 2009). The latter distributions can in principle be estimated based on observational data using nonparametric statistical models. In other words, an identified nonparametric causal quantity could in theory be computed from an infinitely large sample without further limitations.^10^ Graph-based tools for identification exploit the causal structure depicted in the causal graph and are *independent* of the functional form of the structural equations. Thus, causal identification is established in the absence of the risk of misspecification of the functional form.

By contrast, model identification in traditional parametric SEM relies on the solvability of a system of nonlinear equations in terms of a finite number of model parameters. A single parameter $\theta \in {\varvec{\Theta }}$ is identified if it can be expressed as a function of moments of the the joint distribution of observed variables in a unique way (Bekker et al., 1994; Bollen & Bauldry, 2010). If all parameters in ${\varvec{\theta }}$ are identified, then the model is identified. Definition 6 uses causal effect functions to combine the above ideas.

### Definition 6

(causal identification of parametrized causal quantities) Let ${\varvec{\gamma }}$ be a *parametrized* causal quantity in a linear graph-based model. ${\varvec{\gamma }}$ is said to be causally identified if (i) it can be expressed in a unique way as a function of the parameter vector ${\varvec{\theta }}_{\varvec{\gamma }}$ via a causal effect function, namely ${\varvec{\gamma }}={\mathbf {g}}({\varvec{\theta }}_{\varvec{\gamma }})$, *and* (ii) the value of ${\varvec{\theta }}_{\varvec{\gamma }}$ can be uniquely computed from the joint distribution of the observed variables.

Based on this insight, graph-based techniques for causal identification in linear models have been derived, for example by Brito and Pearl (2006); Drton, Foygel, and Sullivant (2011); Kuroki and Cai (2007). Furthermore, part (ii) of the above definition has been dealt with extensively in the literature on traditional linear SEM (see, e.g., Bekker et al., 1994; Bollen, 1989; Fisher, 1966; Wiley, 1973).

We now illustrate Definition 6 for the causal quantities defined in Eqs. (22a) and (22b) from the illustration section. For the interventional mean stated in Eq. (22a), part (i) of the definition is satisfied, since the causal quantity $\gamma _1$ can be expressed as a function of the parameter ${\theta }_{\gamma _1}={c_{yx}}$ in a unique way as follows: $\gamma _{1}:=\mathrm{E}(Y_3 \mid do(x_2))=g_1({\theta }_{\gamma _1};x_2)=c_{yx}x_2$. Part (ii) of the above definition requires that the single structural coefficient $c_{yx}$ can be uniquely computed from the joint distribution of the observed variables.

Similarly, part (i) of the definition is satisfied for the the causal quantity $\gamma _{2}:=\mathrm{V}(Y_3 \mid do(x_2))=g_2({\varvec{\theta }}_{\gamma _2})$ in Eq. (22b). Part (ii) of the above definition requires that each of the structural coefficients and (co)variances on the right-hand side of Eq. (22b), namely ${\varvec{\theta }}_{\gamma _2}=(c_{yx},c_{yy},\psi _{x_1x_1},\psi _{x_1y_1},\psi _{y_1y_1},\psi _{y_1y_2},\psi _{y_2y_3},\psi _{yy})^\intercal$, can be uniquely computed from the joint distribution of the observed variables.

Note that both of the causal quantities discussed above require only a subset of parameters to be identified (i.e., it is not required to identify the entire model ${\varvec{\theta }}$). After causal identification of a parametrized causal quantity has been established, it can be estimated from a sample using the techniques described in the following section.

## Estimation of Causal Quantities

Estimators of causal quantities as defined in Eq. (11) are constructed by replacing the parameters in the causal effect function with a corresponding estimator, namely $\widehat{{\varvec{\gamma }}}={\mathbf {g}}(\widehat{{\varvec{\theta }}}_{\varvec{\gamma }})$. This plug-in procedure is summarized in the following definition.

### Definition 7

(estimation of parametrized causal quantities) Let ${\varvec{\gamma }}$ be an identified causal quantity in a linear graph-based models and ${\mathbf {g}}({\varvec{\theta }}_{\varvec{\gamma }})$ the corresponding causal effect function. Let $\widehat{{\varvec{\theta }}}_{\varvec{\gamma }}$ denote an estimator of ${\varvec{\theta }}_{\varvec{\gamma }}$, then $\widehat{{\varvec{\gamma }}}:={\mathbf {g}}(\widehat{{\varvec{\theta }}}_{\varvec{\gamma }})$ is an estimator of the causal quantity ${\varvec{\gamma }}$.

A main strength of the traditional SEM literature is that a variety of estimation procedures have been developed. Common estimation techniques include maximum likelihood (ML; Jöreskog, 1967; Jöreskog & Lawley, 1968), generalized least squares (GLS; Browne, 1974), and asymptotically distribution free (ADF; Browne, 1984).^11^ Note that some estimation techniques do *not* rely on the assumption of multivariate normal error terms and for others robust versions have been proposed that allow for certain types of deviations from multivariate normality (Satorra & Bentler, 1994; Yuan & Bentler, 1998).

In the following, we assume that causal effect functions ${\mathbf {g}}$ and estimators $\widehat{{\varvec{\theta }}}_{\varvec{\gamma }}$ satisfy certain regularity conditions stated as Properties A.1 and A.2 in the Appendix. The following theorem establishes the asymptotic properties of estimators of causal quantities $\widehat{{\varvec{\gamma }}}={\mathbf {g}}(\widehat{{\varvec{\theta }}}_{\varvec{\gamma }})$.

### Theorem 8

(asymptotic properties of estimators of causal quantities) Let ${\varvec{\gamma }}$ be a causal quantity and ${\mathbf {g}}({\varvec{\theta }}_{\varvec{\gamma }})$ the corresponding causal effect function. Let $\widehat{{\varvec{\theta }}}_{\varvec{\gamma }}$ be an estimator of ${\varvec{\theta }}_{\varvec{\gamma }}$. Assume that ${\mathbf {g}}$ and $\widehat{{\varvec{\theta }}}_{\varvec{\gamma }}$ satisfy Property A.1 and Property A.2, respectively.

16a $$\begin{aligned} \widehat{{\varvec{\gamma }}}={\mathbf {g}}(\widehat{{\varvec{\theta }}}_{\varvec{\gamma }})&\xrightarrow { \ p \ } {\mathbf {g}}({\varvec{\theta }}^*_{\varvec{\gamma }})={\varvec{\gamma }}^* \end{aligned}$$

16b $$\begin{aligned} \sqrt{N}\left( \widehat{{\varvec{\gamma }}}-{\varvec{\gamma }}^*\right)&\xrightarrow { \ d \ } {{N}}_{r} \big (\,{\mathbf {0}}_r\, , \,\mathrm{AV}(\sqrt{N}\widehat{{\varvec{\gamma }}})\,\big ) \end{aligned}$$

16c $$\begin{aligned} \text {with} \quad \mathrm{AV}(\sqrt{N}\widehat{{\varvec{\gamma }}})&:=\frac{\partial {\mathbf {g}}({\varvec{\theta }}_{\varvec{\gamma }})}{\partial {\varvec{\theta }}_{\varvec{\gamma }}^\intercal }\Bigr |_{{\varvec{\theta }}_{\varvec{\gamma }} ={\varvec{\theta }}_{\varvec{\gamma }}^*}\mathrm{AV}(\sqrt{N}\widehat{{\varvec{\theta }}}_{\varvec{\gamma }}) \frac{\partial {\mathbf {g}}({\varvec{\theta }}_{\varvec{\gamma }})}{\partial {\varvec{\theta }}_{\varvec{\gamma }}}\Bigr |_{{\varvec{\theta }}_{\varvec{\gamma }} ={\varvec{\theta }}_{\varvec{\gamma }}^*} \end{aligned}$$

Where ${\varvec{\theta }}_{{\varvec{\gamma }}}^*$ denotes the true population value and $\xrightarrow { \ p \ }$ ($\xrightarrow { \ d \ }$) refers to convergence in probability (distribution) as the sample size *N* tends to infinity. $\text {AV}(\sqrt{N}\widehat{{\varvec{\theta }}}_{\gamma })$ denotes the covariance matrix of the limiting distribution.

### Proof

The results are obtained via a straightforward application of standard results on transformations of convergent sequences of random variables (Mann & Wald, 1943; Serfling, 1980, Chapter 1.7), one of which is known as the multivariate delta method (Cramér, 1946; Serfling, 1980, Chapter 3.3). $\square$

Theorem 8 establishes that the estimator $\widehat{{\varvec{\gamma }}}={\mathbf {g}}(\widehat{{\varvec{\theta }}}_{\varvec{\gamma }})$ is consistent and converges at a rate of $N^{-\frac{1}{2}}$ to the true population value ${\varvec{\gamma }}^*\!\!={\mathbf {g}}({\varvec{\theta }}_{\varvec{\gamma }}^*)$. The rate of convergence is independent of the finite number of parameters and variables in the model. If the causal effect function contains auxiliary variables, then the results in Theorem 8 hold pointwise for any fixed value combination of the auxiliary variable.

Note that the results in Theorem 8 hold whenever an estimator satisfies Property A.2 and they do not depend on a particular estimation method. However, if ${\varvec{\theta }}_{\varvec{\gamma }}$ is estimated via maximum likelihood, the proposed estimator $\widehat{{\varvec{\gamma }}}$ of the causal quantity has the following property:

### Theorem 9

(asymptotic efficiency of $\widehat{{\varvec{\gamma }}}={\mathbf {g}}(\widehat{{\varvec{\theta }}}^{ML}_{\varvec{\gamma }})$) Let the situation be as in Theorem 8 and $\widehat{{\varvec{\theta }}}^{ML}_{\varvec{\gamma }}$ denote the maximum likelihood estimator of ${\varvec{\theta }}_{\varvec{\gamma }}$. Then, the estimator $\widehat{{\varvec{\gamma }}}={\mathbf {g}}(\widehat{{\varvec{\theta }}}^{ML}_{\varvec{\gamma }})$

(i) is the maximum likelihood estimator $\widehat{{\varvec{\gamma }}}^{ML}$ of the causal quantity ${\varvec{\gamma }}$;
(ii) is asymptotically efficient, namely the asymptotic covariance matrix AV$(\sqrt{N}\widehat{{\varvec{\gamma }}})$ reaches the Cramér–Rao lower bound.

### Proof

Result (i) is a direct consequence of the functional invariance of the ML-estimator (Zehna, 1966; see, for example, Casella & Berger, 2002, Chapter 7.2) and result (ii) was established by Cramér (1946) and Rao (1945). $\square$

To make inference feasible in practical applications, a consistent estimator of $\mathrm{AV}(\sqrt{N}\widehat{{\varvec{\gamma }}})$ is required.

### Corollary 10

(consistent estimator of $\mathrm{AV}(\sqrt{N}\widehat{{\varvec{\gamma }}})$) Let the situation be as in Theorem 8 and let the estimator of AV$(\sqrt{N}\widehat{{\varvec{\gamma }}})$ be defined as:

17 $$\begin{aligned} {\widehat{\mathrm{AV}}}(\sqrt{N}\widehat{{\varvec{\gamma }}})&:=\frac{\partial {\mathbf {g}}({\varvec{\theta }}_{\varvec{\gamma }})}{\partial {\varvec{\theta }}_{\varvec{\gamma }}^\intercal }\Bigr |_{{\varvec{\theta }}_{\varvec{\gamma }} =\widehat{{\varvec{\theta }}}_{\varvec{\gamma }}}{\widehat{\mathrm{AV}}}(\sqrt{N} \widehat{{\varvec{\theta }}}_{\varvec{\gamma }})\frac{\partial {\mathbf {g}}({\varvec{\theta }}_{\varvec{\gamma }})}{\partial {\varvec{\theta }}_{\varvec{\gamma }}}\Bigr |_{{\varvec{\theta }}_{\varvec{\gamma }} =\widehat{{\varvec{\theta }}}_{\varvec{\gamma }}} \end{aligned}$$

Then, ${\widehat{\mathrm{AV}}}(\sqrt{N}\widehat{{\varvec{\gamma }}})$ is a consistent estimator of AV$(\sqrt{N}\widehat{{\varvec{\gamma }}})$ if ${\widehat{\mathrm{AV}}}(\sqrt{N}\widehat{{\varvec{\theta }}}_{\varvec{\gamma }}) \xrightarrow { \ p \ } {\mathrm{AV}}(\sqrt{N}\widehat{{\varvec{\theta }}}_{\varvec{\gamma }})$.

### Proof

Note that the partial derivatives $\frac{\partial {\mathbf {g}}({\varvec{\theta }}_{\varvec{\gamma }})}{\partial {\varvec{\theta }}_{\varvec{\gamma }}^\intercal }$ are continuous (see Property A.1) and that $\widehat{{\varvec{\theta }}}_{\varvec{\gamma }}\xrightarrow { \ p \ }{\varvec{\theta }}^*_{\varvec{\gamma }}$ holds (see Property A.2). Thus, the result is a direct consequence of standard results on transformations of convergent sequences of random variables (Mann & Wald, 1943; Serfling, 1980, Chapter 1.7). $\square$

Equation (17) states that estimates of the asymptotic covariance matrix of a causal quantity $\widehat{{\varvec{\gamma }}}$ can be computed based on (i) the estimate of the asymptotic covariance matrix ${\widehat{\text{ AV }}}(\sqrt{N}\widehat{{\varvec{\theta }}}_{\varvec{\gamma }})$, and (ii) the Jacobian matrix $\frac{\partial {\mathbf {g}}({\varvec{\theta }}_{\varvec{\gamma }}\!)}{\partial {\varvec{\theta }}^{\intercal }_{\varvec{\gamma }}}$ (evaluated at $\widehat{{\varvec{\theta }}}_{\varvec{\gamma }}$). Estimation results for (i) the asymptotic covariance matrix depend on the estimation method that is used to obtain $\widehat{{\varvec{\theta }}}_{\varvec{\gamma }}$. For many standard procedures (e.g., 3SLS, ADF, GLS, GMM, ML, IV), theoretical results on the asymptotic covariance matrix are available in the corresponding literature and estimators are implemented in various software packages (e.g., see Muthén & Muthén, 1998-2017; Rosseel, 2012). Explicit expressions of (ii) the Jacobian matrices for the causal effect functions $\mathbf {g_1}$, $\mathbf {g_2}$, $g_3$, and $g_4$ are provided in the following corollary.

### Corollary 11

(Jacobian matrices of basic causal effect functions) Let the causal effect functions ${\mathbf {g}}_1$, ${\mathbf {g}}_2$, $g_3$, and $g_4$ be defined as in Eqs. (12a), (13), (14), and (15), respectively. Then, the Jacobian matrices with respect to ${\varvec{\theta }}$ are given by:

18a $$\begin{aligned} \frac{\partial {\mathbf {g}}_1({\varvec{\theta }}_{{{\varvec{\gamma }}}_1};{\mathbf {x}})}{\partial {\varvec{\theta }}^\intercal }&=\big (({\mathbf {x}}^\intercal {\mathbf {1}}_{{\mathcal {{I}}}}^\intercal ({\mathbf{I}}_n-{\mathbf{I}}_{{\mathcal {N}}}{\mathbf {C}})^{-\intercal })\otimes (({\mathbf{I}}_n -{\mathbf{I}}_{{\mathcal {N}}}{\mathbf {C}})^{-1}{\mathbf{I}}_{{\mathcal {N}}}))\big ) \frac{\partial {{{\,\mathrm{\textsf {vec}}\,}}} {\mathbf {C}}}{\partial {\varvec{\theta }}^\intercal } \end{aligned}$$

18b $$\begin{aligned} \frac{\partial {\mathbf {g}}_2({\varvec{\theta }}_{{{\varvec{\gamma }}}_2})}{\partial {\varvec{\theta }}^\intercal }&={\mathbf {L}}_n\big [{\mathbf {G}}_{2,\mathbf{C}} \frac{\partial {{{\,\mathrm{\textsf {vec}}\,}}}{{\mathbf {C}}}}{\partial {\varvec{\theta }}^\intercal } +{\mathbf {G}}_{2,\varvec{\Psi }}\frac{\partial {{{\,\mathrm{\textsf {vec}}\,}}}{{\varvec{\Psi }}}}{\partial {\varvec{\theta }}^\intercal }\big ]\end{aligned}$$

18c $$\begin{aligned} \frac{\partial g_3({\varvec{\theta }}_{{{\gamma }}_3};{\mathbf {x}},{\mathbf {v}}_{{\mathcal {N}}})}{\partial {\varvec{\theta }}^\intercal }&=f({\mathbf {v}}_{{\mathcal {N}}}\mid do({\mathbf {x}}))\big [{\mathbf {G}}_{3,\varvec{\mu }},{\mathbf {G}}_{3,\varvec{\Sigma }}\big ] \begin{pmatrix} {\mathbf {1}}_{{\mathcal {N}}}^\intercal \frac{\partial {\mathbf {g}}_1 ({\varvec{\theta }}_{{{\varvec{\gamma }}}_1};{\mathbf {x}})}{\partial {\varvec{\theta }}^\intercal }\\ ({\mathbf {1}}_{{\mathcal {N}}}^\intercal \otimes {\mathbf {1}}_{{\mathcal {N}}}^\intercal ){\mathbf {D}}_n\frac{\partial {\mathbf {g}}_2({\varvec{\theta }}_{{{\varvec{\gamma }}}_2})}{\partial {\varvec{\theta }}^\intercal } \end{pmatrix}\end{aligned}$$

18d $$\begin{aligned} \frac{\partial g_4({\varvec{\theta }}_{{{\gamma }}_4};{\mathbf {x}},{\mathbf {y}}^{\text {low}}, {\mathbf {y}}^{\text {up}})}{\partial {\varvec{\theta }}^\intercal }&=\big [{\mathbf {G}}_{4,\mu },{\mathbf {G}}_{4,\sigma ^2} \big ] \left( \begin{array}{rl} \varvec{{{\imath }}}_{j}^\intercal &{} \frac{\partial {\mathbf {g}}_1({\varvec{\theta }}_{{{\varvec{\gamma }}}_1};{\mathbf {x}})}{\partial {\varvec{\theta }}^\intercal }\\ \varvec{{{\imath }}}_{(j-1)n+j}^\intercal &{}{\mathbf {D}}_n \frac{\partial {\mathbf {g}}_2({\varvec{\theta }}_{{{\varvec{\gamma }}}_2})}{\partial {\varvec{\theta }}^\intercal } \end{array}\right) \end{aligned}$$

Where the unit vector in the upper entry of the vector in Eq. (18d) is of dimension $(n \times 1)$ and the unit vector in the lower entry is of dimension $(n^2 \times 1)$. The matrices denoted by $\mathbf {G}$ and a subscript are defined as follows:

$$\begin{aligned} {\mathbf {G}}_{2,\mathbf{C}}&:=({\mathbf{I}}_{n^2}+{\mathbf {K}}_n) \big [(({\mathbf{I}}_n-{\mathbf{I}}_{{\mathcal {N}}}{\mathbf {C}})^{-1}{\mathbf{I}}_{{\mathcal {N}}} {\varvec{\Psi }}{\mathbf{I}}_{{\mathcal {N}}})\otimes {\mathbf{I}}_n\big ]\big [({\mathbf{I}}_n -{\mathbf{I}}_{{\mathcal {N}}}{\mathbf {C}})^{-\intercal } \otimes ( ({\mathbf{I}}_n-{\mathbf{I}}_{{\mathcal {N}}}{\mathbf {C}})^{-1}{\mathbf{I}}_{{\mathcal {N}}})\big ]\\ {\mathbf {G}}_{2,\varvec{\Psi }}&:=\big [({\mathbf{I}}_n-{\mathbf{I}}_{{\mathcal {N}}}{\mathbf {C}})^{-1} \otimes ({\mathbf{I}}_n-{\mathbf{I}}_{{\mathcal {N}}}{\mathbf {C}})^{-1}\big ] ({\mathbf{I}}_{{\mathcal {N}}}\otimes {\mathbf{I}}_{{\mathcal {N}}}) \\ {\mathbf {G}}_{3,\varvec{\mu }}&:=({\mathbf {v}}_{{\mathcal {N}}} -{\varvec{\mu }}_{{\mathcal {N}}})^\intercal {\varvec{\Sigma }}_{{\mathcal {N}}}^{-1}\\ {\mathbf {G}}_{3,\varvec{\Sigma }}&:=\frac{1}{2}\big (\big [({\mathbf {v}}_{{\mathcal {N}}} -{\varvec{\mu }}_{{\mathcal {N}}})^\intercal \otimes ({\mathbf {v}}_{{\mathcal {N}}} -{\varvec{\mu }}_{{\mathcal {N}}})^\intercal \big ]({\varvec{\Sigma }}_{{\mathcal {N}}}^{-1} \otimes {\varvec{\Sigma }}_{{\mathcal {N}}}^{-1})-{{{\,\mathrm{\textsf {vec}}\,}}} ({\varvec{\Sigma }}_{{\mathcal {N}}}^{-1})^\intercal \big )\\ {\mathbf {G}}_{4,\mu }&:= - \frac{1}{\sigma _y}\left[ \phi \left( \frac{y^{\text {up}} -\mu _y}{\sigma _y}\right) -\phi \left( \frac{y^{\text {low}}-\mu _y}{\sigma _y} \right) \right] \\ {\mathbf {G}}_{4,\sigma ^2}&:= - \frac{1}{2\sigma _y^{2}}\left[ \phi \left( \frac{y^{\text {up}}-\mu _y}{\sigma _y}\right) \left( \frac{y^{\text {up}} -\mu _y}{\sigma _y}\right) -\phi \left( \frac{y^{\text {low}} -\mu _y}{\sigma _y}\right) \left( \frac{y^{\text {low}}-\mu _y}{\sigma _y} \right) \right] \end{aligned}$$

Where ${\mathbf {L}}_n$, ${\mathbf {D}}_n$, and ${\mathbf {K}}_n$ denote the elimination matrix, duplication matrix, and commutation matrix for $n \times n$-matrices, respectively (Magnus & Neudecker, 1979, 1980). $\mu _y$ and $\sigma _y$ denote univariate inerventional moments.

### Proof

See Appendix. $$\square $$

Note that the Jacobian matrix for interventional probabilities stated in Eq. (18d) is given for a single outcome variable $Y=V_j$ (i.e., $|{\mathcal {Y}}|=K_y=1$). For simplicity of notation, the derivatives in Corollary 11 are taken with respect to the entire parameter vector ${\varvec{\theta }}$. Recall that a causal quantity is a function of the $s\times 1$ subvector ${\varvec{\theta }}_{\varvec{\gamma }}$. Consequently, the $r \times (q+p)$ Jacobian matrix $\frac{\partial {\mathbf {g}}({\varvec{\theta }}_{\varvec{\gamma }})}{\partial {\varvec{\theta }}^\intercal }$ will contain $(q+p-s)$ columns with zero entries that can be eliminated by pre-multiplication with an appropriate selection matrix.

These asymptotic results can be used for approximate causal inference based on finite samples, as will be illustrated in the following section.

## Illustration

We illustrate the method proposed in the previous paragraphs using simulated data. In this way, the data-generating process is known and we know with certainty that the model is correctly specified. For didactic purposes, we link the simulated data to a real-world example: The data are simulated according to a modified version of the model used in a study by Ito et al. (1998).^12^

**Fig. 2:**
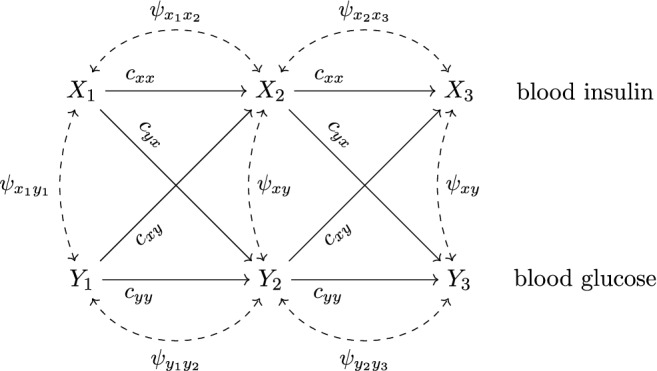
*Causal Graph (ADMG) in the Absence of Interventions*. Figure 2 displays the ADMG corresponding to the linear graph-based model. The dashed bidirected edge drawn between $X_1$ and $Y_1$ represents a correlation due to an unobserved common cause. Directed edges are labeled with the corresponding path coefficients that quantify direct causal effects. For example, the direct causal effect of $X_2$ on $Y_{3}$ is quantified as $c_{yx}$. Traditionally, disturbances (residuals, error terms), denoted by ${\varvec{\varepsilon }}$ in Eq. (19), are not explicitly drawn in an ADMG.

Our simulation mimics an observational study where $N=100$ persons are randomly drawn from a target population of homogeneous individuals and measured at three successive ($\Delta t=6$ min) occasions. Variables $X_1,X_2,X_3$ represent mean-centered blood insulin levels at three successive measurement occasions measured in micro international units per milliliter (mcIU/ml). Variables $Y_1,Y_2,Y_3$ represent mean-centered blood glucose levels measured in milligrams per deciliter (mg/dl). Mean-centered blood glucose levels below $-40 \ \text {mg/dl}$ or above $80 \ \text {mg/dl}$ indicate hypo- or hyperglycemia, respectively. Both hypo- and hyperglycemia should be avoided, yielding an acceptable range for blood glucose levels of $[y^{low},y^{up}]=[-40,80]$. The graph of the assumed linear graph-based models is depicted in Fig. 2.

Each directed edge corresponds to a direct causal effect and is quantified by a nonzero structural coefficient. We assume that direct causal effects are identical (stable) over time. For example, we assign the same parameter $c_{yx}$ to the directed edges $X_1 {\mathop {\rightarrow }\limits ^{c_{yx}}} Y_2$ and $X_2 {\mathop {\rightarrow }\limits ^{c_{yx}}} Y_3$ to indicate that we assume time-stable direct effects of $X_{t-1}$ on $Y_t$. The absence of a directed edge from, say, $X_1$ to $Y_3$ in the ADMG encodes the assumption that there is no direct effect of insulin levels at $t=1$ on glucose levels at $t=3$. In other words, we assume that $X_1$ only indirectly affects $Y_3$ via $X_2$ or via $Y_2$. Furthermore, we assume the absence of effect modification which justifies the use of the following system of linear structural equations:

19 $$\begin{aligned} \underbrace{\begin{pmatrix} X_1\\ Y_1\\ X_2\\ Y_2\\ X_3\\ Y_3 \end{pmatrix}}_{{\mathbf {V}}}=\underbrace{\begin{pmatrix} 0&{}0&{}0&{}0&{}0&{}0\\ 0&{}0&{}0&{}0&{}0&{}0\\ c_{xx}&{}c_{xy}&{}0&{}0&{}0&{}0\\ c_{yx}&{}c_{yy}&{}0&{}0&{}0&{}0\\ 0&{}0&{}c_{xx}&{}c_{xy}&{}0&{}0\\ 0&{}0&{}c_{yx}&{}c_{yy}&{}0&{}0 \end{pmatrix}}_{{\mathbf {C}}}\begin{pmatrix} X_1\\ Y_1\\ X_2\\ Y_2\\ X_3\\ Y_3 \end{pmatrix}+\underbrace{\begin{pmatrix} \varepsilon _{x1}\\ \varepsilon _{y1}\\ \varepsilon _{x2}\\ \varepsilon _{y2}\\ \varepsilon _{x3}\\ \varepsilon _{y3} \end{pmatrix}}_{{\varvec{\varepsilon }}} \end{aligned}$$

Each bidirected edge in the ADMG indicates the existence of an unobserved confounder. In linear graph-based models, unobserved confounders are formalized as covariances between error terms. The covariance matrix of the error terms implied by the graph is given by:

20 $$\begin{aligned} {\varvec{\Psi }}=\begin{pmatrix} \psi _{x_1x_1}&{}\psi _{x_1y_1}&{}\psi _{x_1x_2}&{}0&{}0&{}0\\ \psi _{x_1y_1}&{}\psi _{y_1y_1}&{}0&{}\psi _{y_1y_2}&{}0&{}0\\ \psi _{x_1x_2}&{}0&{}\psi _{xx}&{}\psi _{xy}&{}\psi _{x_2x_3}&{}0\\ 0&{}\psi _{y_1y_2}&{}\psi _{xy}&{}\psi _{yy}&{}0&{}\psi _{y_2y_3}\\ 0&{}0&{}\psi _{x_2x_3}&{}0&{}\psi _{xx}&{}\psi _{xy}\\ 0&{}0&{}0&{}\psi _{y_2y_3}&{}\psi _{xy}&{}\psi _{yy} \end{pmatrix} \end{aligned}$$

The entries $\psi _{x_1x_1}$, $\psi _{y_1y_1}$ and $\psi _{x_1y_1}$ describe the (co-)variances of the initial values of blood insulin and blood glucose. (Co-)Variances of error terms at time 2 and time 3 are assumed to be constant and are denoted as $\psi _{xx}$, $\psi _{yy}$, and $\psi _{xy}$. Serial correlations in the *X*-series (*Y*-series) are denoted by $\psi _{x_1x_2}$, $\psi _{x_2x_3}$ ($\psi _{y_1y_2}$, $\psi _{y_2y_3}$). The covariances $\mathrm {COV}(X_t,Y_t)$, $t=1,2,3$, encode the assumption that the contemporaneous relationship of blood insulin and blood glucose is confounded. The absence of a bidirected edge between $X_t$ and $Y_{t+1}$ encodes the assumption that there are no unobserved confounders that affect the lagged relationship of blood insulin and blood glucose.

Further, we assume that the error terms follow a multivariate normal distribution. Thus, the linear graph-based model is parametrized by the following vector of distinct, functionally unrelated and unknown parameters: ${\varvec{\theta }}^\intercal =({\varvec{\theta }}^\intercal _{{\mathcal {F}}},{\varvec{\theta }}^\intercal _{{\mathcal {P}}})$ with ${\varvec{\theta }}^\intercal _{{\mathcal {F}}}=(c_{xx},c_{xy},c_{yx},c_{yy})$ and ${\varvec{\theta }}^\intercal _{{\mathcal {P}}}=(\psi _{x_1x_1},\psi _{y_1y_1},\psi _{x_1y_1},\psi _{xx},\psi _{yy},\psi _{xy},\psi _{x_1x_2},\psi _{x_2x_3},\psi _{y_1y_2},\psi _{y_2y_3})$.

**Fig. 3:**
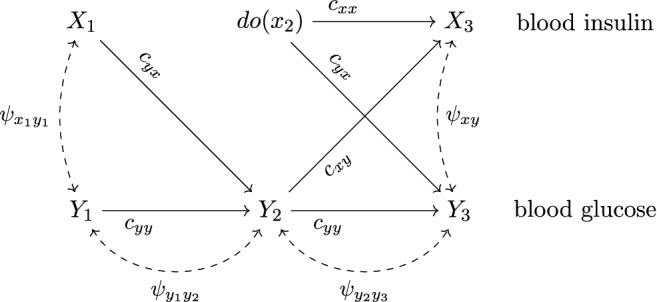
*Causal Graph (ADMG) Under the Intervention* $do(x_2)$. Figure 3 displays the ADMG of the graph-based model under the intervention $do(x_2)$. Edges that enter node $X_2$ (i.e., that have an arrowhead pointing at node $X_2$) are removed since the value of $X_2$ is now set by the experimenter via the intervention $do(x_2)$. The interventional value $x_2$ is neither determined by the values of the causal predecessors of $X_2$ nor by unobserved confounding variables. All other causal relations are unaffected by the intervention reflecting the assumption of modularity.

We are interested in the effect of an intervention on blood insulin at the second measurement occasion (i.e., $X_2$) on blood glucose levels at the third measurement occasion (i.e., $Y_3$). We set the interventional level of blood insulin to one standard deviation, namely $x_2=\sqrt{\text {V}(X_2)}=11.54$. The graph of the causal model under the intervention $do(x_2)$ is depicted in Fig. 3.

Based on the above description of the research situation and the hypothetical experiment, all terms in Definition 1 are uniquely determined and given by:

21 $$\begin{aligned} n&=6, \ {\mathcal {X}}=\{X_2\}, \ {\mathcal {Y}}=\{Y_3\}, \ K_x=K_y=1, \ {\mathcal {I}}=\{3\}, \ {\mathcal {N}}=\{1,2,4,5,6\}\nonumber \\ {\mathbf {x}}&=x_2=\sqrt{\text {V}(X_2)}, \ {\mathbf {1}}_{{\mathcal {I}}}=\begin{pmatrix} 0\\ 0\\ 1\\ 0\\ 0\\ 0 \end{pmatrix}, \ {\varvec{1}}_{{\mathcal {N}}}=\begin{pmatrix} 1&{}0&{}0&{}0&{}0\\ 0&{}1&{}0&{}0&{}0\\ 0&{}0&{}0&{}0&{}0\\ 0&{}0&{}1&{}0&{}0\\ 0&{}0&{}0&{}1&{}0\\ 0&{}0&{}0&{}0&{}1 \end{pmatrix}, \ {\mathbf{I}}_ {{\mathcal {N}}}=\begin{pmatrix} 1&{}0&{}0&{}0&{}0&{}0\\ 0&{}1&{}0&{}0&{}0&{}0\\ 0&{}0&{}0&{}0&{}0&{}0\\ 0&{}0&{}0&{}1&{}0&{}0\\ 0&{}0&{}0&{}0&{}1&{}0\\ 0&{}0&{}0&{}0&{}0&{}1 \end{pmatrix} \end{aligned}$$

The target quantity of causal inference in this example is the interventional distribution $P(Y_3 \mid do(x_2))$, which can be characterized, for example, by the following causal quantities:^13^

22a $$\begin{aligned} \gamma _{1}:=&{\text {E}}(Y_3 \mid do(x_2))=c_{yx}x_2 \end{aligned}$$

22b $$\begin{aligned} \gamma _{2}:=&\mathrm{V}(Y_3 \mid do(x_2))=c_{yx}^2 c_{yy}^2 \psi _{x_1x_1} + c_{yy}^4 \psi _{y_1y_1} + 2 c_{yx} c_{yy}^3 \psi _{x_1y_1}+ (1 + c_{yy}^2) \psi _{yy}\nonumber \\ +&2 c_{yy}^3 \psi _{y_1y_2}+ 2 c_{yy} \psi _{y_2y_3}\end{aligned}$$

22c $$\begin{aligned} \gamma _{3}:=&f(y_3 \mid do(x_2))=(2\pi )^{-\frac{1}{2}}(\mathrm{V}(Y_3 \mid do(x_2)))^{-\frac{1}{2}}\exp \left( -\frac{1}{2}\frac{(y_3-c_{yx} x_2)^2}{\mathrm{V}(Y_3 \mid do(x_2))}\right) \end{aligned}$$

22d $$\begin{aligned} \gamma _{4}:=&P(y^{low} \le Y_3 \le y^{up}\mid do(x_2))=\nonumber \\&\Phi \left( \frac{y^{up}-\mathrm{E}(Y_3 \mid do(x_2))}{\sqrt{\mathrm{V}(Y_3 \mid do(x_2))}} \right) - \Phi \left( \frac{y^{low}-\mathrm{E}(Y_3 \mid do(x_2))}{\sqrt{\mathrm{V}(Y_3 \mid do(x_2))}}\right) \end{aligned}$$

Where $\Phi$ denotes the cumulative distribution function (cdf) of the standard normal distribution. A central goal of a treatment at time 2 (i.e., $do(x_2)$) is to avoid hypo- or hyperglycemia at time 3. We therefore refer to the event $\{y^{low} \le Y_3 \le y^{up} \mid do(x_2)\}$ as *treatment success*. Using this terminology, the causal quantity $\gamma _{4}$ from Eq. (22d) is called the probability of treatment success.

The causal effect functions corresponding to these causal quantities are stated below and satisfy Property A.1:

23a $$\begin{aligned} \gamma _{1}&=g_1(\theta _{\gamma _1};x_2), \ \text {with} \ \theta _{\gamma _1}=c_{yx} \end{aligned}$$

23b $$\begin{aligned} \gamma _{2}&=g_2({\varvec{\theta }}_{\gamma _2}), \ \text {with} \ {\varvec{\theta }}_{\gamma _2}=(c_{yx},c_{yy},\psi _{x_1x_1},\psi _{x_1y_1}, \psi _{y_1y_1},\psi _{y_1y_2},\psi _{y_2y_3},\psi _{yy})^\intercal \end{aligned}$$

23c $$\begin{aligned} \gamma _{3}&=g_3({\varvec{\theta }}_{\gamma _3};x_2, y_3), \ \text {with} \ {\varvec{\theta }}_{\gamma _3}={\varvec{\theta }}_{\gamma _2} \end{aligned}$$

23d $$\begin{aligned} \gamma _{4}&=g_4({\varvec{\theta }}_{\gamma _4};x_2,y^{low},y^{up}), \ \text {with} \ {\varvec{\theta }}_{\gamma _4}={\varvec{\theta }}_{\gamma _2} \end{aligned}$$

**Fig. 4:**
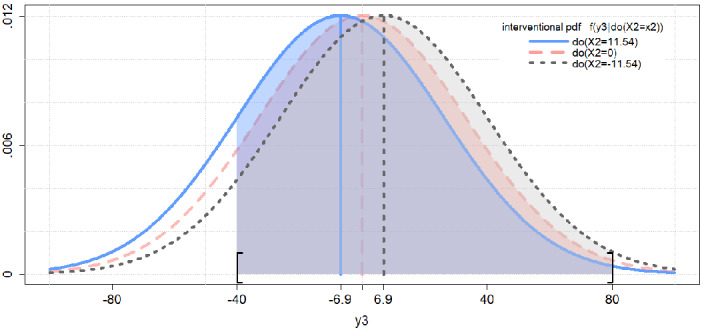
*Interventional Distributions for Three Distinct Treatment Levels*. Figure 4 displays several features of the interventional distribution for three distinct interventional levels $x_2=11.54$ (solid), $x_2'=0$ (dashed), and $x_2''=-11.54$ (dotted). The pdfs of the interventional distributions are represented by the bell-shaped curves. The interventional means are represented by vertical line segments. The interventional variances correspond to the width of the bell-shaped curves and are equal across the different interventional levels. The probabilities of treatment success are represented by the shaded areas below the curves in the interval $[-40,80]$.

Figure 4 displays the pdfs of interventional distributions that result from three distinct (hypothetical) experiments where different interventional levels are chosen, namely $-11.54$, 0, and 11.54. Note that the interventional mean $\gamma _{1}=g_1(\theta _{\gamma _1};x_2)$ is functionally dependent on the interventional level $x_2$ (see also Eq. [22a]). Thus, the location of the interventional distributions in Fig. 4 depends on the interventional level $x_2$. By contrast, the interventional variance $\gamma _{2}=g_2({\varvec{\theta }}_{\gamma _2})$ is functionally independent of $x_2$ (see also Eq. [22b]). Consequently, the scale of the interventional distributions in Fig. 4 is the same for all interventional levels.

Equations 23(a-d) display the causal effect functions corresponding to the causal quantities $\gamma _{1},\dots ,\gamma _{4}$. Definition 6 states that the parametrized causal quantities $\gamma _{1},\dots ,\gamma _{4}$ are identified if the corresponding parameters $\theta _{\gamma _1},{\varvec{\theta }}_{\gamma _2},{\varvec{\theta }}_{\gamma _3}, \mathrm{and}\, {\varvec{\theta }}_{\gamma _4}$ can be uniquely computed from the joint distribution of the observed variables. We show in the Appendix that the values of the entire parameter vector ${\varvec{\theta }}$ can be uniquely computed from the joint distribution of the observed variables. In fact, the values of ${\varvec{\theta }}$ can be uniquely computed from the covariance matrix of the observed variables.^14^

The joint distribution of the observed variables is given by $\{P({\mathbf {v}},{\varvec{\theta }})\mid {\varvec{\theta }} \in {\varvec{\Theta }}\}$, where *P* is the family of 6-dimensional multivariate normal distributions. We estimated all parameters simultaneously by minimizing the maximum likelihood discrepancy function of the model implied covariance matrix and the sample covariance matrix. The ML-estimator $\widehat{{\varvec{\theta }}}^{ML}$ is consistent, asymptotically efficient, and asymptotically normally distributed (Bollen, 1989) and therefore satisfies Property A.2. Additionally, the asymptotic covariance matrix of the ML-estimator is known (e.g., see Bollen, 1989) and consistent estimates thereof are implemented in many statistical software packages (e.g., in the R package lavaan; Rosseel, 2012). The corresponding estimation results for ${\varvec{\theta }}$ are displayed in Table 1.

Since Property A.1 and Property A.2 are satisfied, the asymptotic properties of the estimators $\widehat{{\gamma }}_1,\widehat{{\gamma }}_2,\widehat{{\gamma }}_3$ and $\widehat{{\gamma }}_4$ can be established via Theorem 8. The Jacobian matrices of the causal effect functions in Eq. (23) can be calculated according to Corollary 11. Estimates of the causal quantities are reported in Table 2 together with estimates of the asymptotic standard errors and approximate *z*-values.

**Table 1:** *Parameters in the Linear Graph-Based Model.*

	Structural coefficients									
	$c_{xx}$	$c_{xy}$	$c_{yx}$	$c_{yy}$						
Population	0.05	0.4	$-0.6$	1.2						
Estimate	0.08	0.39	$-0.52$	1.18						
Est. ASE	0.08	0.03	0.09	0.04						
*z*-value	1.00	13.00	$-5.78$	29.50						

	Variance-covariance parameters									
	$\psi _{x_1x_1}$	$\psi _{y_1y_1}$	$\psi _{x_1y_1}$	$\psi _{xx}$	$\psi _{yy}$	$\psi _{xy}$	$\psi _{x_1x_2}$	$\psi _{x_2x_3}$	$\psi _{y_1y_2}$	$\psi _{y_2y_3}$
Population	131.76	632.94	254.12	20	40	3	15	2	35	10
Estimate	126.32	601.85	241.19	22.15	35.88	1.71	16.57	2.31	28.96	9.03
Est. ASE	17.02	83.23	35.83	2.58	3.93	1.93	2.71	1.78	7.07	3.29
*z*-value	7.42	7.23	6.73	8.59	9.13	0.89	6.11	1.30	4.10	2.74

The estimation results $\widehat{{\varvec{\theta }}}^{ML}$ for the model parameters ${\varvec{\theta }}$ (using a covariance-based maximum likelihood estimator with $N=100$) are displayed together with the true population values used for data simulation. The *z*-values are reported for the null hypothesis of a population quantity equal to zero. Structural coefficients are displayed in the upper part, and the variance–covariance parameters are displayed in the lower part. ASE = asymptotic standard error.

**Table 2:** *Causal Quantities in the Linear Graph-Based Model.*

	${\widehat{\gamma }}_{1}$	${\widehat{\gamma }}_{2}$	${\widehat{\gamma }}_{3}^{\dagger }$	${\widehat{\gamma }}_{4}^{\dagger }$
Population	$-0.6000x_2$	1096.3855	0.0120	0.8368
Estimate	$-0.5217x_2$	1007.2180	0.0123	0.8545
Est. ASE	$0.0909x_2$	146.7012	0.0009	0.0007
*z*-value	$-5.7393$	6.8658	13.6667	1220.71

The estimation results for the causal quantities $\gamma _{1}$, $\gamma _{2}$, $\gamma _{3}$, and $\gamma _{4}$ are displayed together with the population values used for data simulation. The *z*-values are reported for the null hypothesis of a population quantity equal to zero. $^\dagger$ The estimates ${\widehat{\gamma }}_{3}$ and ${\widehat{\gamma }}_{4}$ depend on $x_2$, $y_3$, $y_3^{low}$, or $y_3^{up}$ in a nonlinear way. The displayed quantities are calculated for $x_2=11.54$, $y_3=0$, $y_3^{low}=-40$ and $y_3^{up}=80$. ASE = asymptotic standard error.

**Fig. 5:**
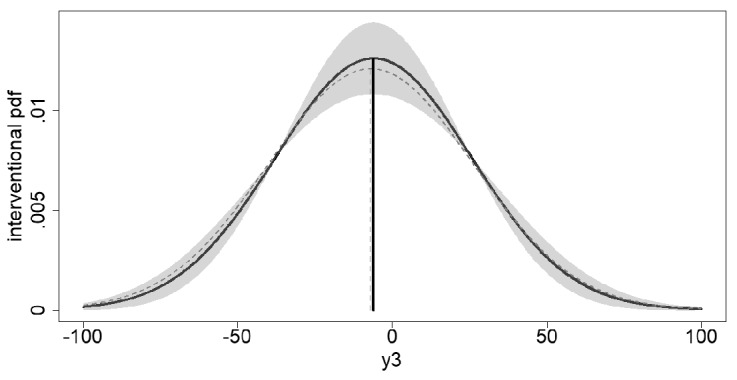
*Estimate of the Probability Density Function of the Interventional Distribution*. Figure 5 displays the estimated interventional pdf ${\widehat{f}}(y_3 \mid do(x_2=11.54))$ (black solid line) with pointwise $95\%$ confidence intervals, that is, $\pm 1.96\cdot {\widehat{\mathrm{ASE}}}[{\widehat{f}}(y_3 \mid do(x_2=11.54))]$ (gray shaded area). The true population interventional pdf $f(y_3 \mid do(x_2=11.54))$ is displayed by the gray dashed line.

From Theorem 8, we know that $\widehat{\gamma }_3=g_3(\widehat{{\varvec{\theta }}}_{\gamma _3};x_2, y_3)=\widehat{f}(y_3 \mid do(x_2))\xrightarrow { \ p \ } f(y_3 \mid do(x_2))$ holds pointwise for any $(y_3,x_2) \in {\mathbb {R}}^2$. Figure 5 displays the estimated interventional pdf together with its population counterpart as well as pointwise asymptotic confidence intervals for the fixed interventional level $x_2=11.54$ over the range $y_3\in [-100,100]$.

Figure 5 shows that a sample size of $N=100$ yields very precise estimates of the interventional pdf over the whole range of values $y_3\in [-100,100]$, which is a consequence of the rate of convergence $N^{-\frac{1}{2}}$ established in Theorem 8.

**Fig. 6:**
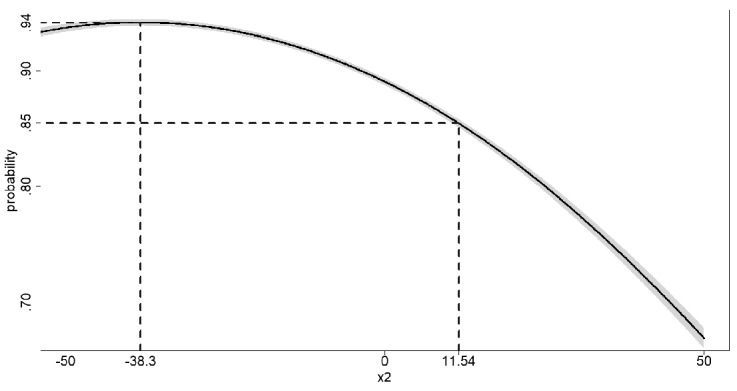
*Estimated Probability of Treatment Success*. Figure 6 displays the estimated probability of treatment success (i.e., ${\widehat{\gamma }}_4={\widehat{P}}(-40 \le Y_3 \le 80\mid do(x_2))$; black solid line) as a function of the interventional level $x_2$. The pointwise confidence intervals $\pm 1.96\cdot {\widehat{\mathrm{ASE}}}[{\widehat{P}}(-40 \le Y_3 \le 80\mid do(x_2))]$ are displayed by the (very narrow) gray shaded area around the solid black line (see electronic version for high resolution). The vertical dashed lines are drawn at the interventional levels $x_2=11.54$ and $x_2=-38.3$. The horizontal dashed lines correspond to the probabilities of treatment success for the treatments $do(X_2=11.54)$ and $do(X_2=-33.3)$.

Figure 6 displays the estimated probability that the blood glucose level falls into the acceptable range (i.e., hypo- and hyperglycemia are avoided) at $t=3$, given an intervention $do(x_2)$ on blood insulin at $t=2$, as a function of the interventional level $x_2$. Just like in the case of the interventional pdf, Fig. 6 shows that a sample size of $N=100$ yields very precise estimates of interventional probabilities over the whole range of values $x_2\in [-50,50]$. Given the intervention $do(x_2=11.54)$, the probability of treatment success (i.e, blood glucose level within the acceptable range at $t=3$) equals .85, as depicted in Fig. 6. Since the curve in Fig. 6 displays a unique (local and global) maximum, the interventional level can be chosen such that the probability of treatment success is maximized. The maximal probability of treatment success is equal to .94 and can be obtained by administering intervention $do(x_2^*=-38.3)$. Note that the curve is relatively flat around its maximum, meaning that slight deviations from the optimal treatment level will result in a small decrease in the probability of treatment success.

### Interventional Distribution vs. Conditional Distribution

**Fig. 7:**
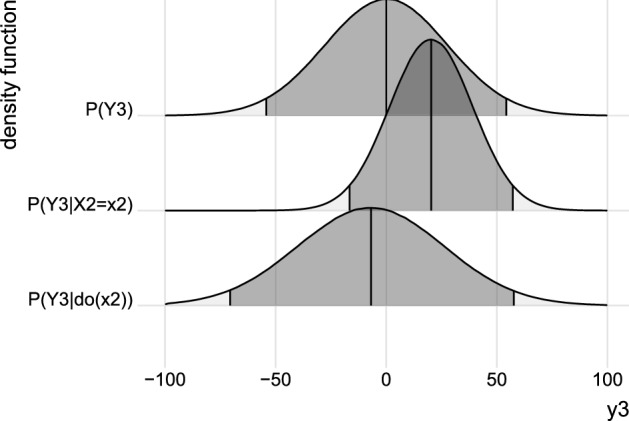
*Marginal, Conditional, and Interventional Distribution*. The panels depict (i) the pdf of the unconditional distribution $P(Y_3)$ (top panel), (ii) the conditional distribution $P(Y_3 \mid X_2=x_2)$ (middle panel), and (iii) the interventional distribution $P(Y_3 \mid do(x_2))$ (bottom panel). In (ii) the level $x_2=11.54 \ \text {mg/dl}$ was passively measured whereas in (iii) the intervention $do(X_2=11.54)$ was performed. The central vertical black solid lines are drawn at the mean and shaded areas cover $95\%$ of the probability mass.

To illustrate the conceptual differences between the interventional and conditional distribution, we use the numeric population values from the first row of Table 1 and Table 2, respectively. The interventional distribution is given by $P(Y_3 \mid do(x_2))={{N}}_{1}(-0.6x_2 \, , \, 1096.39)$ and it differs from both the conditional distribution, $P(Y_3 \mid X_2=x_2)={{N}}_{1}(1.76x_2 \, , \, 353.99)$, and the unconditional distribution, $P(Y_3)={{N}}_{1}(0 \, , \,766.91)$, as depicted in Fig. 7.^15^

The unconditional distribution (upper panel) corresponds to a situation where no prior observation is available and no intervention is performed. Note that the conditional distribution (middle panel) is shifted to the right (for $X_2=11.54$), whereas the interventional distribution (bottom panel) is shifted to the left for $do(X_2=11.54)$, as displayed in Fig. 7. The differences displayed between $P(Y_3 \mid X_2=11.54)$ and $P(Y_3 \mid do(X_2=11.54))$ reflect the fundamental difference between the mode of *seeing*, namely passive observation, and the mode of *doing*, namely active intervention (Pearl, 2009).

On the one hand, observing a blood insulin level of $X_2=11.54$ at the second measurement occasion leads to an expected value of $20.31 \ \text {mg/dl}$ for blood glucose at the third measurement occasion (i.e., $\mathrm{E}(Y_3 \mid X_2=11.54)=1.76\cdot 11.54=20.31$). Using the conditional variance $\mathrm{V}(Y_3 \mid X_2=11.54)=353.99$ to compute a $95\%$ forecast interval yields $P(Y_3 \in [-16.56,57.19] \mid X_2=11.54)=.95$, as indicated by the shaded area under the curve in the middle panel of Fig. 7.

On the other hand, setting the level of blood insulin to $do(X_2=11.54)$ at the second occasion by an active intervention leads to an expected value of $-6.92 \ \text {mg/dl}$ for blood glucose at the third measurement occasion (i.e., $\mathrm{E}(Y_3 \mid do(x_2=11.54))=-0.60\cdot 11.54=-6.92$). Using the interventional variance $\mathrm{V}(Y_3 \mid do(11.54))=1096.39$ to compute a $95\%$ forecast interval yields $P(Y_3 \in [-71.82,57.97] \mid do(x_2=11.54))=.95$, as indicated by the shaded area under the curve in the bottom panel of Fig. 7.

Based on both the conditional and interventional distribution, valid statements about values of blood glucose can be made. A patient who measures a high level of insulin at time 2 in the absence of an intervention (e.g., self-measured monitoring of blood insulin; mode of seeing) will predict a high level of blood glucose at time 3 based on the conditional distribution. A physician who actively administers a high dose of insulin at time 2 (e.g., via an insulin injection; mode of doing) will forecast a low value of blood glucose at time 3 based on the interventional distribution.

Incorrect conclusions arise if the conditional distribution is used to forecast effects of interventions, or the other way around, the interventional distribution is used to predict future values of blood glucose in the absence of interventions. For example, a physician who correctly uses the interventional distribution to choose the optimal treatment level would administer $do(X_2=-38.3)$, resulting in a $94\%$ probability of treatment success (see Fig. 6). A physician who erroneously uses the conditional distribution to specify the optimal treatment level would administer $do(X_2=11.4)$. Such a non-optimal intervention would result in a $85\%$ probability of treatment success. Thus, an incorrect decision results in an absolute decrease of $9\%$ in the probability of treatment success (Gische et al., 2021).

## Discussion

Graph-based causal models combine a priori assumptions about the causal structure of the data-generating mechanism (e.g., encoded in a ADMG) and observational data to make inference about the effects of (hypothetical) interventions. Causal quantities are defined via the *do*-operator and may comprise any feature of the interventional distribution (e.g., the mean vector, the covariance matrix, the pdf). This flexibility allows researchers to analyze effects of interventions beyond changes in the mean level. Causal effect functions map the parameters of the model implied joint distribution of observed variables onto causal quantities and therefore enable analyzing causal quantities using tools from the literature on traditional SEM. We propose an estimator for causal quantities and show that it is consistent and converges at a rate of $N^{-\frac{1}{2}}$. In case of maximum likelihood estimation, the proposed estimator is asymptotically efficient.

In the remainder of the paper, we discuss several situations in which linear graph-based models are misspecified and how the proposed procedure can be extended to be applicable in such situations.

### Causal Structure, Modularity, and Conditional Interventions

A researcher’s beliefs about the causal structure are encoded in the graph. Based on the concept of *d*-separation, every ADMG implies a set of (conditional) independence relations between observable variables that can be tested parametrically (Chen, Tian, & Pearl, 2014; Shipley, 2003; Thoemmes, Rosseel, & Textor, 2018) or nonparametrically (Richardson, 2003; Tian & Pearl, 2002b). One drawback of these tests is that they only distinguish between equivalence classes of ADMGs and do not evaluate the validity of a single graph.

One way of dealing with this situation is to further analyze the equivalence class to which a specified model belongs (Richardson & Spirtes, 2002). Some authors have proposed methods to draw causal conclusions based on common features of an entire equivalence class instead of using a single model (Hauser & Bühlmann, 2015; Maathuis, Kalisch, & Bühlmann, 2009; Perkovic, 2020; Zhang, 2008). However, equivalence classes can be large and its members might not overlap with respect to the causal effects of interest (He & Jia, 2015).

Another approach discussed in the literature is to complement the available observational data with *experimental* data. If these experiments are optimally chosen, the size of an equivalence class can be substantially reduced (Eberhardt, Glymour, & Scheines, 2005; Hyttinen, Eberhardt, & Hoyer, 2013). The idea of combining observational data and experimental data is theoretically appealing for many reasons, and it has stimulated the development of a variety of techniques (He & Geng, 2008; Peters, Bühlmann, & Meinshausen, 2016; Sontakke, Mehrjou, Itti, & Schölkopf, 2020). Most importantly, the combination of observational and interventional data allows differentiating causal models that cannot be distinguished solely based on observational data.

Furthermore, the availability of experimental evidence enables (partly) testing further causal assumptions such as the assumption of modularity, which cannot be tested solely based on observational data. While modularity seems rather plausible if the mechanisms correspond to natural laws (e.g., chemical or biological processes, genetic laws, laws of physics), it needs additional reflection if the mechanisms describe human behavior. For example, humans might respond to an intervention by adjusting behavioral mechanisms different from the one that is intervened on. The proposed method can readily be adjusted to capture such violations of the modularity assumption if an intervention changes other mechanisms in a known way. However, if the ways in which humans adjust their behavior in response to an intervention are unknown, they need to be learned. Well-designed experiments may be particularly useful for this purpose.

Throughout the manuscript, we focus on specific *do*-type interventions that assign fixed values to the interventional variables according to an exogenous rule. However, in practical applications interventional values are often chosen conditionally on the values of other observed variables. In our illustrative example, the interventional insulin level at $t=2$ might be chosen in response to the glucose level observed at $t=1$. Such situations are discussed in the literature on conditional interventions (Pearl, 2009) and dynamic treatment plans (Pearl & Robins, 1995; Robins, Hernán, & Brumback, 2000). In principle, the proposed method can be extended to evaluate conditional interventions and effects of dynamic treatment plans. However, the derivation of the closed-form representations of parametrized causal quantities and the corresponding causal effect functions in these settings require further research.

Finally, consequences of specific violations of non-testable causal assumptions can be gauged via sensitivity analyses and robustness checks (Ding & VanderWeele, 2016; Dorie, Harada, Carnegie, & Hill, 2016; Franks, D’Amour, & Feller, 2020; Rosenbaum, 2002).

### Effect Modification and Heterogeneity

In this article, we have focused on situations in which direct causal effects are constant across value combinations of observed variables and error terms. In such situations, the use of linear models is justified. Statistical tests for linearity of the functional relations exist for both nested and non-nested models (Amemiya, 1985; Lee, 2007; Schumacker & Marcoulides, 1998). If these tests provide evidence against linearity, the assumption of constant direct effects is likely to be violated.

Theoretical considerations often suggest the existence of so-called effect modifiers (moderators), which can be modeled in parametrized graph-based models via nonlinear structural equations (Amemiya, 1985; Klein & Muthén, 2007). However, a closed-form representation of the entire interventional distribution in case of nonlinear structural relations cannot be derived via a direct application of the method proposed in this paper. The extent to which the proposed parametric method can be generalized to capture common types of nonlinearity (e.g., simple product terms that capture certain types of effect modification) is a focus of ongoing research. Preliminary results suggest that parametrized closed-form expressions of certain features of the interventional distribution (e.g., its moments) can be obtained (Kan, 2008; Wall & Amemiya, 2003), which in turns enables analyzing ATEs and other causal quantities.

Furthermore, we assumed that direct causal effects quantified by structural coefficients are equal across individuals in the population. However, (unobserved) heterogeneity in mean levels or direct effects might be present in many applied situations. A common procedure to capture specific types of unobserved heterogeneity is to include random intercepts or random coefficients in panel data models (Hamaker, Kuiper, & Grasman, 2015; Usami, Murayama, & Hamaker, 2019; Zyphur et al. 2019). Gische et al. (2021) apply the method proposed in this paper to linear cross-lagged panel models with additive person-specific random intercepts and show how *absolute values* of optimal treatment levels differ across individuals.

Even though additive random intercepts capture unobserved person-specific differences in the mean levels of the variables, these models still imply constant effects of changes in treatment level across persons. The latter implication might be overly restrictive in many applied situations in which treatment effects vary across individuals (e.g., different patients respond differently to variations in treatment level). An extension of the proposed methods to more complex dynamic panel data models (e.g., models including random slopes) requires further research. Several alternative approaches to model effect heterogeneity have been proposed for example within the social and behavioral sciences (Xie, Brand, & Jann, 2012), economics (Athey & Imbens, 2016), the political sciences (Imai & Ratkovic, 2013), and the computer sciences (Nie & Wager, 2020; Wager & Athey, 2018).

### Measurement Error and Non-Normality

We assumed that variables are observed without measurement error. The proposed method can be extended to define, identify, and estimate causal effects among latent variables. In other words, measurement errors and measurement models can be included. The model implied joint distribution of observed variables in latent variable SEM is known (Bollen, 1989), and the derivation of the parametric expressions for causal quantities and causal effect functions in such models is subject to ongoing research.

However, measurement models for latent variables often can only mitigate measurement error issues (unless the true measurement model is known and everything is correctly specified). Furthermore, the degree to which interventions on certain types of latent constructs is feasible in practice needs further discussion (e.g., see Bollen, 2002; Borsboom, Mellenbergh, & van Heerden, 2003; van Bork, Rhemtulla, Sijtsma, & Borsboom, 2020).

Some population results derived in this paper rely on multivariate normally distributed error terms (e.g., Result 3), while others do not (e.g., the moments of the interventional distribution in Eqs. (6a) and (6b) or Theorem 8). For the former results, a systematic analytic inquiry of the consequences of incorrectly assuming multivariate normal error terms requires specific knowledge about the type of misspecification. If such knowledge is not available, one could attempt to assess the sensitivity of, for example, the interventional pdf, to misspecifications in the error term distribution via simulation studies.

Some estimation results derived in this paper rely on a known parametric distributional family of the error terms (e.g., Theorem 9 requires maximum-likelihood estimation), while others do not (e.g., Theorem 8 ensures consistency of the estimators of causal quantities for a broad class of estimators including ADF or WLS estimation of ${\varvec{\theta }}$). Thus, inference about the interventional moments can be conducted in the absence of parametric assumptions on the error term distribution. Furthermore, it has been shown that ML-estimators in linear SEM are robust to certain types of distributional misspecification but sensitive to others (West, Finch, & Curran, 1995) and robust estimators have been developed for several types of distributional misspecifications (Satorra & Bentler, 1994; Yuan & Bentler, 1998).

### Conclusion

Causal graphs (e.g., ADMGs) allow researchers to express their causal beliefs in a transparent way and provide a sound basis for the definition of causal effects using the *do*-operator. Causal effect functions enable analyzing causal quantities in parametrized models. They are a flexible tool that allow researchers to model causal effects beyond the mean and covariance structure and can thus be applied in a large variety of research situations. Consistent and asymptotically efficient estimators of parametric causal quantities are provided that yield precise estimates based on sample sizes commonly available in the social and behavioral sciences.

### Supplementary Information

Below is the link to the electronic supplementary material.

Supplementary file 1 (pdf 644 KB)

## Appendix

### Proof of Lemma 2

*Proof of* $\text {rank}({\mathbf {T}}_1)=n-K_x$: The matrix $({\mathbf{I}}_n-{\mathbf{I}}_ {{\mathcal {N}}}{\mathbf {C}})^{-1}$ is lower triangular with ones on the diagonal and thus has full rank *n* (Lütkepohl, 1997, result 9.14.1(4)(c), p. 165). By construction $\mathbf {{\mathbf{I}}}_{{\mathcal {N}}}$ is a diagonal matrix where $K_x$ diagonal elements are equal to zero which implies $\mathrm {rank}(\mathbf {{\mathbf{I}}}_{{\mathcal {N}}})=n-K_x$ (Lütkepohl 1997, result 9.4(3)(a), p. 120). Thus, $\text {rank}({\mathbf {T}}_1)=\text {rank}(({\mathbf{I}}_n-{\mathbf{I}}_ {{\mathcal {N}}}{\mathbf {C}})^{-1}\mathbf {{\mathbf{I}}}_{{\mathcal {N}}})=n-K_x$, where the last equality sign follows from result 4.3.1(9) in Lütkepohl (1997).

*Proof of* $\text {rank}({\mathbf {T}}_2)=n-K_x$: $({\mathbf{I}}_n-{\mathbf{I}}_ {{\mathcal {N}}}{\mathbf {C}})^{-1}$ is a lower triangular matrix of full rank *n* that has ones on the diagonal. Postmultiplying $({\mathbf{I}}_n-{\mathbf{I}}_ {{\mathcal {N}}}{\mathbf {C}})^{-1}$ with $\mathbf {{\mathbf{I}}}_{{\mathcal {N}}}$ sets all columns with index $i \in {\mathcal {I}}$ to zero. Or more formally, $[{\mathbf {t}}_{1}]_{\bullet i}={\mathbf {0}}_{n \times 1}, \, i \in {\mathcal {I}}$, where $[{\mathbf {t}}_{1}]_{\bullet i}$ denotes the *i*-th column of the matrix ${\mathbf {T}}_1$. Similarly, $[{\mathbf {t}}_{1}]_{i \bullet }$ denotes the *i*-th row of the matrix ${\mathbf {T}}_1$. Thus, all diagonal elements $t_{ii}$ with $\ i \in {\mathcal {I}}$ are equal to zero. Premultiplying ${\mathbf {T}}_1$ with ${\mathbf {1}}_{{\mathcal {N}}}^{\intercal }$ deletes all rows $[{\mathbf {t}}_{1}]_{i\bullet }$ that have an index $i \in {\mathcal {I}}$. The deleted rows are exactly those rows that have $t_{ii}=0$ as diagonal elements. The matrix ${\mathbf {T}}_2={\mathbf {1}}_{{\mathcal {N}}}^{\intercal }{\mathbf {T}}_1$ contains only those rows of ${\mathbf {T}}_1$ that have a non-interventional index, that is, rows that have diagonal elements $t_{ii}$ equal to 1. The resulting structure of ${\mathbf {T}}_2$ is illustrated below:

$$\begin{aligned} {\mathbf {T}}_{2}=\begin{array}{cccccccccccc|l} [{\mathbf {t}}_{2}]_{\bullet 1} &{}&{}\dots &{}&{} [{\mathbf {t}}_{2}]_{\bullet _{j_2}} &{}&{}\dots &{}&{}&{} [{\mathbf {t}}_{2}]_{\bullet _{j_3}}&{} \dots &{} [{\mathbf {t}}_{2}]_{\bullet n}\\ \hline 1 &{} 0 &{} \dots &{} 0 &{} 0 &{} 0 &{} \dots &{} 0 &{} 0 &{} 0 &{} \dots &{} 0 &{} [{\mathbf {t}}_{2}]_{1 \bullet }\\ * &{} * &{} \dots &{} * &{} 1 &{} 0 &{} \dots &{} 0 &{} 0 &{} 0 &{} \dots &{} 0 &{} [{\mathbf {t}}_{2}]_{2 \bullet }\\ *&{} * &{} \dots &{} * &{} * &{} * &{} \dots &{} * &{} 1 &{} 0 &{} \dots &{} 0 &{} [{\mathbf {t}}_{2}]_{3 \bullet }\\ \vdots &{} &{} &{} &{} &{} &{} &{} &{} &{} &{} &{} \vdots &{} \vdots \\ * &{} * &{} \dots &{} * &{} * &{} * &{} \dots &{} * &{} * &{} * &{} \dots &{} 1 &{} [{\mathbf {t}}_{2}]_{(n-K_x)\bullet } \end{array} \end{aligned}$$

The ordered set of non-interventional indexes is given by ${\mathcal {N}}:=\{1,2,...,n\}\setminus {\mathcal {I}}=\{j_1,j_2,...,j_{n-K_x}\}$. For clarity of display (and without loss of generality), we assume $j_1=1$ and $j_{n-K_x}=n$, that is, variables $V_1$ and $V_n$ are *not* subject to intervention. Due to the step structure of the matrix ${\mathbf {T}}_{2}$ with the rightmost nonzero element of each row equal to one, the matrix ${\mathbf {T}}_{2}$ has full row rank, that is, $\text {rank}({\mathbf {T}}_2)=n-K_x$. $\square$

### Sketch of proof of local identification of example model

Due to space restrictions and the necessity to state high-dimensional vectors and matrices explicitly, a detailed and fully reproducible version of the proof is given in the online supplementary material.

Let ${\mathbf {V}}={\mathbf {C}}{\mathbf {V}}+{\varvec{\varepsilon }}$ be a linear graph-based model as defined in Eq. (2), where $n=6$, and ${\mathbf {C}}$ and ${\varvec{\Psi }}$ are given in Eq. (19) and (20), respectively. Plugging in these quantities into Eq. (3.3.6) from Bekker et al. (1994) yields:

A.1 $$\begin{aligned} \tilde{{\mathbf {J}}}=\begin{pmatrix} {\mathbf {R}}_{{\varvec{\Psi }}}(\mathbf {{\mathbf{I}}}_{36}+{\mathbf {K}}_6)(\mathbf {{\mathbf{I}}}_6 \otimes {\varvec{\Psi }})\\ {\mathbf {R}}_{\mathbf {C}}(\mathbf {{\mathbf{I}}}_6 \otimes (\mathbf {{\mathbf{I}}}-{\mathbf {C}})^\intercal ) \end{pmatrix} \quad , \quad (43 \times 36) \end{aligned}$$

The $(32 \times 36)$ matrix ${\mathbf {R}}_{{\varvec{\Psi }}}$ and the $(11 \times 36)$ matrix ${\mathbf {R}}_\mathbf{{C}}$ encode the zero restrictions and equality constraints imposed on the covariance matrix and the matrix of structural coefficients in Eqs. (20) and (19), respectively. The matrix ${\mathbf {K}}_6$ denotes the commutation matrix for $n\times n$ matrices (Magnus & Neudecker, 1979). Theorem 3.3.1 in Bekker et al. (1994) states that under certain regularity conditions the parameter vector ${\varvec{\theta }}$ is locally identified, if and only if, the Jacobian matrix $\tilde{{\mathbf {J}}}$ has full column rank. We show that $\mathrm {rank}(\tilde{{\mathbf {J}}})=36$. The exact form of the restriction matrices ${\mathbf {R}}_{{\varvec{\Psi }}}$ and ${\mathbf {R}}_\mathbf{{C}}$, and the Mathematica (Wolfram Research Inc., 2018) code used to evaluate the rank of $\tilde{{\mathbf {J}}}$ are provided in the online supplementary material. $\square$

*Properties Required for* Theorem 8:

### Property A.1

(properties of causal effect functions ${\mathbf {g}}$) Let ${\varvec{\gamma }}$ be a causal quantity and ${\mathbf {g}}({\varvec{\theta }}_{\varvec{\gamma }})$ the corresponding causal effect function. Let ${\mathbf {g}}({\varvec{\theta }}_{\varvec{\gamma }})$ be continuously differentiable with respect to ${\varvec{\theta }}_{\varvec{\gamma }}$ in a neighborhood around the true population parameter value ${\varvec{\theta }}^*_{\varvec{\gamma }}\in {\varvec{\Theta }}_{\varvec{\gamma }}$. The $r \times s$ matrix of partial derivatives is non-singular and denoted by $\frac{\partial {\mathbf {g}}({\varvec{\theta }}_{\varvec{\gamma }})}{\partial {\varvec{\theta }}_{\varvec{\gamma }}^\intercal }$. If the causal effect function contains auxiliary variables, say ${\mathbf {g}}({\varvec{\theta }}_{\varvec{\gamma }};{\mathbf {x}},{\mathbf {v}}_{{\mathcal {N}}})$, then non-singularity of the matrix of partial derivatives is supposed to hold for any fixed value combination $({\mathbf {x}},{\mathbf {v}}_{{\mathcal {N}}})\in {\mathbb {R}}^{K_x}\times {\mathbb {R}}^{n-K_x}$.^16^

### Property A.2

(statistical properties of $\widehat{{\varvec{\theta }}}_{\varvec{\gamma }}$) Let $\widehat{{\varvec{\theta }}}_{\varvec{\gamma }}$ be an estimator of ${\varvec{\theta }}_{\varvec{\gamma }}$ with:

A.2a $$\begin{aligned} \widehat{{\varvec{\theta }}}_{\varvec{\gamma }}&\xrightarrow { \ p \ }{\varvec{\theta }}^*_{\varvec{\gamma }} \end{aligned}$$

A.2b $$\begin{aligned} \sqrt{N}\left( \widehat{{{\varvec{\theta }}}}_{{\varvec{\gamma }}}-{{\varvec{\theta }}}^*_{{{\varvec{\gamma }}}} \right)&\xrightarrow { \ d \ } { {N}}_{s} \big (\,{\mathbf {0}}_s\, , \, \mathrm {AV}(\sqrt{N}\widehat{{{\varvec{\theta }}}}_{{\varvec{\gamma }}})\,\big ) \end{aligned}$$

Where ${\varvec{\theta }}^*$ denotes the true population value and $\xrightarrow { \ p \ }$ ($\xrightarrow { \ d \ }$) refers to convergence in probability (distribution) as the sample size *N* tends to infinity. The covariance matrix of the limiting distribution is denoted as $\mathrm{AV}(\sqrt{N}\widehat{{\varvec{\theta }}}_{\varvec{\gamma }})$ and is assumed to be finite.^17^

### Proof of Corollary 11

We follow the definition of a matrix differential and a matrix derivative in Magnus and Neudecker (1999). To complete the proof, we make extensive use of results (a) from matrix differential calculus (Abadir & Magnus, 2005; Magnus & Neudecker, 1999) and (b) regarding the ${{\,\mathrm{\textsf {vec}}\,}}$-operator and Kronecker products (e.g., see Lütkepohl, 1997 for an overview).

*Proof of Equation* (18a):

A.3 $$\begin{aligned} \mathrm {E}({\mathbf {V}} \mid do({\mathbf {x}}))&=({\mathbf{I}}_n-{\mathbf{I}}_{{\mathcal {N}}}{\mathbf {C}})^{-1} {\mathbf {1}}_{{\mathcal {I}}}{\mathbf {x}}\nonumber \\ \Rightarrow \quad \textsf {d}\mathrm {E}({\mathbf {V}} \mid do({\mathbf {x}}))&=\textsf {d}\big [({\mathbf{I}}_n-{\mathbf{I}}_{{\mathcal {N}}} {\mathbf {C}})^{-1}{\mathbf {1}}_{{\mathcal {I}}}{\mathbf {x}}\big ] =({\mathbf{I}}_n-{\mathbf{I}}_{{\mathcal {N}}}{\mathbf {C}})^{-1}{\mathbf{I}}_{{\mathcal {N}}}[\textsf {d}{\mathbf {C}}]({\mathbf{I}}_n-{\mathbf{I}}_{{\mathcal {N}}}{\mathbf {C}})^{-1} {\mathbf {1}}_{{\mathcal {I}}}{\mathbf {x}}\nonumber \\ \Leftrightarrow \quad {{{\,\mathrm{\textsf {vec}}\,}}} \big (\textsf {d}\mathrm {E}({\mathbf {V}} \mid do({\mathbf {x}}))\big )&={{{\,\mathrm{\textsf {vec}}\,}}} \big (({\mathbf{I}}_n-{\mathbf{I}}_{{\mathcal {N}}} {\mathbf {C}})^{-1}{\mathbf{I}}_{{\mathcal {N}}}[\textsf {d}{\mathbf {C}}]({\mathbf{I}}_n -{\mathbf{I}}_{{\mathcal {N}}}{\mathbf {C}})^{-1}{\mathbf {1}}_{{\mathcal {I}}} {\mathbf {x}}\big )\nonumber \\ \Leftrightarrow \quad \textsf {d}\mathrm {E}({\mathbf {V}} \mid do({\mathbf {x}})) \big )&=\big (({\mathbf {x}}^\intercal {\mathbf {1}}_{{\mathcal {I}}}^\intercal ({\mathbf{I}}_n-{\mathbf{I}}_{{\mathcal {N}}}{\mathbf {C}})^{-\intercal })\otimes (({\mathbf{I}}_n -{\mathbf{I}}_{{\mathcal {N}}}{\mathbf {C}})^{-1}{\mathbf{I}}_{{\mathcal {N}}})\big ) \textsf {d}{{{\,\mathrm{\textsf {vec}}\,}}} {\mathbf {C}}\nonumber \\ \Leftrightarrow \quad \textsf {d}\mathrm {E}({\mathbf {V}} \mid do({\mathbf {x}}))\big )&=\underbrace{\big (({\mathbf {x}}^\intercal {\mathbf {1}}_{{\mathcal {I}}}^\intercal ({\mathbf{I}}_n -{\mathbf{I}}_{{\mathcal {N}}}{\mathbf {C}})^{-\intercal }) \otimes (({\mathbf{I}}_n-{\mathbf{I}}_{{\mathcal {N}}}{\mathbf {C}})^{-1} {\mathbf{I}}_{{\mathcal {N}}})\big )\frac{\partial {{{\,\mathrm{\textsf {vec}}\,}}} {\mathbf {C}}}{\partial {\varvec{\theta }}^\intercal }}_{=\frac{\partial \mathrm {E}({\mathbf {V}} \mid do({\mathbf {x}}))}{\partial {\varvec{\theta }}^\intercal }}\textsf {d}{\varvec{\theta }} \end{aligned}$$

Note that ${{{\,\mathrm{\textsf {vec}}\,}}}\,\, \textsf {d}{\mathbf {C}}=\frac{\partial {{{\,\mathrm{\textsf {vec}}\,}}} {\mathbf {C}}}{\partial {\varvec{\theta }}^\intercal }\textsf {d}{\varvec{\theta }}$ holds by definition and that each entry of the matrix ${\mathbf {C}}={\mathbf {C}}({\varvec{\theta }})$ is either equal to a single element of ${\varvec{\theta }}$ or equal to zero. Thus, the $n^2 \times p$ Jacobian matrix $\frac{\partial {{{\,\mathrm{\textsf {vec}}\,}}} {\mathbf {C}}}{\partial {\varvec{\theta }}^\intercal }$ is a zero-one matrix.

*Proof of Equation* (18b):

A.4 $$\begin{aligned} \mathrm {V}({\mathbf {V}} \mid do({\mathbf {x}}))&=({\mathbf{I}}_n-{\mathbf{I}}_{{\mathcal {N}}}{\mathbf {C}})^{-1} {\mathbf{I}}_{{\mathcal {N}}}{\varvec{\Psi }}{\mathbf{I}}_{{\mathcal {N}}}({\mathbf{I}}_n -{\mathbf{I}}_{{\mathcal {N}}}{\mathbf {C}})^{-\intercal }\nonumber \\ \Rightarrow \quad \textsf {d}\mathrm {V}({\mathbf {V}} \mid do({\mathbf {x}}))&=\textsf {d}\big [({\mathbf{I}}_n-{\mathbf{I}}_{{\mathcal {N}}} {\mathbf {C}})^{-1}{\mathbf{I}}_{{\mathcal {N}}}{\varvec{\Psi }}{\mathbf{I}}_{{\mathcal {N}}} ({\mathbf{I}}_n-{\mathbf{I}}_{{\mathcal {N}}}{\mathbf {C}})^{-\intercal }\big ]\nonumber \\&=({\mathbf{I}}_n-{\mathbf{I}}_{{\mathcal {N}}}{\mathbf {C}})^{-1}{\mathbf{I}}_{{\mathcal {N}}} [\textsf {d}{\mathbf {C}}]({\mathbf{I}}_n-{\mathbf{I}}_{{\mathcal {N}}}{\mathbf {C}})^{-1} {\mathbf{I}}_{{\mathcal {N}}}{\varvec{\Psi }}{\mathbf{I}}_{{\mathcal {N}}}({\mathbf{I}}_n-{\mathbf{I}}_{{\mathcal {N}}} {\mathbf {C}})^{-\intercal }\nonumber \\&\quad +({\mathbf{I}}_n-{\mathbf{I}}_{{\mathcal {N}}}{\mathbf {C}})^{-1}{\mathbf{I}}_{{\mathcal {N}}} [\textsf {d}{\varvec{\Psi }}] {\mathbf{I}}_{{\mathcal {N}}}({\mathbf{I}}_n-{\mathbf{I}}_{{\mathcal {N}}} {\mathbf {C}})^{-\intercal }\nonumber \\&\quad +({\mathbf{I}}_n-{\mathbf{I}}_{{\mathcal {N}}}{\mathbf {C}})^{-1}{\mathbf{I}}_{{\mathcal {N}}}{\varvec{\Psi }} {\mathbf{I}}_{{\mathcal {N}}}({\mathbf{I}}_n-{\mathbf{I}}_{{\mathcal {N}}}{\mathbf {C}})^{-\intercal } [\textsf {d}{\mathbf {C}}^{\intercal }]{\mathbf{I}}_{{\mathcal {N}}}({\mathbf{I}}_n-{\mathbf{I}}_{{\mathcal {N}}} {\mathbf {C}})^{-\intercal } \end{aligned}$$

Vectorizing Eq. (A.4) yields the following term for ${{{\,\mathrm{\textsf {vec}}\,}}}\,\, \textsf {d}\mathrm {V}({\mathbf {V}} \mid do({\mathbf {x}}))$:

A.5 $$\begin{aligned}&({\mathbf{I}}_{n^2}+{\mathbf {K}}_n)\big [(({\mathbf{I}}_n-{\mathbf{I}}_{{\mathcal {N}}} {\mathbf {C}})^{-1}{\mathbf{I}}_{{\mathcal {N}}}{\varvec{\Psi }}{\mathbf{I}}_{{\mathcal {N}}}) \otimes {\mathbf{I}}_n\big ]\big [({\mathbf{I}}_n-{\mathbf{I}}_{{\mathcal {N}}}{\mathbf {C}})^{-\intercal } \otimes (({\mathbf{I}}_n-{\mathbf{I}}_{{\mathcal {N}}}{\mathbf {C}})^{-1}{\mathbf{I}}_{{\mathcal {N}}}) \big ]{{{\,\mathrm{\textsf {vec}}\,}}}\,\, \textsf {d}{\mathbf {C}}\nonumber \\ +&\big [({\mathbf{I}}_n-{\mathbf{I}}_{{\mathcal {N}}}{\mathbf {C}})^{-1}\otimes ({\mathbf{I}}_n-{\mathbf{I}}_{{\mathcal {N}}}{\mathbf {C}})^{-1}\big ]({\mathbf{I}}_{{\mathcal {N}}} \otimes {\mathbf{I}}_{{\mathcal {N}}}){{{\,\mathrm{\textsf {vec}}\,}}}\,\, \textsf {d}{\varvec{\Psi }} \end{aligned}$$

Where ${\mathbf {K}}_n$ denotes the commutation matrix for $n \times n$ matrices (Magnus & Neudecker, 1979). For simplicity of notation, we define the following $n^2 \times n^2$ matrices:

$$\begin{aligned} {\mathbf {G}}_{2,\mathbf {C}}&:=({\mathbf{I}}_{n^2}+{\mathbf {K}}_n) \big [(({\mathbf{I}}_n-{\mathbf{I}}_{{\mathcal {N}}}{\mathbf {C}})^{-1}{\mathbf{I}}_{{\mathcal {N}}} {\varvec{\Psi }}{\mathbf{I}}_{{\mathcal {N}}})\otimes {\mathbf{I}}_n\big ]\big [({\mathbf{I}}_n-{\mathbf{I}}_{{\mathcal {N}}} {\mathbf {C}})^{-\intercal } \otimes (({\mathbf{I}}_n-{\mathbf{I}}_{{\mathcal {N}}} {\mathbf {C}})^{-1}{\mathbf{I}}_{{\mathcal {N}}})\big ]\\ {\mathbf {G}}_{2,\varvec{\Psi }}&:=\big [({\mathbf{I}}_n-{\mathbf{I}}_{{\mathcal {N}}} {\mathbf {C}})^{-1}\otimes ({\mathbf{I}}_n-{\mathbf{I}}_{{\mathcal {N}}}{\mathbf {C}})^{-1}\big ]({\mathbf{I}}_{{\mathcal {N}}} \otimes {\mathbf{I}}_{{\mathcal {N}}}) \end{aligned}$$

Substituting ${\mathbf {G}}_{2,\mathbf {C}}$ and ${\mathbf {G}}_{2,\varvec{\Psi }}$ into the expression for ${{{\,\mathrm{\textsf {vec}}\,}}}\,\, \textsf {d}\mathrm {V}({\mathbf {V}} \mid do({\mathbf {x}}))$ yields:

A.6 $$\begin{aligned} {{{\,\mathrm{\textsf {vec}}\,}}}\,\, \textsf {d}\mathrm {V}({\mathbf {V}} \mid do({\mathbf {x}}))&=\underbrace{\big [{\mathbf {G}}_{2,\mathbf {C}} \frac{\partial {{{\,\mathrm{\textsf {vec}}\,}}}\,\, {{\mathbf {C}}}}{\partial {\varvec{\theta }}^\intercal }+{\mathbf {G}}_{2,\varvec{\Psi }}\frac{\partial {{{\,\mathrm{\textsf {vec}}\,}}}\,\, {{\varvec{\Psi }}}}{\partial {\varvec{\theta }}^\intercal }\big ]}_{=\frac{\partial {{{\,\mathrm{\textsf {vec}}\,}}}\,\, \mathrm {V}({\mathbf {V}} \mid do({\mathbf {x}}))}{\partial {\varvec{\theta }}^\intercal }}\textsf {d}{\varvec{\theta }} \end{aligned}$$

Note that ${{{\,\mathrm{\textsf {vec}}\,}}}\,\,\textsf {d}{\varvec{\Psi }}=\frac{\partial {{\,\mathrm{\textsf {vec}}\,}}{{\varvec{\Psi }}}}{\partial {\varvec{\theta }}^\intercal }\textsf {d}{\varvec{\theta }}$ holds by definition and each entry of the matrix ${\varvec{\Psi }}={\varvec{\Psi }} ({\varvec{\theta }})$ is either equal to a single element of ${\varvec{\theta }}$ or equal to zero. Thus, the $n^2 \times p$ Jacobian matrix $\frac{\partial {{{\,\mathrm{\textsf {vec}}\,}}}\,\, {\varvec{\Psi }}}{\partial {\varvec{\theta }}^\intercal }$ is a zero-one matrix. Since $\mathrm {V}({\mathbf {V}} \mid do({\mathbf {x}}))$ is symmetric, one oftentimes works with the half-vectorized version, given by:

A.7 $$\begin{aligned} {{{\,\mathrm{\textsf {vech}}\,}}}\, \textsf {d}\mathrm {V}({\mathbf {V}} \mid do({\mathbf {x}}))&=\underbrace{{\mathbf {L}}_n\big [{\mathbf {G}}_{2,\mathbf {C}} \frac{\partial {{{\,\mathrm{\textsf {vec}}\,}}}\,\,{{\mathbf {C}}}}{\partial {\varvec{\theta }}^\intercal }+{\mathbf {G}}_{2,\varvec{\Psi }}\frac{\partial {{{\,\mathrm{\textsf {vec}}\,}}}\,\,{{\varvec{\Psi }}}}{\partial {\varvec{\theta }}^\intercal }\big ]}_{=\frac{\partial {{{\,\mathrm{\textsf {vech}}\,}}} \mathrm {V}({\mathbf {V}} \mid do({\mathbf {x}}))}{\partial {\varvec{\theta }}^\intercal }}\textsf {d}{\varvec{\theta }} \end{aligned}$$

Where ${\mathbf {L}}_n$ denotes the elimination matrix for $n \times n$ matrices Magnus and Neudecker (1980).

*Proof of Equation* (18c): We treat the interventional pdf $f({\mathbf {v}}_{{\mathcal {N}}}\mid do({\mathbf {x}}))$ as a function $\varphi$ of the interventional mean vector and the interventional covariance matrix:

A.8a $$\begin{aligned} \varphi ({\varvec{\mu }}_{{\mathcal {N}}},{\varvec{\Sigma }}_{{\mathcal {N}}})&=(2\pi )^{-\frac{n-K_x}{2}}|{\varvec{\Sigma }}_{{\mathcal {N}}}|^{-\frac{1}{2}} \times \exp \left( -\frac{1}{2}({\mathbf {v}}_{{\mathcal {N}}} -{\varvec{\mu }}_{{\mathcal {N}}})^\intercal {\varvec{\Sigma }}_{{\mathcal {N}}}^{-1} ({\mathbf {v}}_{{\mathcal {N}}}-{\varvec{\mu }}_{{\mathcal {N}}})\right) \end{aligned}$$

A.8b $$\begin{aligned} {\varvec{\mu }}_{{\mathcal {N}}}:&={\mathbf {1}}_{{\mathcal {N}}}^\intercal \mathrm {E}({\mathbf {V}} \mid do({\mathbf {x}}))\quad , \quad {\varvec{\Sigma }}_{{\mathcal {N}}}:={\mathbf {1}}_{{\mathcal {N}}}^\intercal \mathrm {V}({\mathbf {V}} \mid do({\mathbf {x}})){\mathbf {1}}_{{\mathcal {N}}} \end{aligned}$$

Further, we treat $\varphi$ as a product of two functions, that is, $\varphi =\varphi _1\cdot \varphi _2$, with:

A.9a $$\begin{aligned} \varphi _1({\varvec{\Sigma }}_{{\mathcal {N}}})&:=(2\pi )^{-\frac{n-K_x}{2}} |{\varvec{\Sigma }}_{{\mathcal {N}}}|^{-\frac{1}{2}} \end{aligned}$$

A.9b $$\begin{aligned} \varphi _2({\varvec{\mu }}_{{\mathcal {N}}},{\varvec{\Sigma }}_{{\mathcal {N}}})&:= \exp \left( -\frac{1}{2}({\mathbf {v}}_{{\mathcal {N}}} -{\varvec{\mu }}_{{\mathcal {N}}})^\intercal {\varvec{\Sigma }}_{{\mathcal {N}}}^{-1} ({\mathbf {v}}_{{\mathcal {N}}}-{\varvec{\mu }}_{{\mathcal {N}}})\right) \end{aligned}$$

We display $\varphi$ from Eq. (A.8a) as a function of $\varphi _1$ and $\varphi _2$ and apply the product rule, yielding:

A.10 $$\begin{aligned} \textsf {d}\varphi&= \textsf {d}[\varphi _1\cdot \varphi _2]=[\textsf {d}\varphi _1]\cdot \varphi _2+\varphi _1\cdot [\textsf {d}\varphi _2] \end{aligned}$$

Both $\varphi _1$ and $\varphi _2$ are composite functions:

A.11 $$\begin{aligned} \varphi _1&=g_1(f_1({\varvec{\Sigma }}_{{\mathcal {N}}})), \quad \varphi _2=h_2(g_2[{\mathbf {f}}_{21}({\varvec{\mu }}_{{\mathcal {N}}}), {\mathbf {f}}_{22}({\varvec{\Sigma }}_{{\mathcal {N}}})]) \end{aligned}$$

with:

A.12a $$\begin{aligned} f_1({\varvec{\Sigma }}_{{\mathcal {N}}})&=|{\varvec{\Sigma }}_{{\mathcal {N}}}|, \quad {\mathbb {R}}^{n \times n} \mapsto {\mathbb {R}} \end{aligned}$$

A.12b $$\begin{aligned} g_1(f_1)&=(2\pi )^{-\frac{n-K_x}{2}}f_1^{-\frac{1}{2}}, \quad {\mathbb {R}} \mapsto {\mathbb {R}}\end{aligned}$$

A.12c $$\begin{aligned} {\mathbf {f}}_{21}({\varvec{\mu }}_{{\mathcal {N}}})&=({\mathbf {v}}_{{\mathcal {N}}} -{\varvec{\mu }}_{{\mathcal {N}}}), \quad {\mathbb {R}}^{n-K_x} \mapsto {\mathbb {R}}^{n-K_x} \end{aligned}$$

A.12d $$\begin{aligned} {\mathbf {f}}_{22}({\varvec{\Sigma }}_{{\mathcal {N}}})&= {\varvec{\Sigma }}_{{\mathcal {N}}}^{-1}, \quad {\mathbb {R}}^{n \times n} \mapsto {\mathbb {R}}^{n \times n} \end{aligned}$$

A.12e $$\begin{aligned} g_2({\mathbf {f}}_{21},{\mathbf {f}}_{22})&={\mathbf {f}}_{21}^\intercal {\mathbf {f}}_{22}{\mathbf {f}}_{21}, \quad {\mathbb {R}}^{n-K_x} \times {\mathbb {R}}^{n \times n} \mapsto {\mathbb {R}}\end{aligned}$$

A.12f $$\begin{aligned} h_2(g_2)&=\text {exp}(-\frac{1}{2}g_2), \quad {\mathbb {R}} \mapsto {\mathbb {R}} \end{aligned}$$

The differentials of $\varphi _1$ and $\varphi _2$ are computed using Cauchy’s invariance (Magnus & Neudecker, 1999). We start with $\varphi _1$ and compute the differential of the innermost function $f_1({\varvec{\Sigma }}_{{\mathcal {N}}})$:

A.13 $$\begin{aligned} \textsf {d}f_{1}&=|{\varvec{\Sigma }}_{{\mathcal {N}}}|\text {tr} ({\varvec{\Sigma }}_{{\mathcal {N}}}^{-1}\textsf {d}{\varvec{\Sigma }}_{{\mathcal {N}}})=|{\varvec{\Sigma }}_{{\mathcal {N}}}|{{{\,\mathrm{\textsf {vec}}\,}}}\,\, ({\varvec{\Sigma }}_{{\mathcal {N}}}^{-\intercal })^\intercal {{{\,\mathrm{\textsf {vec}}\,}}}\,\, \textsf {d}{\varvec{\Sigma }}_{{\mathcal {N}}}=f_1{{{\,\mathrm{\textsf {vec}}\,}}}\,\, ({\varvec{\Sigma }}_{{\mathcal {N}}}^{-1})^\intercal {{{\,\mathrm{\textsf {vec}}\,}}}\,\, \textsf {d}{\varvec{\Sigma }}_{{\mathcal {N}}} \end{aligned}$$

Next, we obtain the differential of $g_1$ with respect to $f_1$:

A.14 $$\begin{aligned} \frac{\textsf {d}g_1}{\textsf {d}f_1}&=-\frac{1}{2}(2\pi )^{-\frac{n-K_x}{2}}f_1^{-\frac{3}{2}} =-\frac{1}{2}\varphi _1f_1^{-1} \Rightarrow \textsf {d}g_1=-\frac{1}{2}\varphi _1 f_1^{-1} \textsf {d}f_1 \end{aligned}$$

Plugging in Eq. (A.13) into Eq. (A.14) yields:

A.15 $$\begin{aligned} \textsf {d}\varphi _1=\frac{\textsf {d}g_1}{\textsf {d}f_1}\textsf {d}f_1&=-\frac{1}{2}\varphi _1f_1^{-1} f_1 {{{\,\mathrm{\textsf {vec}}\,}}}\,\, ({\varvec{\Sigma }}_{{\mathcal {N}}}^{-1})^\intercal {{{\,\mathrm{\textsf {vec}}\,}}}\,\, \textsf {d}{\varvec{\Sigma }}_{{\mathcal {N}}}=-\frac{1}{2}\varphi _1 {{{\,\mathrm{\textsf {vec}}\,}}}\,\, ({\varvec{\Sigma }}_{{\mathcal {N}}}^{-1})^\intercal {{{\,\mathrm{\textsf {vec}}\,}}}\,\,\textsf {d}{\varvec{\Sigma }}_{{\mathcal {N}}} \end{aligned}$$

For $\varphi _2$, we start with the differentials of ${\mathbf {f}}_{21}({\varvec{\mu }}_{{\mathcal {N}}})$ and ${\mathbf {f}}_{22}({\varvec{\Sigma }}_{{\mathcal {N}}})$:

A.16a $$\begin{aligned} \textsf {d}{\mathbf {f}}_{21}&=\textsf {d}({\mathbf {v}}_{{\mathcal {N}}} -{\varvec{\mu }}_{{\mathcal {N}}})=-\textsf {d}{\varvec{\mu }}_{{\mathcal {N}}} \Rightarrow \frac{\partial {\mathbf {f}}_{21}}{\partial {\varvec{\mu }}_{{\mathcal {N}}}^\intercal }=-{\mathbf{I}}_{n-K_x} \end{aligned}$$

A.16b $$\begin{aligned} \textsf {d}{\mathbf {f}}_{22}&=\textsf {d}{\varvec{\Sigma }}_{{\mathcal {N}}}^{-1}= -{\varvec{\Sigma }}_{{\mathcal {N}}}^{-1} [\textsf {d}{\varvec{\Sigma }}_{{\mathcal {N}}}] {\varvec{\Sigma }}_{{\mathcal {N}}}^{-1} \Rightarrow {{{\,\mathrm{\textsf {vec}}\,}}}\,\, \textsf {d}{\mathbf {f}}_{22}=-({\varvec{\Sigma }}_{{\mathcal {N}}}^{-1}\otimes {\varvec{\Sigma }}_{{\mathcal {N}}}^{-1}){{{\,\mathrm{\textsf {vec}}\,}}}\,\, \textsf {d}{\varvec{\Sigma }}_{{\mathcal {N}}} \end{aligned}$$

Next, we obtain the total differential of $g_2$ by applying the product rule twice:

A.17 $$\begin{aligned} \textsf {d}g_2&=\textsf {d}[{\mathbf {f}}_{21}^\intercal {\mathbf {f}}_{22} {\mathbf {f}}_{21}]=[\textsf {d}{\mathbf {f}}_{21}^\intercal ]{\mathbf {f}}_{22} {\mathbf {f}}_{21}+{\mathbf {f}}_{21}^\intercal [\textsf {d}{\mathbf {f}}_{22}] {\mathbf {f}}_{21}+{\mathbf {f}}_{21}^\intercal {\mathbf {f}}_{22} \textsf {d}{\mathbf {f}}_{21}\nonumber \\&=2{\mathbf {f}}_{21}^\intercal {\mathbf {f}}_{22}\textsf {d}{\mathbf {f}}_{21}+({\mathbf {f}}_{21}^\intercal \otimes {\mathbf {f}}_{21}^\intercal ) {{{\,\mathrm{\textsf {vec}}\,}}}\,\, \textsf {d}{\mathbf {f}}_{22} \end{aligned}$$

The last mapping that is applied in this chain is $h_2(g_2)$, which is a scalar function of a scalar argument:

A.18 $$\begin{aligned} \frac{\textsf {d}h_2}{\textsf {d}g_2}&=\frac{\textsf {d}}{\textsf {d}g_2} \text {exp}(-\frac{1}{2}g_2)=-\frac{1}{2}\text {exp}(-\frac{1}{2}g_2) =-\frac{1}{2}\varphi _2 \quad \Rightarrow \ \textsf {d}h_2=-\frac{1}{2}\varphi _2 \textsf {d}g_2 \end{aligned}$$

Plugging in (A.17) into (A.18) yields:

A.19 $$\begin{aligned} \textsf {d}\varphi _2&=\frac{\textsf {d}h_2}{\textsf {d}g_2}\textsf {d}g_2=-\frac{1}{2}\varphi _2\left[ 2{\mathbf {f}}_{21}^\intercal {\mathbf {f}}_{22} \textsf {d}{\mathbf {f}}_{21}+({\mathbf {f}}_{21}^\intercal \otimes {\mathbf {f}}_{21}^\intercal ){{{\,\mathrm{\textsf {vec}}\,}}}\,\, \textsf {d}{\mathbf {f}}_{22} \right] \end{aligned}$$

Plugging in Eqs. (A.16) into (A.19) yields:

A.20 $$\begin{aligned} \textsf {d}\varphi _2&=-\frac{1}{2}\varphi _2 (2{\mathbf {f}}_{21}^\intercal {\mathbf {f}}_{22} {[}-\textsf {d}{\varvec{\mu }}_{{\mathcal {N}}}]+({\mathbf {f}}_{21}^\intercal \otimes {\mathbf {f}}_{21}^\intercal )[-({\varvec{\Sigma }}_{{\mathcal {N}}}^{-1} \otimes {\varvec{\Sigma }}_{{\mathcal {N}}}^{-1}) {{{\,\mathrm{\textsf {vec}}\,}}}\,\, \textsf {d}{\varvec{\Sigma }}_{{\mathcal {N}}}])\nonumber \\&=\varphi _2\big [{\mathbf {f}}_{21}^\intercal {\mathbf {f}}_{22}\textsf {d}{\varvec{\mu }}_{{\mathcal {N}}}+\frac{1}{2}({\mathbf {f}}_{21}^\intercal \otimes {\mathbf {f}}_{21}^\intercal )({\varvec{\Sigma }}_{{\mathcal {N}}}^{-1}\otimes {\varvec{\Sigma }}_{{\mathcal {N}}}^{-1}){{{\,\mathrm{\textsf {vec}}\,}}}\,\, \textsf {d}{\varvec{\Sigma }}_{{\mathcal {N}}}\big ] \end{aligned}$$

We now insert Eqs. (A.9a), (A.9b), (A.15), and (A.20) into Eq. (A.10):

A.21 $$\begin{aligned}&\textsf {d}\varphi =\textsf {d}f({\mathbf {v}}_{{\mathcal {N}}} \mid do({\mathbf {x}}))\nonumber \\&=\bigg (-\frac{1}{2}\varphi _1 {{{\,\mathrm{\textsf {vec}}\,}}}\,\, ({\varvec{\Sigma }}_{{\mathcal {N}}}^{-1})^\intercal {{{\,\mathrm{\textsf {vec}}\,}}}\,\, \textsf {d}{\varvec{\Sigma }}_{{\mathcal {N}}}\bigg )\varphi _2\nonumber \\&\qquad +\varphi _1\cdot \bigg (\varphi _2\big [{\mathbf {f}}_{21}^\intercal {\mathbf {f}}_{22} \textsf {d}{\varvec{\mu }}_{{\mathcal {N}}}+\frac{1}{2}({\mathbf {f}}_{21}^\intercal \otimes {\mathbf {f}}_{21}^\intercal )({\varvec{\Sigma }}_{{\mathcal {N}}}^{-1} \otimes {\varvec{\Sigma }}_{{\mathcal {N}}}^{-1}){{{\,\mathrm{\textsf {vec}}\,}}}\,\, \textsf {d}{\varvec{\Sigma }}_{{\mathcal {N}}}\big ]\bigg )\nonumber \\&=\varphi _1 \varphi _2\left[ {\mathbf {f}}_{21}^\intercal {\mathbf {f}}_{22} \textsf {d}{\varvec{\mu }}_{{\mathcal {N}}}+\left( -\frac{1}{2}{{{\,\mathrm{\textsf {vec}}\,}}}\,\, ({\varvec{\Sigma }}_{{\mathcal {N}}}^{-1})^\intercal +\frac{1}{2} ({\mathbf {f}}_{21}^\intercal \otimes {\mathbf {f}}_{21}^\intercal ) ({\varvec{\Sigma }}_{{\mathcal {N}}}^{-1}\otimes {\varvec{\Sigma }}_{{\mathcal {N}}}^{-1}) \right) {{{\,\mathrm{\textsf {vec}}\,}}}\,\, \textsf {d}{\varvec{\Sigma }}_{{\mathcal {N}}}\right] \nonumber \\&{=}\varphi \left[ ({\mathbf {v}}_{{\mathcal {N}}} {-}{\varvec{\mu }}_{{\mathcal {N}}})^\intercal {\varvec{\Sigma }}_{{\mathcal {N}}}^{{-}1}\textsf {d}{\varvec{\mu }}_{{\mathcal {N}}}{+}\frac{1}{2}\big (\big [({\mathbf {v}}_{{\mathcal {N}}} {-}{\varvec{\mu }}_{{\mathcal {N}}})^\intercal \otimes ({\mathbf {v}}_{{\mathcal {N}}}\right. \left. {-}{\varvec{\mu }}_{{\mathcal {N}}})^\intercal \big ] ({\varvec{\Sigma }}_{{\mathcal {N}}}^{{-}1}\otimes {\varvec{\Sigma }}_{{\mathcal {N}}}^{{-}1})\right. \left. {-}{{{\,\mathrm{\textsf {vec}}\,}}} ({\varvec{\Sigma }}_{{\mathcal {N}}}^{{-}1})^\intercal \big ) {{{\,\mathrm{\textsf {vec}}\,}}} \textsf {d}{\varvec{\Sigma }}_{{\mathcal {N}}}\right] \nonumber \\&\qquad =f({\mathbf {v}}_{{\mathcal {N}}}\mid do({\mathbf {x}}))\big [{\mathbf {G}}_{3,\varvec{\mu }},{\mathbf {G}}_{3,\varvec{\Sigma }} \big ]\begin{pmatrix} \textsf {d}{\varvec{\mu }}_{{\mathcal {N}}}\\ {{{\,\mathrm{\textsf {vec}}\,}}}\,\, \textsf {d}{\varvec{\Sigma }}_{{\mathcal {N}}} \end{pmatrix} \end{aligned}$$

Where we have resubstituted the expressions for $\varphi _1$, $\varphi _2$, $\varphi$, ${\mathbf {f}}_{21}$, ${\mathbf {f}}_{22}$ and introduced the following terms for simplicity of notation:

$$\begin{aligned} {\mathbf {G}}_{3,\varvec{\mu }}&:=({\mathbf {v}}_{{\mathcal {N}}}-{\varvec{\mu }}_{{\mathcal {N}}})^\intercal {\varvec{\Sigma }}_{{\mathcal {N}}}^{-1}\\ {\mathbf {G}}_{3,\varvec{\Sigma }}&:=\frac{1}{2}\big (\big [({\mathbf {v}}_{{\mathcal {N}}}-{\varvec{\mu }}_{{\mathcal {N}}})^\intercal \otimes ({\mathbf {v}}_{{\mathcal {N}}}-{\varvec{\mu }}_{{\mathcal {N}}})^\intercal \big ]({\varvec{\Sigma }}_{{\mathcal {N}}}^{-1}\otimes {\varvec{\Sigma }}_{{\mathcal {N}}}^{-1})- {{{\,\mathrm{\textsf {vec}}\,}}}\,\, ({\varvec{\Sigma }}_{{\mathcal {N}}}^{-1})^\intercal \big ) \end{aligned}$$

From the equations stated in Eq. (A.8b), it immediately follows:

A.22 $$\begin{aligned} \textsf {d}{\varvec{\mu }}_{{\mathcal {N}}}&=\textsf {d}[{\mathbf {1}}_{{\mathcal {N}}}^\intercal \mathrm {E}({\mathbf {V}} \mid do({\mathbf {x}}))] ={\mathbf {1}}_{{\mathcal {N}}}^\intercal \textsf {d}\mathrm {E}({\mathbf {V}} \mid do({\mathbf {x}})) \end{aligned}$$

A.23 $$\begin{aligned} {{{\,\mathrm{\textsf {vec}}\,}}}\,\, \textsf {d}{\varvec{\Sigma }}_{{\mathcal {N}}}&= {{{\,\mathrm{\textsf {vec}}\,}}}\,\, \textsf {d}[{\mathbf {1}}_{{\mathcal {N}}}^\intercal \mathrm {V}({\mathbf {V}} \mid do({\mathbf {x}})){\mathbf {1}}_{{\mathcal {N}}}] =({\mathbf {1}}_{{\mathcal {N}}}^\intercal \otimes {\mathbf {1}}_{{\mathcal {N}}}^\intercal ) {{{\,\mathrm{\textsf {vec}}\,}}}\,\,\textsf {d}\mathrm {V}({\mathbf {V}} \mid do({\mathbf {x}})) \end{aligned}$$

Using Eqs. (A.3) and (A.4), we obtain the final result:

A.24 $$\begin{aligned}&\textsf {d}f({\mathbf {v}}_{{\mathcal {N}}}\mid do({\mathbf {x}}))=\nonumber \\&\underbrace{f({\mathbf {v}}_{{\mathcal {N}}}\mid do({\mathbf {x}}))\big [{\mathbf {G}}_{3,\varvec{\mu }},{\mathbf {G}}_{3,\varvec{\Sigma }}\big ] \begin{pmatrix} {\mathbf {1}}_{{\mathcal {N}}}^\intercal \big (({\mathbf {x}}^\intercal {\mathbf {1}}_{{\mathcal {I}}}^\intercal ({\mathbf{I}}_n-{\mathbf{I}}_{{\mathcal {N}}}{\mathbf {C}})^{-\intercal })\otimes (({\mathbf{I}}_n-{\mathbf{I}}_{{\mathcal {N}}}{\mathbf {C}})^{-1}{\mathbf{I}}_{{\mathcal {N}}})\big )\frac{\partial {{{\,\mathrm{\textsf {vec}}\,}}}\,\, {\mathbf {C}}}{\partial {\varvec{\theta }}^\intercal }\\ ({\mathbf {1}}_{{\mathcal {N}}}^\intercal \otimes {\mathbf {1}}_{{\mathcal {N}}}^\intercal )\left( {\mathbf {G}}_{2,\mathbf {C}} \frac{\partial {{{\,\mathrm{\textsf {vec}}\,}}}\,\,{{\mathbf {C}}}}{\partial {\varvec{\theta }}^\intercal }+{\mathbf {G}}_{2,\varvec{\Psi }}\frac{\partial {{{\,\mathrm{\textsf {vec}}\,}}}\,\, {{\varvec{\Psi }}}}{\partial {\varvec{\theta }}^\intercal }\right) \end{pmatrix}}_{\frac{\partial f({\mathbf {v}}_{{\mathcal {N}}} \mid do({\mathbf {x}}))}{\partial {\varvec{\theta }}^\intercal }}\textsf {d}{\varvec{\theta }} \end{aligned}$$

*Proof of Equation* (18d): The general definition of $g_4$ for a vector of outcome variables ${\mathbf {Y}}$ is given in Eq. (15). The following derivation is restricted to the case of a single (scalar) outcome variable *Y*, that is, $|{\mathcal {Y}}|=K_y=1$.

A.25 $$\begin{aligned} \gamma _4:=P(y^{{low}}\le y \le y^{{up}} \mid do({\mathbf {x}}))=g_4({\varvec{\theta }}_{\gamma _4};{\mathbf {x}},y^{{low}}, y^{{up}})=\int _{y^{{low}}}^{y^{{up}}} f(y\mid do({\mathbf {x}})) \textsf {d}y \end{aligned}$$

Let *Y* be the *j*-th entry of ${\mathbf {V}}$. For simplicity of notation, we denote the scalar interventional mean and the scalar interventional variance as:

A.26a $$\begin{aligned} \mu _y=\mu _y({\varvec{\theta }})&:=\mathrm {E}(y\mid do({\mathbf {x}})) =\varvec{{{\imath }}}_{j}^\intercal \mathrm {E}({\mathbf {V}} \mid do({\mathbf {x}})) \end{aligned}$$

A.26b $$\begin{aligned} \sigma ^2_y=\sigma ^2_y({\varvec{\theta }})&:=\mathrm {V}(y\mid do({\mathbf {x}}))=\varvec{{{\imath }}}_{j}^\intercal \mathrm {V}({\mathbf {V}} \mid do({\mathbf {x}}))\varvec{{{\imath }}}_{j} \end{aligned}$$

Again, we take the derivative with respect to the entire parameter vector ${\varvec{\theta }}$.

A.27 $$\begin{aligned}&\frac{\partial }{\partial {\varvec{\theta }}^\intercal }g_4({\varvec{\theta }}; {\mathbf {x}},y^{{up}},y^{{low}})=\frac{\partial }{\partial {\varvec{\theta }}^\intercal }\int _{\frac{y^{{low}}-\mu _y({\varvec{\theta }})}{\sigma _y({\varvec{\theta }})})}^{\frac{y^{{up}}-\mu _y({\varvec{\theta }})}{\sigma _y({\varvec{\theta }})}}\phi (u)\textsf {d}u=\int _{\frac{y^{{low}} -\mu _y({\varvec{\theta }})}{\sigma _y({\varvec{\theta }})})}^{\frac{y^{{up}} -\mu _y({\varvec{\theta }})}{\sigma _y({\varvec{\theta }})}}\frac{\partial }{\partial {\varvec{\theta }}^\intercal }\phi (u)\textsf {d}u \nonumber \\&+\phi \left( \frac{y^{{up}}-\mu _y({\varvec{\theta }})}{\sigma _y({\varvec{\theta }})} \right) \frac{\partial }{\partial {\varvec{\theta }}^\intercal }\left[ \frac{y^{{up}}-\mu _y({\varvec{\theta }})}{\sigma _y({\varvec{\theta }})}\right] -\phi \left( \frac{y^{\textit{low}} -\mu _y({\varvec{\theta }})}{\sigma _y({\varvec{\theta }})}\right) \frac{\partial }{\partial {\varvec{\theta }}^\intercal }\left[ \frac{y^{{low}}-\mu _y({\varvec{\theta }})}{\sigma _y({\varvec{\theta }})}\right] \end{aligned}$$

The last equation sign of Eq. (A.27) follows from Leibniz’s rule for partial differentiation of an integral (Dieudonné, 1969). The derivative under the integral sign (first term after the last equation sign) is equal to zero since the pdf of the standard normal $\phi (u)$ is functionally independent of ${\varvec{\theta }}$. For simplicity of notation, we use $\mu _y$ and $\sigma ^2_y$ instead of $\mu _y({\varvec{\theta }})$ and $\sigma ^2_y({\varvec{\theta }})$ in the following. The two partial derivatives in the second line of Eq. (A.27) have the same structure $\varphi _3=h_3[f_{31}(\mu _y),f_{32}(\sigma _y^2)]$ and differ only in the constants $y^{{up}}$ and $y^{{low}}$. The functions below are stated for $y^{{up}}$ and are defined analogously for $y^{{low}}$ (we do not state the latter ones explicitly):

A.28a $$\begin{aligned}&f_{31}(\mu _y)=(y^{{up}}-\mu _y) \ , \ {\mathbb {R}} \mapsto {\mathbb {R}} \quad , \quad f_{32}(\sigma _y^2) ={(\sigma _y^{2})}^{-\frac{1}{2}} \ , \ {\mathbb {R}}^+ \mapsto {\mathbb {R}} ^+ \end{aligned}$$

A.28b $$\begin{aligned}&h_3(f_{31},f_{32})=f_{31}f_{32} \ , \ {\mathbb {R}}\times {\mathbb {R}}^{+} \mapsto {\mathbb {R}} \end{aligned}$$

The corresponding differentials and derivatives are given by:

A.29 $$\begin{aligned} \frac{\partial h_3}{\partial f_{31}}&={(\sigma _y^{2})}^{-\frac{1}{2}} \ , \ \frac{\partial h_3}{\partial f_{32}}=(y^{\textit{up}}-\mu _y) \ , \ \frac{\textsf {d}f_{31}}{\textsf {d}\mu _y}=-1 \ , \ \frac{\textsf {d}f_{32}}{\textsf {d}\sigma _y^2}=(-\frac{1}{2}){(\sigma _y^{2})}^{-\frac{3}{2}} \end{aligned}$$

The differential of $\varphi _3=h_3[f_{31}(\mu _y({\varvec{\theta }})),f_{32}(\sigma _y^2({\varvec{\theta }}))]$ can be evaluated as follows using the total differential, Cauchy’s invariance and the chain rule:

A.30 $$\begin{aligned} \textsf {d}\varphi _3&=\frac{\partial h_3}{\partial {\varvec{\theta }}^\intercal }\textsf {d}{\varvec{\theta }}=\left( \frac{\partial h_3}{\partial f_{31}}\frac{\partial f_{31}}{\partial \mu _y}\frac{\partial \mu _y}{\partial {\varvec{\theta }}^\intercal }+\frac{\partial h_3}{\partial f_{32}}\frac{\partial f_{32}}{\partial \sigma _y^2}\frac{\partial \sigma _y^2}{\partial {\varvec{\theta }}^\intercal } \right) \textsf {d}{\varvec{\theta }} \end{aligned}$$

Inserting Eqs. (A.28) and (A.29) into Eq. (A.30) yields the following term for $y^{{up}}$ (analogous for $y^{{low}}$):

A.31 $$\begin{aligned} \frac{\partial h_3}{\partial {\varvec{\theta }}^\intercal }&=-\frac{1}{\sigma _y}\frac{\partial \mu _y}{\partial {\varvec{\theta }}^\intercal }-\frac{1}{2\sigma _y^{2}} \left( \frac{y^{{up}}-\mu _y}{\sigma _y}\right) \frac{\partial \sigma _y^2}{\partial {\varvec{\theta }}^\intercal } \end{aligned}$$

Inserting Eqs. (A.28), (A.29), (A.30), and (A.31) into the derivative of the causal effect function $g_4$ (Eq. [A.27]) and rearranging yields:

A.32 $$\begin{aligned}&\frac{\partial }{\partial {\varvec{\theta }}^\intercal }g_4({\varvec{\theta }};x, y^{{up}},y^{{low}})=\nonumber \\&\phi \left( \frac{y^{{up}}-\mu _y}{\sigma _y} \right) \left( -\frac{1}{\sigma _y}\frac{\partial \mu _y}{\partial {\varvec{\theta }}^\intercal }-\frac{1}{2\sigma _y^{2}}\left( \frac{y^{{up}} -\mu _y}{\sigma _y}\right) \frac{\partial \sigma _y^2}{\partial {\varvec{\theta }}^\intercal }\right) \nonumber \\&-\phi \left( \frac{y^{{low}}-\mu _y}{\sigma _y}\right) \left( -\frac{1}{\sigma _y}\frac{\partial \mu _y}{\partial {\varvec{\theta }}^\intercal } -\frac{1}{2\sigma _y^{2}}\left( \frac{y^{{low}}-\mu _y}{\sigma _y}\right) \frac{\partial \sigma _y^2}{\partial {\varvec{\theta }}^\intercal }\right) =\nonumber \\&-\frac{1}{\sigma _y}\left[ \phi \left( \frac{y^{{up}}-\mu _y}{\sigma _y}\right) -\phi \left( \frac{y^{{low}}-\mu _y}{\sigma _y}\right) \right] \frac{\partial \mu _y}{\partial {\varvec{\theta }}^\intercal }\nonumber \\&-\frac{1}{2\sigma _y^{2}}\left[ \phi \left( \frac{y^{{up}}-\mu _y}{\sigma _y}\right) \left( \frac{y^{{up}}-\mu _y}{\sigma _y}\right) -\phi \left( \frac{y^{{low}}-\mu _y}{\sigma _y}\right) \left( \frac{y^{{low}}-\mu _y}{\sigma _y}\right) \right] \frac{\partial \sigma _y^2}{\partial {\varvec{\theta }}^\intercal } \end{aligned}$$

The derivatives $\frac{\partial \mu _y}{\partial {\varvec{\theta }}^\intercal }$ and $\frac{\partial \sigma _y^2}{\partial {\varvec{\theta }}^\intercal }$ are obtained from the general expressions in Eqs. (A.3) and (A.6) by selecting the corresponding rows. Row selection can be obtained by premultiplication with a selection matrix:

A.33 $$\begin{aligned}&\frac{\partial }{\partial {\varvec{\theta }}^\intercal }g_4({\varvec{\theta }};x,y^{{up}},y^{{low}}) =\big [{\mathbf {G}}_{4,\mu },{\mathbf {G}}_{4,\sigma ^2} \big ] \left( \begin{array}{rl} \varvec{{{\imath }}}_{j}^\intercal &{}\frac{\partial \mathrm {E}({\mathbf {V}} \mid do({\mathbf {x}}))}{\partial {\varvec{\theta }}^\intercal }\\ \varvec{{{\imath }}}_{(j-1)n+j}^\intercal &{}\frac{\partial {{{\,\mathrm{\textsf {vec}}\,}}}\,\, \mathrm {V}({\mathbf {V}} \mid do({\mathbf {x}}))}{\partial {\varvec{\theta }}^\intercal } \end{array}\right) \end{aligned}$$

Where the unit vector in the upper entry of the vector in Eq. (A.33) is of dimension $(n \times 1)$ and the unit vector in the lower entry is of dimension $(n^2 \times 1)$. The matrices denoted by $\mathbf {G}$ and a subscript are defined as follows:

A.34a $$\begin{aligned} {\mathbf {G}}_{4,\mu }&:= - \frac{1}{\sigma _y}\left[ \phi \left( \frac{y^{{up}}-\mu _y}{\sigma _y}\right) -\phi \left( \frac{y^{{low}}-\mu _y}{\sigma _y}\right) \right] \end{aligned}$$

A.34b $$\begin{aligned} {\mathbf {G}}_{4,\sigma ^2}&:= - \frac{1}{2\sigma _y^{2}} \left[ \phi \left( \frac{y^{{up}}-\mu _y}{\sigma _y}\right) \left( \frac{y^{{up}}-\mu _y}{\sigma _y}\right) -\phi \left( \frac{y^{{low}}-\mu _y}{\sigma _y}\right) \left( \frac{y^{{low}}-\mu _y}{\sigma _y}\right) \right] \end{aligned}$$

where $\frac{\partial \mu _y}{\partial {\varvec{\theta }}^\intercal }$ is obtained from $\frac{\partial \mathrm {E}({\mathbf {V}} \mid do({\mathbf {x}}))}{\partial {\varvec{\theta }}^\intercal }$ by selecting the *j*-th row. Since $\frac{\partial {{{\,\mathrm{\textsf {vec}}\,}}}\,\, \mathrm {V}({\mathbf {V}} \mid do({\mathbf {x}}))}{\partial {\varvec{\theta }}^\intercal }$ is a vectorized quantity, $\frac{\partial \sigma _y^2}{\partial {\varvec{\theta }}^\intercal }$ is obtained by selecting the $((j-1)n+j)$-th row. $\square$

## Footnotes

1 Exceptions from this statement include, for example Ernest and Bühlmann (2015) and Bhattacharya, Nabi, and Shpitser (2020). Furthermore, statistical procedures from related fields such as the potential outcome framework (Robins, 1986, 1987; Robins, Rotnitzky, & Zhao, 1994; Rosenbaum & Rubin, 1983; van der Laan & Rubin, 2006) or econometrics (Chernozhukov, Fernández-Val, Newey, Stouli, & Vella, 2020; Matzkin, 2015) could be adjusted such that they can be used to estimate causal quantities in the NPSEM framework.

2 However, techniques for identification (e.g., rank and order conditions) and estimation (e.g., limited information estimators) of single structural equations have been developed (c.f. Bollen, 1996; Bollen, Kolenikov, & Bauldry, 2014; Bowden & Turkington, 1985; Fisher, 1966).

3 If the listed statements are indeed true, the causal Markov assumption is implied. For a detailed discussion of the logical relation of causal assumptions encoded in graph-based models and causal assumptions from the Neyman–Rubin potential outcome framework (e.g., ignorability, SUTVA), see, for example, Holland (1988); Pearl (2009); Shpitser et al. (2020).

4 Similar concepts such as autonomy (Aldrich, 1989), exogeneity (Mouchart, Russo, & Wunsch, 2009), and invariance (Cartwright, 2009) have been discussed in the econometric literature. However, we believe that these concepts are not part of the canonical assumptions of traditional SEM as used in the social and behavioral sciences.

5 Many results derived in this paper (e.g., the moments of the interventional distribution in Eqs. (6a) and (6b) or Theorem 8) do *not* rely on multivariate normality. However, Result 3 on the distributional family of the interventional distribution requires multivariate normality.

6 Throughout this article, we use the following conventions: Sets of random variables are denoted by calligraphic letters (e.g., ${\mathcal {V}}=\{V_1,...,V_n\}$). Single random variables from a set are denoted by corresponding upper-case Latin letters (e.g., $V_i$). The column vector containing all random variables in a set is denoted by the corresponding bold Latin letter (e.g., ${\mathbf {V}}=(V_1,...,V_n)^\intercal$). Realizations of a random vector ${\mathbf {V}}$ are denoted by lower-case Latin letters (e.g., ${\mathbf {v}}$).

7 Note that bidirected edges in a causal graph (see Fig. 2 in the illustration section) represent a nonzero covariance between error terms that is due to an unobserved common cause. This convention for causal graphs is different for path diagrams from the traditional SEM literature where bidirected edges simply indicate a correlation without being specific about its origin.

8 An alternative approach to compute the distribution of an outcome variable under different (hypothetical) treatments is Robin’s (1986) G-formula. For similarities and differences between the two approaches, see, for example Hernán and Robins (2020); Pearl (2009); Pearl and Robins (1995).

9 A detailed justification that the matrix expressions in Eq. (3) adequately represent the changes to the linear system imposed by the *do*-operator is provided in the online supplementary material.

10 In practice, nonparametric estimation of multivariate distributions requires certain regularity conditions and large sample sizes due to reduced rates of convergence (as compared to parametric estimation procedures). This practical limitation will be particularly pronounced in high dimensional systems with continuous variables, a phenomenon known as the curse of dimensionality.

11 Additional estimation techniques include two- and three-stage least squares (2SLS, 3SLS; Bollen, 1996; Sargan, 1988; Theil, 1971), instrumental variables (IV; Bowden & Turkington, 1985), and generalized methods of moments (GMM; Bollen et al., 2014; Hansen, 1982; Hayashi, 2011).

12 A more detailed description of the data simulation is provided in the online supplementary material.

13 For the detailed derivation of analytic expressions and computational details, we refer the reader to the online supplementary material.

14 Put more technically, we show that the model is locally identified using a generalized version of Wald’s (1950) rank rule (Bekker et al., 1994). Given the triangular structure of the matrix of structural coefficients and the special structure of the covariance restrictions, we believe that the model is also globally identified (Hausman & Taylor, 1983; Hsiao, 1983).

15 For the detailed derivation of analytic expressions and computational details, we refer the reader to the online supplementary material.
