# Supplementary material for: Beyond the Mean: A Flexible Framework for Studying Causal Effects Using Linear Models
Source: Psychometrika. 2021 Dec 11;87(3):868–901. doi: 10.1007/s11336-021-09811-z (PMC9433367; doi:10.1007/s11336-021-09811-z)
Supplement: Supplementary file 1 — (pdf 644 KB) [file 11336_2021_9811_MOESM1_ESM.pdf]

Beyond the mean: A flexible framework for studying causal effects using linear models

–

Online Supplementary Material

Christian Gische[a] and Manuel C. Voelkle[a]

[a] Department of Psychology, Humboldt University of Berlin, Berlin, Germany.

Contact: Christian Gische
Email: christian.gische@hu-berlin.de

This material supplements the following paper:

Gische, C. & Voelkle, M. C. (2021). Beyond the mean: A flexible framework for studying causal effects using linear models. *Psychometrika*. https://doi.org/10.1007/s11336-021-09811-z.

Abstract

The main goal of this online supplementary material is to provide a comprehensive and reproducible account of the data generation, the derivation of the analytic formulae presented in the illustration section of the paper, and the numeric results. Furthermore, we provide a justification of the zero-one matrices defined to formulate *do*-type interventions using matrix algebra and complete the proof of local identification of the example model. Finally, we provide software code for the analytic derivations (using Mathematica) and the numeric calculations (using R).

Key words: causal inference, structural equation modeling, graph-based causal models, acyclic directed mixed graphs

Contents

S.1 Introduction 1

S.2 Data Generation and Estimation 1

S.3 Formulae Used in the Illustration Section of the Paper 5

S.4 Structural Equations Given *do*-Type Interventions 12

S.5 Proof of Local Identification 12

S.6 R Code 19

S.7 Mathematica Code 28

S.1 Introduction

Equations and tables from the supplementary material are labeled with S (e.g., Table S.1, Equation [S.1]) to distinguish them from those in the paper. The supplementary material is structured as follows. The data generation as used in the illustrative example is presented in Section S.2. A detailed account of the derivation of the formulae presented in the illustration section of the paper is given in Section S.3. In Section S.4, we justify the matrix operations introduced in Definition 1 of the paper to formalize the changes to a system of linear equations induced by the *do*-operator. In Section S.5, we provide the full proof of local identification of the example model. We provide computer code for the software packages R (R Core Team, 2019) that enables readers to reproduce all numerical results from the paper in Section S.6. Please note that at the time of publication, an R package with the tentative title "causalSEM" is being developed which will be made available via the Comprehensive R Archive Network (CRAN) shortly after the publication of the paper. The computer code for the computer algebra system Mathematica (Wolfram Research Inc., 2018) used to evaluate the rank of the Jacobian matrix in the proof of local identification is provided in Section S.7.

S.2 Data Generation and Estimation

The population values of the parameters used in the illustration are loosely based on prior empirical work by Ito et al. (1998). Unfortunately, we could not get access to the raw data (which were requested from the publisher), so we read off the available time series data for one healthy patient from Figure S.1 (line with empty circles). The resulting data is included in the R-code in Section S.6.

Ito et al. (1998) use a bivariate vector autoregressive model of order one (VAR[1]) to model the insulin-glucose dynamics. A VAR(1) model can be represented as follows:

$$\underbrace{\begin{pmatrix} X_{t+1} \\ Y_{t+1} \end{pmatrix}}_{\mathbf{Y}_{t+1}} = \underbrace{\begin{pmatrix} m_x \\ m_y \end{pmatrix}}_{\mathbf{m}} + \underbrace{\begin{pmatrix} c_{xx} & c_{xy} \\ c_{yx} & c_{yy} \end{pmatrix}}_{\mathbf{B}} \underbrace{\begin{pmatrix} X_t \\ Y_t \end{pmatrix}}_{\mathbf{Y}_t} + \underbrace{\begin{pmatrix} \varepsilon_{x,t+1} \\ \varepsilon_{y,t+1} \end{pmatrix}}_{\boldsymbol{\varepsilon}_{t+1}} \qquad t \in \mathbb{N} \tag{S.1}$$

Figure S.1

Time Series Data From Prior Empirical Study

(a) time series of insulin **(b)** time series of glucose

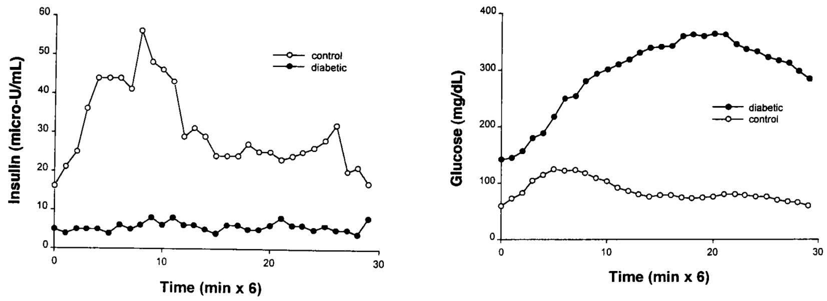

Note. Time series data ($T = 31$) for a diabetic individual (line with filled circles) and a non-diabetic individual (line with empty circles) for blood insulin levels (panel a) and blood glucose levels (panel b). Figures are reprinted with permission from Ito et al. (1998, p. 30).

As commonly done in the context of linear VAR(1) models, we impose the following set of assumptions and regularity conditions (Lütkepohl, 2005):[1]

1. innovations $\boldsymbol{\varepsilon}_t$ are independent white noise, that is, independent draws from the following normal distribution:

$$\begin{pmatrix} \varepsilon_{xt} \\ \varepsilon_{yt} \end{pmatrix} \overset{iid}{\sim} N_2\left(\begin{pmatrix} 0 \\ 0 \end{pmatrix}, \underbrace{\begin{pmatrix} \psi_x & \psi_{xy} \\ \psi_{xy} & \psi_y \end{pmatrix}}_{\boldsymbol{\Psi}_\varepsilon} \right) \qquad t \geq 2 \qquad \text{(S.2)}$$

2. regularity conditions on $\mathbf{B}$:

   (a) all eigenvalues of $\mathbf{B}$ have an absolute value less than one;

   (b) the matrix $(\mathbf{I}_2 - \mathbf{B})$ is nonsingular ($\mathbf{I}_2$ is the 2×2 identity matrix).

Under Assumptions 1. and 2. as stated above, the solution to the stochastic difference equation displayed in Equation (S.1) is covariance-stationary. We use the package vars (Pfaff, 2008) of the software R (R Core Team, 2019) to fit the bivariate VAR(1) model to the data of the non-diabetic individual (lines with empty circles in panels (a) and (b) of Figure S.1), yielding the results displayed in Table S.1.

[1]See Bollen (1987), Hamilton (1994) and Lütkepohl (2005) for formal details and other regularity conditions.

Table S.1

Estimation Results and Numeric Population Values Used in the Illustration

	m_x	m_y	c_{xx}	c_{xy}	c_{yx}	c_{yy}	ψ_x	ψ_y	ψ_{xy}
Estimate	-8.04	0.80	0.19	0.39	-0.58	1.20	18.95	44.02	3.00
Est. ASE	3.73	5.69	0.15	0.08	0.23	0.12	–	–	–
Value for Illustration	0	0	0.05	0.40	-0.60	1.20	20	40	3.00

Note. Row 1 of Table S.1 contains the estimated parameter values of the VAR(1)-model for a non-diabetic individual. Row 2 contains the estimated asymptotic standard errors (ASE). An empty cell (denoted by '–') indicates that the information is not routinely reported by the software. The third row of Table S.1 contains the values we use for the data generation underlying our numerical illustration. In the illustration we assume mean-centered variables and therefore set the intercepts m_x and m_y to zero in the last row of the table.

The mean structure was not modeled in our illustration since we assumed mean-centered data. Thus, the parameters corresponding to m_x and m_y are set to zero. The numeric population values of all remaining parameters that were used in the illustration were chosen to be relatively close to the estimated values. The choice of $c_{xx} = .05$ (instead of a value closer to $\widehat{c}_{xx} = .19$) was motivated by the fact that $\widehat{c}_{xx}$ is not significantly different from zero ($\widehat{c}_{xx} = .19$, $\widehat{s.d.}(\widehat{c}_{xx}) = .15$, $\alpha = .05$). For the estimated process the roots of the characteristic polynomial are real and have absolute values less than one ($\widehat{\lambda}_1 = .86$, $\widehat{\lambda}_2 = .52$), that is, the regularity conditions for $\widehat{\mathbf{B}}$ are met.

So far we have focused on the time series ($T = 31$) of a single person ($N = 1$). In our illustration, we assumed a panel design for N individuals and time series of fixed length $T = 3$. Thus, the situation in our illustrative example differs in three important aspects. First, the number of time points T is fixed and equal to three. Second, we simulate a situation where we draw a sample of $N = 100$ individuals i from a homogeneous population. Third, we allow for serial correlations in the error terms.

The equations of the modified model ($T = 3$) are given by:

$$
\begin{aligned}
X_{i1} &= \varepsilon_{xi1} \\
Y_{i1} &= \varepsilon_{yi1} \\
X_{i2} &= c_{xx}X_{i1} + c_{xy}Y_{i1} + \varepsilon_{xi2} \\
Y_{i2} &= c_{yx}X_{i1} + c_{yy}Y_{i1} + \varepsilon_{yi2} \\
X_{i3} &= c_{xx}X_{i2} + c_{xy}Y_{i2} + \varepsilon_{xi3} \\
Y_{i3} &= c_{yx}X_{i2} + c_{yy}Y_{i2} + \varepsilon_{yi3}\ , \qquad i = 1, ..., N
\end{aligned} \tag{S.3}
$$

Note that in Equations (S.3) we use the subscript i to indicate that the units of analysis are individuals and to clearly distinguish between quantities that vary across persons (values of observed variables and error terms) and those that are assumed not to vary across persons (structural coefficients). In the remainder, we drop the person index i for ease of presentation. Restating the system of Equations (S.3) in matrix notation yields:

$$
\underbrace{\begin{pmatrix} X_1 \\ Y_1 \\ X_2 \\ Y_2 \\ X_3 \\ Y_3 \end{pmatrix}}_{\mathbf{V}} = \underbrace{\begin{pmatrix} 0 & 0 & 0 & 0 & 0 & 0 \\ 0 & 0 & 0 & 0 & 0 & 0 \\ c_{xx} & c_{xy} & 0 & 0 & 0 & 0 \\ c_{yx} & c_{yy} & 0 & 0 & 0 & 0 \\ 0 & 0 & c_{xx} & c_{xy} & 0 & 0 \\ 0 & 0 & c_{yx} & c_{yy} & 0 & 0 \end{pmatrix}}_{\mathbf{C}} \begin{pmatrix} X_1 \\ Y_1 \\ X_2 \\ Y_2 \\ X_3 \\ Y_3 \end{pmatrix} + \underbrace{\begin{pmatrix} \varepsilon_{x1} \\ \varepsilon_{y1} \\ \varepsilon_{x2} \\ \varepsilon_{y2} \\ \varepsilon_{x3} \\ \varepsilon_{y3} \end{pmatrix}}_{\boldsymbol{\varepsilon}} \tag{S.4}
$$

The covariance matrix of the (6×1) vector of error terms $\boldsymbol{\varepsilon}$ in Equation (S.4) is given by:

$$
\mathbf{\Psi} = \begin{pmatrix} \psi_{x_1x_1} & \psi_{x_1y_1} & \psi_{x_1x_2} & 0 & 0 & 0 \\ \psi_{x_1y_1} & \psi_{y_1y_1} & 0 & \psi_{y_1y_2} & 0 & 0 \\ \psi_{x_1x_2} & 0 & \psi_{xx} & \psi_{xy} & \psi_{x_2x_3} & 0 \\ 0 & \psi_{y_1y_2} & \psi_{xy} & \psi_{yy} & 0 & \psi_{y_2y_3} \\ 0 & 0 & \psi_{x_2x_3} & 0 & \psi_{xx} & \psi_{xy} \\ 0 & 0 & 0 & \psi_{y_2y_3} & \psi_{xy} & \psi_{yy} \end{pmatrix} \tag{S.5}
$$

Where $\psi_{x_1x_1}$, $\psi_{y_1y_1}$, $\psi_{x_1y_1}$ denote the (co-)variances of the initial variables X_1 and Y_1. ψ_{xx}, ψ_{yy}, ψ_{xy} denote the (co-)variances of the error terms ε_{xt} and ε_{yt}, $t = 2, 3$. $\psi_{x_1x_2}$ and $\psi_{x_2x_3}$ denote the serial covariances between successive measurements of blood insulin levels. $\psi_{y_1y_2}$ and $\psi_{y_2y_3}$ denote the serial covariances between successive measurements of blood glucose levels. The numeric population values used for data generation in the illustration section are displayed in the first row of Table S.2. We simulate a sample of $N = 100$ values using the simulateData-function of the R package lavaan (R Core Team, 2019; Rosseel, 2012). Finally, the model is fitted to the sampled data using the sem-function of the lavaan package. The resulting point estimates are displayed in the second row of Table S.2.

Table S.2

Parameters in the Linear Graph-Based Model

	structural coefficients			
	c_{xx}	c_{xy}	c_{yx}	c_{yy}
Population	0.05	0.4	−0.6	1.2
Estimate	0.08	0.39	−0.52	1.18
Est. ASE	0.08	0.03	0.09	0.04
z-value	1.00	13.00	−5.78	29.50

	variance-covariance parameters									
	$\psi_{x_1x_1}$	$\psi_{y_1y_1}$	$\psi_{x_1y_1}$	ψ_{xx}	ψ_{yy}	ψ_{xy}	$\psi_{x_1x_2}$	$\psi_{x_2x_3}$	$\psi_{y_1y_2}$	$\psi_{y_2y_3}$
Population	131.76	632.94	254.12	20	40	3	15	2	35	10
Estimate	126.32	601.85	241.19	22.15	35.88	1.71	16.57	2.31	28.96	9.03
Est. ASE	17.02	83.23	35.83	2.58	3.93	1.93	2.71	1.78	7.07	3.29
z-value	7.42	7.23	6.73	8.59	9.13	0.89	6.11	1.30	4.10	2.74

Note. The true population values used for data simulation are reported together with the estimation results $\widehat{\boldsymbol{\theta}}^{ML}$ for the model parameters $\boldsymbol{\theta}$ (using a covariance-based maximum likelihood estimator with $N = 100$). The z-values are reported for the null hypothesis of a population quantity equal to zero. Structural coefficients are displayed in the upper part and the variance-covariance parameters are displayed in the lower part. ASE = asymptotic standard error.

S.3 Formulae Used in the Illustration Section of the Paper

In this section we show in a step-by-step fashion how the analytic results presented in the illustration section of the paper can be computed from the general formulae. The starting point is the vector of observed variables $\mathbf{V} = (V_1, V_2, V_3, V_4, V_5, V_6)^\intercal = (X_1, Y_1, X_2, Y_2, X_3, Y_3)^\intercal$, where we used the same ordering of the observed variables as

in Equation (S.4). In this ordering, variable X_2 is the third entry of the vector $\mathbf{V}$.

The joint distribution of observed variables $P(\mathbf{V})$ is multivariate normal with zero mean and the model-implied covariance matrix can be denoted in matrix notation as follows (Bollen, 1989):

$$\mathbf{\Sigma_V} = (\mathbf{I}_n - \mathbf{C})^{-1}\mathbf{\Psi}(\mathbf{I}_n - \mathbf{C})^{-\intercal} \tag{S.6}$$

Note that the entries of the model-implied covariance matrix are lengthy. Therefore, we only state those entries explicitly that are used for calculations later in this section:

$$\mathrm{V}(X_2) = c_{xx}^2\psi_{x_1x_1} + c_{xy}^2\psi_{y_1y_1} + 2c_{xx}c_{xy}\psi_{x_1y_1} + \psi_{xx} + 2c_{xx}\psi_{x_1x_2} \tag{S.7a}$$

$$\begin{aligned}\mathrm{V}(Y_3) = {} & (c_{xx}c_{yx} + c_{yx}c_{yy})^2\psi_{x_1x_1} + (c_{xy}c_{yx} + c_{yy}^2)^2\psi_{y_1y_1} \\ & + 2(c_{xx}c_{yx} + c_{yx}c_{yy})(c_{xy}c_{yx} + c_{yy}^2)\psi_{x_1y_1} \\ & + c_{yx}^2\psi_{xx} + (1 + c_{yy}^2)\psi_{yy} + 2c_{yx}c_{yy}\psi_{xy} \\ & + 2c_{yx}(c_{xx}c_{yx} + c_{yx}c_{yy})\psi_{x_1x_2} + 2c_{yy}(c_{xy}c_{yx} + c_{yy}^2)\psi_{y_1y_2} + 2c_{yy}\psi_{y_2y_3}\end{aligned} \tag{S.7b}$$

$$\begin{aligned}\mathrm{COV}(X_2, Y_3) = {} & c_{xx}(c_{xx}c_{yx} + c_{yx}c_{yy})\psi_{x_1x_1} + c_{xy}(c_{xy}c_{yx} + c_{yy}^2)\psi_{y_1y_1} \\ & + (c_{xy}(c_{xx}c_{yx} + c_{yx}c_{yy}) + c_{xx}(c_{xy}c_{yx} + c_{yy}^2))\psi_{x_1y_1} \\ & + c_{yx}\psi_{xx} + c_{yy}\psi_{xy} + (2c_{xx}c_{yx} + c_{yx}c_{yy})\psi_{x_1x_2} + c_{xy}c_{yy}\psi_{y_1y_2}\end{aligned} \tag{S.7c}$$

The marginal distribution $P(Y_3)$ can be obtained from the joint distribution $P(\mathbf{V})$ via marginalization (Rao, 1973). The marginal distribution $P(Y_3)$ is univariate normal with (i) zero mean and (ii) a variance equal to the $(6,6)$-th entry of the covariance matrix of $\mathbf{\Sigma}_V$ (recall that Y_3 is the 6-th entry in the vector of variables given the above ordering $(V_1, V_2, V_3, V_4, V_5, V_6)^\intercal := (X_1, Y_1, X_2, Y_2, X_3, Y_3)^\intercal$). Evaluating Equation (S.7b) at the population values of the parameters (see the first row of Table S.2) yields $\mathrm{V}(Y_3) = 766.91$ and consequently:

$$P(Y_3) = N_1(0\,,\,766.91) \tag{S.8}$$

The conditional distribution $P(Y_3 \mid X_2 = x_2)$ is univariate normal with (i) mean equal to the 5-th entry of the conditional mean vector $E(X_1, Y_1, Y_2, X_3, Y_3 \mid X_2 = x_2)$ and (ii) variance equal to the $(5,5)$-th entry of the conditional covariance matrix

$V(X_1, Y_1, Y_2, X_3, Y_3 \mid X_2 = x_2)$. Since the variables are joint normally distributed, the conditional moments can be obtained via a linear regression of Y_3 on X_2. Consequently, the conditional mean is given by:

$$\mathrm{E}(Y_3 \mid X_2 = x_2) = \frac{\mathrm{COV}(X_2, Y_3)}{\mathrm{V}(X_2)} x_2 = (c_{yx} + s) x_2 \tag{S.9}$$

Note that the conditional mean is additively decomposed into an causal component c_{yx} and a non-causal component s (the non-causal component is sometimes called spurious). The causal component of the conditional mean stems from the direct causal effect c_{yx} which quantifies the only causal path from X_2 to Y_3 in Figure 2 from the paper. The non-causal component s of the conditional mean stems from all non-causal paths from X_2 to Y_3 (e.g., $X_2 \leftarrow Y_1 \rightarrow Y_2 \rightarrow Y_3$, $X_2 \longleftrightarrow Y_2 \rightarrow Y_3$) and is equal to:

$$s = \frac{c_{xx} c_{yx} c_{yy} \psi_{x_1 x_1} + c_{xy} c_{yy}^2 \psi_{y_1 y_1} + (c_{xy} c_{yx} c_{yy} + c_{xx} c_{yy}^2) \psi_{x_1 y_1} + c_{yy} \psi_{xy} + c_{yx} c_{yy} \psi_{x_1 x_2} + c_{xy} c_{yy} \psi_{y_1 y_2}}{c_{xx}^2 \psi_{x_1 x_1} + c_{xy}^2 \psi_{y_1 y_1} + 2 c_{xx} c_{xy} \psi_{x_1 y_1} + \psi_{xx} + 2 c_{xx} \psi_{x_1 x_2}}$$

Similarly, the conditional variance of Y_3 given $X_2 = x_2$ can be obtained from the linear regression of Y_3 on X_2 and is given by the following formula:

$$\mathrm{V}(Y_3 \mid X_2 = x_2) = \mathrm{V}(Y_3) - \frac{\mathrm{COV}(X_2, Y_3)^2}{\mathrm{V}(X_2)} \tag{S.10}$$

The conditional variance $\mathrm{V}(Y_3 \mid X_2 = x_2)$ could be expressed as a function of the parameters $\boldsymbol{\theta}$ by plugging in Equations (S.7a), (S.7b), and (S.7c) into Equation (S.10). Since the resulting expression is lengthy and does not provide any immediate insights, we refrain from doing so. Evaluating expressions (S.9) and (S.10) at the population values of the parameters (see the first row of Table S.2), yields:

$$P(Y_3 \mid X_2 = x_2) = N_1(1.76 x_2 \,, 353.99) \tag{S.11a}$$

$$P(Y_3 \mid X_2 = 11.54) = N_1(1.76 \cdot 11.54 \,, 353.99) = N_1(20.31 \,, 353.99) \tag{S.11b}$$

The interventional distribution $P(Y_3 \mid do(x_2))$ can be calculated from Results 3 and 4 stated in the paper. We start with Equation (7a) from the paper, which states that the interventional distribution is given by:

$$\mathbf{V} \mid do(\mathbf{x}) \sim N_n^{n-K_x}(\, \mathbf{a}_1 \mathbf{x} \,,\, \mathbf{T}_1 \boldsymbol{\Psi} \mathbf{T}_1^\intercal \,) \tag{S.12}$$

The mean vector and the covariance matrix of the interventional distribution can be computed according to Equations (6a) and (6b) in the paper, respectively, which we restate here for the sake of completeness:

$$\mathrm{E}(\mathbf{V} \mid do(\mathbf{x})) = \mathbf{a}_1\mathbf{x} = (\mathbf{I}_n - \mathbf{I}_{\mathcal{N}}\mathbf{C})^{-1}\mathbf{1}_{\mathcal{I}}\mathbf{x} \tag{S.13a}$$

$$\mathrm{V}(\mathbf{V} \mid do(\mathbf{x})) = \mathbf{T}_1\boldsymbol{\Psi}\mathbf{T}_1^{\intercal} = (\mathbf{I}_n - \mathbf{I}_{\mathcal{N}}\mathbf{C})^{-1}\mathbf{I}_{\mathcal{N}}\boldsymbol{\Psi}\mathbf{I}_{\mathcal{N}}(\mathbf{I}_n - \mathbf{I}_{\mathcal{N}}\mathbf{C})^{-\intercal} \tag{S.13b}$$

The matrices $\mathbf{C}$ and $\boldsymbol{\Psi}$ are stated in Equation (S.4) and Equation (S.5), respectively. The zero-one matrices $\mathbf{I}_n$, $\mathbf{I}_{\mathcal{N}}$, $\mathbf{1}_{\mathcal{I}}$ are uniquely defined according to Definition 1 from the paper.

We are interested in the intervention $do(X_2 = 11.54)$, that is, we intervene on a single variable. Consequently, the set of interventional variables is given as $\mathcal{X} = \{X_2\}$ and the number of interventional variables is given by $K_x = 1$. Based on the ordering $\mathbf{V} = (X_1, Y_1, X_2, Y_2, X_3, Y_3)^{\intercal}$, the interventional variable is the third entry of the vector of observed variables and the set of interventional indexes is given by $\mathcal{I} = \{3\}$. Altogether, there are six variables in the system and consequently $n = 6$. The index set of non-interventional variables is defined as $\mathcal{N} = \{1, 2, 3, 4, 5, 6\} \setminus \{3\} = \{1, 2, 4, 5, 6\}$.

Note that the zero-one matrices in Definition 1 are uniquely determined by $n = 6$, $\mathcal{I} = \{3\}$, and $\mathcal{N} = \{1, 2, 3, 4, 5, 6\}$. $\mathbf{I}_n$ denotes the (6×6) identity matrix. $\mathbf{I}_{\mathcal{N}}$ is defined as a (6×6) diagonal matrix with zeros and ones as diagonal values. The i-th diagonal value is equal to one if $i \in \mathcal{N}$ and zero otherwise. $\mathbf{1}_{\mathcal{I}}$ collects all (6×1) unit vectors that correspond to the set of interventional indexes $\mathcal{I}$. In the running example, $\mathcal{I} = \{3\}$ and thus $\mathbf{1}_{\mathcal{I}}$ is equal to the third canonical unit vector of the $\mathbb{R}^6$.

$$n = 6,\ \mathcal{X} = \{X_2\},\ K_x = 1,\ \mathcal{I} = \{3\},\ \mathcal{N} = \{1, 2, 4, 5, 6\} \tag{S.14}$$

$$\mathbf{x} = x_2 = 11.54,\ \mathbf{1}_{\mathcal{I}} = \begin{pmatrix} 0 \\ 0 \\ 1 \\ 0 \\ 0 \\ 0 \end{pmatrix},\ \mathbf{I}_{\mathcal{N}} = \begin{pmatrix} 1 & 0 & 0 & 0 & 0 & 0 \\ 0 & 1 & 0 & 0 & 0 & 0 \\ 0 & 0 & 0 & 0 & 0 & 0 \\ 0 & 0 & 0 & 1 & 0 & 0 \\ 0 & 0 & 0 & 0 & 1 & 0 \\ 0 & 0 & 0 & 0 & 0 & 1 \end{pmatrix}$$

Plugging in $\mathbf{C}$ (from Equation [S.4]), $\boldsymbol{\Psi}$ (from Equation [S.5]), and the quantities stated in Equation (S.14) into the general formulae for the interventional mean as stated in Equation (S.13a) yields:

$$\mathrm{E}(\mathbf{V} \mid do(X_2 = 11.54)) = \mathrm{E}(\begin{pmatrix} X_1 \\ Y_1 \\ X_2 \\ Y_2 \\ X_3 \\ Y_3 \end{pmatrix} \mid do(X_2 = 11.54)) = \begin{pmatrix} 0 \\ 0 \\ 11.54 \\ 0 \\ c_{xx} \cdot 11.54 \\ c_{yx} \cdot 11.54 \end{pmatrix} = \begin{pmatrix} 0 \\ 0 \\ 11.54 \\ 0 \\ 0.577 \\ -6.924 \end{pmatrix} \tag{S.15}$$

The last equality sign is the numeric evaluation at the population values of the parameters (see the first row of Table S.2).

The numeric values of the interventional covariance matrix can be obtained by evaluation Equation (S.13b) analogously to the procedure demonstrated for the interventional means.

$$\mathrm{V}(\begin{pmatrix} X_1 \\ Y_1 \\ X_2 \\ Y_2 \\ X_3 \\ Y_3 \end{pmatrix} \mid do(X_2 = 11.54)) = \begin{pmatrix} 131.76 & 254.12 & 0 & 225.89 & 90.36 & 271.07 \\ 254.12 & 632.94 & 0 & 642.06 & 256.82 & 770.47 \\ 0.00 & 0.00 & 0 & 0.00 & 0.00 & 0.00 \\ 225.89 & 642.06 & 0 & 716.93 & 286.77 & 870.32 \\ 90.36 & 256.82 & 0 & 286.77 & 134.71 & 351.13 \\ 271.07 & 770.47 & 0 & 870.32 & 351.13 & 1096.39 \end{pmatrix} \tag{S.16}$$

Note that Equation (S.15) states the interventional mean both as a function of the parameters $\boldsymbol{\theta}$ and the corresponding numeric evaluation. By contrast, Equation (S.18) only contains the numeric evaluation due to space limitations. However, each entry of the interventional covariance matrix can be expressed as a function of the parameters

$\boldsymbol{\theta}$, as exemplified for the (6,6)th entry in the equation below.

$$\begin{aligned} \mathrm{V}(Y_3 \mid do(x_2)) = {} & c_{yx}^2 c_{yy}^2 \psi_{x_1x_1} + c_{yy}^4 \psi_{y_1y_1} + 2c_{yx}c_{yy}^3 \psi_{x_1y_1} \\ & + (1 + c_{yy}^2)\psi_{yy} + 2c_{yy}^3 \psi_{y_1y_2} + 2c_{yy}\psi_{y_2y_3} \end{aligned} \tag{S.17a}$$

The third row and the third column of the interventional covariance matrix in Equation (S.18) contains zeroes. The reason for this is, that the variable X_2 (third entry in $\mathbf{V} = (X_1, Y_1, X_2, Y_2, X_3, Y_3)^\intercal$) is no longer random given the intervention $do(X_2 = 11.54)$. To avoid working with singular matrices, one often focuses on the interventional distribution of all non-interventional variables. The vector of non-interventional variables is denotes as $\mathbf{V}_\mathcal{N}$ and is given by $\mathbf{V}_\mathcal{N} = (X_1, Y_1, Y_2, X_3, Y_3)^\intercal$ in the running example. The corresponding interventional covariance matrix is given by:

$$\mathrm{V}(\begin{pmatrix} X_1 \\ Y_1 \\ Y_2 \\ X_3 \\ Y_3 \end{pmatrix} \mid do(X_2 = 11.54)) = \begin{pmatrix} 131.76 & 254.12 & 225.89 & 90.36 & 271.07 \\ 254.12 & 632.94 & 642.06 & 256.82 & 770.47 \\ 225.89 & 642.06 & 716.93 & 286.77 & 870.32 \\ 90.36 & 256.82 & 286.77 & 134.71 & 351.13 \\ 271.07 & 770.47 & 870.32 & 351.13 & 1096.39 \end{pmatrix} \tag{S.18}$$

Following Result 4 from the paper, the resulting marginal interventional distribution $P(Y_3 \mid do(x_2))$ is univariate normal and can be obtained from the joint interventional distribution by selecting the 6th entry of the interventional mean and the (6,6)-th entry of the interventional covariance matrix. The general formulae from Result 4 is restated below for the sake of completeness:

$$P(\mathbf{y} \mid do(\mathbf{x})) \sim N_{K_y}\left(\mathbf{1}_{\mathcal{I}_y}^\intercal \mathbf{a}_1 \mathbf{x} \ , \ \mathbf{1}_{\mathcal{I}_y}^\intercal \mathbf{T}_1 \boldsymbol{\Psi} \mathbf{T}_1^\intercal \mathbf{1}_{\mathcal{I}_y} \right) \tag{S.19}$$

In the running example, the set of outcome variables is given by $\mathcal{Y} = \{Y_3\}$ and consequently $|\mathcal{Y}| = K_y = 1$. The index set of the outcome variables is $\mathcal{I}_y = \{6\}$ since Y_3 is the 6th entry in $\mathbf{V} = (X_1, Y_1, X_2, Y_2, X_3, Y_3)^\intercal$. The corresponding selection matrix $\mathbf{1}_{\mathcal{I}_y}$ is given by $(0, 0, 0, 0, 0, 1)^\intercal$. Evaluating Equation S.19 for these values yields following

marginal interventional distribution:

$$P(Y_3 \mid do(x_2)) = N_1(-.6x_2\,,\,1096.39) \tag{S.20}$$
$$P(Y_3 \mid do(11.54)) = N_1(-.6 \cdot 11.54\,,\,1096.39) = N_1(-6.92\,,\,1096.39)$$

Based on the result stated in Equation (S.20), namely that the marginal interventional distribution is univariate normal, the corresponding interventional probability density function (pdf) $f(y_3 \mid do(x_2))$ is given by:

$$f(y_3 \mid do(x_2)) = (2\pi V(Y_3 \mid do(x_2)))^{-\frac{1}{2}} \exp\left(-\frac{1}{2}\frac{(y_3 - E(Y_3 \mid do(x_2)))^2}{V(Y_3 \mid do(x_2))}\right) \tag{S.21}$$
$$=(2\pi)^{-\frac{1}{2}}(c_{yx}^2 c_{yy}^2 \psi_{x_1x_1} + c_{yy}^4 \psi_{y_1y_1} + 2c_{yx}c_{yy}^3\psi_{x_1y_1} + (1+c_{yy}^2)\psi_{yy} + 2c_{yy}^3\psi_{y_1y_2} + 2c_{yy}\psi_{y_2y_3})^{-\frac{1}{2}}$$
$$\times \exp\left(-\frac{1}{2}\frac{(y_3 - c_{yx}x_2)^2}{c_{yx}^2 c_{yy}^2 \psi_{x_1x_1} + c_{yy}^4 \psi_{y_1y_1} + 2c_{yx}c_{yy}^3\psi_{x_1y_1} + (1+c_{yy}^2)\psi_{yy} + 2c_{yy}^3\psi_{y_1y_2} + 2c_{yy}\psi_{y_2y_3}}\right)$$

Evaluating expression (S.21) at the population values of the parameters (see the first row of Table S.2) and $do(X_2 = 11.54)$, yields:

$$f(y_3 \mid do(x_2)) = (2\pi)^{-\frac{1}{2}}(1096.39)^{-\frac{1}{2}} \exp\left(-\frac{1}{2}\frac{(y_3 + 0.6x_2)^2}{1096.39}\right)$$
$$f(y_3 \mid do(11.54)) = (2\pi)^{-\frac{1}{2}}(1096.39)^{-\frac{1}{2}} \exp\left(-\frac{1}{2}\frac{(y_3 + 6.92)^2}{1096.39}\right) \tag{S.22}$$

The interventional probability $P(y^{low} \leq Y_3 \leq y^{up} \mid do(x_2))$ can be obtained via integration over the interventional pdf in Equation (S.21), yielding:

$$P(y^{\text{low}} \leq Y_3 \leq y^{up} \mid do(x_2)) = \int_{y^{low}}^{y^{up}} f(y_3 \mid do(x_2))\mathsf{d}y_3 = \int_{\frac{y^{low} - E(Y_3|do(x_2))}{\sqrt{V(Y_3|do(x_2))}}}^{\frac{y^{up} - E(Y_3|do(x_2))}{\sqrt{V(Y_3|do(x_2))}}} \phi(u)\mathsf{d}u$$
$$= \Phi\left(\frac{y^{up} - E(Y_3 \mid do(x_2))}{\sqrt{V(Y_3 \mid do(x_2))}}\right) - \Phi\left(\frac{y^{low} - E(Y_3 \mid do(x_2))}{\sqrt{V(Y_3 \mid do(x_2))}}\right) \tag{S.23}$$

The second equation sign follows from integration by substitution where ϕ and Φ denote the pdf and the cumulative distribution function of the standard normal distribution, respectively. Evaluating expression (S.23) at the values $y^{low} = -40$ and $y^{up} = 80$ and the population values of the parameters (see the first row of Table S.2), yields:

$$P(-40 \leq Y_3 \leq 80 \mid do(x_2)) = \Phi\left(\frac{80 + .6x_2}{1096.39}\right) - \Phi\left(\frac{-40 + .6x_2}{1096.39}\right) \tag{S.24a}$$

For the interventional level $do(X_2 = 11.54)$ we obtain:

$$P(-40 \leq Y_3 \leq 80 \mid do(11.54)) = \Phi\left(\frac{80 + 6.92}{1096.39}\right) - \Phi\left(\frac{-40 + 6.92}{1096.39}\right) = .8368 \quad \text{(S.24b)}$$

S.4 Structural Equations Given *do*-Type Interventions

In this section we show that the interventional equations (Equation [3] in the paper) provide an adequate description of the way the system of linear equations is modified by a *do*-type intervention. The starting point is a linear structural equation model as described in the section entitled "Graph-Based Causal Models with Linear Equations" of the paper. The following statements summarize some properties of the selection matrices introduced in Definition 1 of the paper.

1. *replace rows/columns by zeroes* Premultiplying (Postmultiplying) $\mathbf{C}$ by the matrix $\mathbf{I}_{\mathcal{N}}$: For all $i \in \mathcal{I}$ the entries of the i-th row (column) of $\mathbf{C}$ are replaced by zeroes while all other entries are left unchanged.

2. *select rows/columns* Premultiplying (Postmultiplying) $\mathbf{C}$ by the matrix $\mathbf{1}_{\mathcal{I}}^{\intercal}$ ($\mathbf{1}_{\mathcal{I}}$): selects all row (column) vectors of $\mathbf{C}$ with an interventional index $i \in \mathcal{I}$.

Thus, premultiplying $\mathbf{C}$ and $\boldsymbol{\varepsilon}$ by $\mathbf{I}_{\mathcal{N}}$ replaces all rows with an interventional index $i \in \mathcal{I}$ by rows of zeros while leaving all other rows unchanged. Thus, for the system of equations $\mathbf{V} = \mathbf{I}_{\mathcal{N}}\mathbf{C}\mathbf{V} + \mathbf{I}_{\mathcal{N}}\boldsymbol{\varepsilon}$ the i-th component on the right-hand side is equal to zero for all $i \in \mathcal{I}$. This accounts for the fact that, due to the intervention $do(\mathbf{x})$, the value of $V_i,\ i \in \mathcal{I}$, is exogenous and no longer determined by other variables V_j or the error term ε_i. However, the values of the interventional variables $\mathbf{V}_{\mathcal{I}}$ are not equal to zero but are set to the constant interventional levels $\mathbf{x}$. This is accounted for by adding $\mathbf{1}_{\mathcal{I}}\mathbf{x}$ on the right-hand side, yielding $\mathbf{V} = \mathbf{I}_{\mathcal{N}}\mathbf{C}\mathbf{V} + \mathbf{I}_{\mathcal{N}}\boldsymbol{\varepsilon} + \mathbf{1}_{\mathcal{I}}\mathbf{x}$, which is equal to the expression given in Equation (3) of the paper.

S.5 Proof of Local Identification

Let $\mathbf{V} = \mathbf{C}\mathbf{V} + \boldsymbol{\varepsilon}$ be a linear SEM as defined in Equation (2) of the paper. In the following, we focus on the linear SEM used in the illsutration section, where $n = 6$, and $\mathbf{C}$ and $\boldsymbol{\Psi}$ are given in Equation (S.4) and Equation (S.5), respectively. The proof of

local identification is based on a result from Bekker, Merckens, and Wansbeek (1994). To prepare our proof, we first reconcile the different notations used in the paper and in the book by Bekker et al. (1994). The latter use the following notation to denote a system of linear structural equations (see, e.g., Equation [3.2.1], page 47):

$$By + \Gamma x = \zeta \tag{S.25}$$

Where y is an $(m \times 1)$ random vector of endogenous variables, x is an $(k \times 1)$ random vector of exogenous variables, and ζ is an $(m \times 1)$ random vector of disturbances. The $(m \times m)$ matrix B and the $(m \times k)$ matrix Γ are real valued matrices of structural coefficients. The $(m \times m)$ covariance matrix of the disturbances is denoted as Σ_ζ. When translating the notation from Bekker et al. (1994) to the notation used throughout this paper, we set $k = 0$ and $\Gamma = \mathbf{0}$. In other words, we treat all *observed* variables as endogenous and Equation (S.25) simplifies accordingly:[2]

$$By = \zeta \tag{S.26}$$

A detailed comparison of the notation in Bekker et al. (1994) and the notation used throughout the paper suggests a translation as summarized in Table S.3.

Table S.3

Comparison of Notation

	notation used in the paper	notation used in Bekker et al. (1994)	relation
vector of observed variables	$\mathbf{V}$	y	$\mathbf{V} = y$
vector of error terms	$\boldsymbol{\varepsilon}$	ζ	$\boldsymbol{\varepsilon} = \zeta$
matrix of structural coefficients	$\mathbf{C}$	B	$(\mathbf{I} - \mathbf{C}) = B$
covariance matrix of error terms	$\boldsymbol{\Psi}$	Σ_ζ	$\boldsymbol{\Psi} = \Sigma_\zeta$
number of endogenous variables	n	m	$n = m$

Note. Table S.3 displays the notation used throughout the paper (column 1) and the notation used in Bekker et al. (1994) (column 2). Column 3 provides the relation between the symbols.

Following Equation (3.3.5) in Bekker et al. (1994), we denote the zero restrictions and the equality constraints imposed on the matrix of structural coefficients and the covariance

[2] Note that in the graph-based framework, *each* observed variable is affected by an unobserved error term (see Equation [S.4]) and consequently all observed variables are endogenous. This convention differs from other notations proposed in the literature (e.g., the LISREL notation).

matrix of error terms as follows:

$$\boldsymbol{\rho} = \begin{pmatrix} \boldsymbol{\rho}_I(\boldsymbol{\Psi}) \\ \boldsymbol{\rho}_{II}((\mathbf{I} - \mathbf{C})) \end{pmatrix} = \mathbf{0} \tag{S.27}$$

Starting with an unconstrained *symmetric* covariance matrix as stated below,

$$\begin{pmatrix} \psi_{11} & \psi_{12} & \psi_{13} & \psi_{14} & \psi_{15} & \psi_{16} \\ & \psi_{22} & \psi_{23} & \psi_{24} & \psi_{25} & \psi_{26} \\ & & \psi_{33} & \psi_{34} & \psi_{35} & \psi_{36} \\ & & & \psi_{44} & \psi_{45} & \psi_{46} \\ & & & & \psi_{55} & \psi_{56} \\ & & & & & \psi_{66} \end{pmatrix}$$

the zero restrictions and the equality constraints imposed on the covariance matrix of the error terms in Equation (S.5) are denoted by $\boldsymbol{\rho}_I(\boldsymbol{\Psi}) = \mathbf{0}$, where $\boldsymbol{\rho}_I$ is a vector-valued function with values in $\mathbb{R}^{r_I}$. In the example model, $r_I = 11$ and the explicit formula of the restriction function $\boldsymbol{\rho}_I$ is stated in Equation (S.30) at the end of this section. The upper eight entries of $\boldsymbol{\rho}_I(\boldsymbol{\Psi})$ correspond to zero restrictions, while the lower three entries of $\boldsymbol{\rho}_I(\boldsymbol{\Psi})$ reflect those equality constraints, which do *not* result from the symmetry of the covariance matrix.

Similarly, starting with an unconstrained matrix of structural coefficients (we use the identity matrix minus an unconstrained matrix of structural coefficients as displayed in Table S.3) as stated below,

$$\begin{pmatrix} 1-c_{11} & -c_{12} & -c_{13} & -c_{14} & -c_{15} & -c_{16} \\ -c_{21} & 1-c_{22} & -c_{23} & -c_{24} & -c_{25} & -c_{26} \\ -c_{31} & -c_{32} & 1-c_{33} & -c_{34} & -c_{35} & -c_{36} \\ -c_{41} & -c_{42} & -c_{43} & 1-c_{44} & -c_{45} & -c_{46} \\ -c_{51} & -c_{52} & -c_{53} & -c_{54} & 1-c_{55} & -c_{56} \\ -c_{61} & -c_{62} & -c_{63} & -c_{64} & -c_{65} & 1-c_{66} \end{pmatrix}$$

the zero restrictions and the equality constraints imposed on the matrix of structural coefficients in Equation (S.4) are denoted as $\boldsymbol{\rho}_{II}((\mathbf{I} - \mathbf{C})) = \mathbf{0}$, where $\boldsymbol{\rho}_{II}$ is a vector-

valued function with values in $\mathbb{R}^{r_{II}}$. In the example model, $r_{II} = 32$ and the explicit formula of the restriction function $\boldsymbol{\rho}_{II}$ is stated in Equation (S.30) at the end of this section. The upper 28 entries of $\boldsymbol{\rho}_{II}((\mathbf{I} - \mathbf{C})$ correspond to zero restrictions, while the lower four entries of $\boldsymbol{\rho}_{II}((\mathbf{I} - \mathbf{C})$ reflect equality constraints.

Stacking the restrictions together as in Equation (3.3.5) in Bekker et al. (1994) and calculating the Jacobian of the resulting stacked function $\boldsymbol{\rho}(\boldsymbol{\Psi}, (\mathbf{I} - \mathbf{C}))$ yields:

$$\boldsymbol{\rho}(\boldsymbol{\Psi}, (\mathbf{I} - \mathbf{C})) = \begin{pmatrix} \boldsymbol{\rho}_I(\boldsymbol{\Psi}) \\ \boldsymbol{\rho}_{II}((\mathbf{I} - \mathbf{C})) \end{pmatrix} \quad , \quad \frac{\partial \boldsymbol{\rho}}{\partial(\mathsf{vec}^\mathsf{T}((\mathbf{I} - \mathbf{C})^\mathsf{T}), \mathsf{vec}^\mathsf{T}(\boldsymbol{\Psi}))} = \begin{pmatrix} \mathbf{0} & \mathbf{R}_{\boldsymbol{\Psi}} \\ \mathbf{R}_{\mathbf{C}} & \mathbf{0} \end{pmatrix} \tag{S.28}$$

The (11×36) matrix $\mathbf{R}_{\boldsymbol{\Psi}}$ and the (32×36) matrix $\mathbf{R}_{\mathbf{C}}$ are stated explicitly in Equations (S.31) and (S.32), respectively. We are now in a position to use Theorem 3.3.1 from Bekker et al. (1994), which states the parameter vector $\boldsymbol{\theta}$ is locally identified, if, and only if, the Jacobian matrix $\tilde{\mathbf{J}}$ defined in Equation (S.29) has full column rank.

$$\tilde{\mathbf{J}} = \begin{pmatrix} \mathbf{R}_{\boldsymbol{\Psi}}(\mathbf{I}_{36} + \mathbf{K}_6)(\mathbf{I}_6 \otimes \boldsymbol{\Psi}) \\ \mathbf{R}_{\mathbf{C}}(\mathbf{I}_6 \otimes (\mathbf{I} - \mathbf{C})^\mathsf{T}) \end{pmatrix} \tag{S.29}$$

The rank evaluation of the $((32 + 11) \times 36)$ matrix $\tilde{\mathbf{J}}$ is performed using the computer algebra system Mathematica (Wolfram Research Inc., 2018). The corresponding code used to evaluate the rank of $\tilde{\mathbf{J}}$ is provided in Section S.7. The result obtained from Mathematica states that $\text{rank}(\tilde{\mathbf{J}}) = 36$, which completes the proof.

$$\boldsymbol{\rho}_I(\boldsymbol{\Psi}) = \begin{pmatrix} \psi_{14} \\ \psi_{15} \\ \psi_{16} \\ \psi_{23} \\ \psi_{25} \\ \psi_{26} \\ \psi_{36} \\ \psi_{45} \\ \psi_{33} - \psi_{55} \\ \psi_{34} - \psi_{56} \\ \psi_{44} - \psi_{66} \end{pmatrix} = \mathbf{0} \quad , \quad \boldsymbol{\rho}_{II}((\mathbf{I} - \mathbf{C})) = \begin{pmatrix} c_{11} - 1 \\ c_{12} \\ c_{13} \\ c_{14} \\ c_{15} \\ c_{16} \\ c_{21} \\ c_{22} - 1 \\ c_{23} \\ c_{24} \\ c_{25} \\ c_{26} \\ c_{33} - 1 \\ c_{34} \\ c_{35} \\ c_{36} \\ c_{43} \\ c_{44} - 1 \\ c_{45} \\ c_{46} \\ c_{51} \\ c_{52} \\ c_{55} - 1 \\ c_{56} \\ c_{61} \\ c_{62} \\ c_{65} \\ c_{66} - 1 \\ c_{31} - c_{53} \\ c_{32} - c_{54} \\ c_{41} - c_{63} \\ c_{42} - c_{64} \end{pmatrix} = \mathbf{0} \qquad \text{(S.30)}$$

$$
\mathbf{R}_{\boldsymbol{\Psi}} = \begin{pmatrix}
0 & 0 & 0 & 1 & 0 & 0 & 0 & 0 & 0 & 0 & 0 & 0 & 0 & 0 & 0 & 0 & 0 & 0 & 0 & 0 & 0 & 0 & 0 & 0 & 0 & 0 & 0 & 0 & 0 & 0 & 0 & 0 & 0 & 0 & 0 & 0 \\
0 & 0 & 0 & 0 & 1 & 0 & 0 & 0 & 0 & 0 & 0 & 0 & 0 & 0 & 0 & 0 & 0 & 0 & 0 & 0 & 0 & 0 & 0 & 0 & 0 & 0 & 0 & 0 & 0 & 0 & 0 & 0 & 0 & 0 & 0 & 0 \\
0 & 0 & 0 & 0 & 0 & 1 & 0 & 0 & 0 & 0 & 0 & 0 & 0 & 0 & 0 & 0 & 0 & 0 & 0 & 0 & 0 & 0 & 0 & 0 & 0 & 0 & 0 & 0 & 0 & 0 & 0 & 0 & 0 & 0 & 0 & 0 \\
0 & 0 & 0 & 0 & 0 & 0 & 0 & 0 & 1 & 0 & 0 & 0 & 0 & 0 & 0 & 0 & 0 & 0 & 0 & 0 & 0 & 0 & 0 & 0 & 0 & 0 & 0 & 0 & 0 & 0 & 0 & 0 & 0 & 0 & 0 & 0 \\
0 & 0 & 0 & 0 & 0 & 0 & 0 & 0 & 0 & 0 & 1 & 0 & 0 & 0 & 0 & 0 & 0 & 0 & 0 & 0 & 0 & 0 & 0 & 0 & 0 & 0 & 0 & 0 & 0 & 0 & 0 & 0 & 0 & 0 & 0 & 0 \\
0 & 0 & 0 & 0 & 0 & 0 & 0 & 0 & 0 & 0 & 0 & 1 & 0 & 0 & 0 & 0 & 0 & 0 & 0 & 0 & 0 & 0 & 0 & 0 & 0 & 0 & 0 & 0 & 0 & 0 & 0 & 0 & 0 & 0 & 0 & 0 \\
0 & 0 & 0 & 0 & 0 & 0 & 0 & 0 & 0 & 0 & 0 & 0 & 0 & 0 & 0 & 0 & 0 & 1 & 0 & 0 & 0 & 0 & 0 & 0 & 0 & 0 & 0 & 0 & 0 & 0 & 0 & 0 & 0 & 0 & 0 & 0 \\
0 & 0 & 0 & 0 & 0 & 0 & 0 & 0 & 0 & 0 & 0 & 0 & 0 & 0 & 0 & 0 & 0 & 0 & 0 & 0 & 0 & 0 & 1 & 0 & 0 & 0 & 0 & 0 & 0 & 0 & 0 & 0 & 0 & 0 & 0 & 0 \\
0 & 0 & 0 & 0 & 0 & 0 & 0 & 0 & 0 & 0 & 0 & 0 & 0 & 0 & 1 & 0 & 0 & 0 & 0 & 0 & 0 & 0 & 0 & 0 & 0 & 0 & 0 & 0 & -1 & 0 & 0 & 0 & 0 & 0 & 0 & 0 \\
0 & 0 & 0 & 0 & 0 & 0 & 0 & 0 & 0 & 0 & 0 & 0 & 0 & 0 & 0 & 0 & 0 & 0 & 0 & 0 & 0 & 1 & 0 & 0 & 0 & 0 & 0 & 0 & 0 & 0 & 0 & 0 & 0 & 0 & 0 & -1 \\
0 & 0 & 0 & 0 & 0 & 0 & 0 & 0 & 0 & 0 & 0 & 0 & 0 & 0 & 0 & 1 & 0 & 0 & 0 & 0 & 0 & 0 & 0 & 0 & 0 & 0 & 0 & 0 & 0 & -1 & 0 & 0 & 0 & 0 & 0 & 0
\end{pmatrix} \tag{S.31}
$$

$$
\mathbf{R_C} = \begin{pmatrix}
1 & 0 & 0 & 0 & 0 & 0 & 0 & 0 & 0 & 0 & 0 & 0 & 0 & 0 & 0 & 0 & 0 & 0 & 0 & 0 & 0 & 0 & 0 & 0 & 0 & 0 & 0 & 0 & 0 & 0 & 0 & 0 & 0 & 0 & 0 & 0 \\
0 & 1 & 0 & 0 & 0 & 0 & 0 & 0 & 0 & 0 & 0 & 0 & 0 & 0 & 0 & 0 & 0 & 0 & 0 & 0 & 0 & 0 & 0 & 0 & 0 & 0 & 0 & 0 & 0 & 0 & 0 & 0 & 0 & 0 & 0 & 0 \\
0 & 0 & 1 & 0 & 0 & 0 & 0 & 0 & 0 & 0 & 0 & 0 & 0 & 0 & 0 & 0 & 0 & 0 & 0 & 0 & 0 & 0 & 0 & 0 & 0 & 0 & 0 & 0 & 0 & 0 & 0 & 0 & 0 & 0 & 0 & 0 \\
0 & 0 & 0 & 1 & 0 & 0 & 0 & 0 & 0 & 0 & 0 & 0 & 0 & 0 & 0 & 0 & 0 & 0 & 0 & 0 & 0 & 0 & 0 & 0 & 0 & 0 & 0 & 0 & 0 & 0 & 0 & 0 & 0 & 0 & 0 & 0 \\
0 & 0 & 0 & 0 & 1 & 0 & 0 & 0 & 0 & 0 & 0 & 0 & 0 & 0 & 0 & 0 & 0 & 0 & 0 & 0 & 0 & 0 & 0 & 0 & 0 & 0 & 0 & 0 & 0 & 0 & 0 & 0 & 0 & 0 & 0 & 0 \\
0 & 0 & 0 & 0 & 0 & 1 & 0 & 0 & 0 & 0 & 0 & 0 & 0 & 0 & 0 & 0 & 0 & 0 & 0 & 0 & 0 & 0 & 0 & 0 & 0 & 0 & 0 & 0 & 0 & 0 & 0 & 0 & 0 & 0 & 0 & 0 \\
0 & 0 & 0 & 0 & 0 & 0 & 1 & 0 & 0 & 0 & 0 & 0 & 0 & 0 & 0 & 0 & 0 & 0 & 0 & 0 & 0 & 0 & 0 & 0 & 0 & 0 & 0 & 0 & 0 & 0 & 0 & 0 & 0 & 0 & 0 & 0 \\
0 & 0 & 0 & 0 & 0 & 0 & 0 & 1 & 0 & 0 & 0 & 0 & 0 & 0 & 0 & 0 & 0 & 0 & 0 & 0 & 0 & 0 & 0 & 0 & 0 & 0 & 0 & 0 & 0 & 0 & 0 & 0 & 0 & 0 & 0 & 0 \\
0 & 0 & 0 & 0 & 0 & 0 & 0 & 0 & 1 & 0 & 0 & 0 & 0 & 0 & 0 & 0 & 0 & 0 & 0 & 0 & 0 & 0 & 0 & 0 & 0 & 0 & 0 & 0 & 0 & 0 & 0 & 0 & 0 & 0 & 0 & 0 \\
0 & 0 & 0 & 0 & 0 & 0 & 0 & 0 & 0 & 1 & 0 & 0 & 0 & 0 & 0 & 0 & 0 & 0 & 0 & 0 & 0 & 0 & 0 & 0 & 0 & 0 & 0 & 0 & 0 & 0 & 0 & 0 & 0 & 0 & 0 & 0 \\
0 & 0 & 0 & 0 & 0 & 0 & 0 & 0 & 0 & 0 & 1 & 0 & 0 & 0 & 0 & 0 & 0 & 0 & 0 & 0 & 0 & 0 & 0 & 0 & 0 & 0 & 0 & 0 & 0 & 0 & 0 & 0 & 0 & 0 & 0 & 0 \\
0 & 0 & 0 & 0 & 0 & 0 & 0 & 0 & 0 & 0 & 0 & 1 & 0 & 0 & 0 & 0 & 0 & 0 & 0 & 0 & 0 & 0 & 0 & 0 & 0 & 0 & 0 & 0 & 0 & 0 & 0 & 0 & 0 & 0 & 0 & 0 \\
0 & 0 & 0 & 0 & 0 & 0 & 0 & 0 & 0 & 0 & 0 & 0 & 0 & 0 & 1 & 0 & 0 & 0 & 0 & 0 & 0 & 0 & 0 & 0 & 0 & 0 & 0 & 0 & 0 & 0 & 0 & 0 & 0 & 0 & 0 & 0 \\
0 & 0 & 0 & 0 & 0 & 0 & 0 & 0 & 0 & 0 & 0 & 0 & 0 & 0 & 0 & 1 & 0 & 0 & 0 & 0 & 0 & 0 & 0 & 0 & 0 & 0 & 0 & 0 & 0 & 0 & 0 & 0 & 0 & 0 & 0 & 0 \\
0 & 0 & 0 & 0 & 0 & 0 & 0 & 0 & 0 & 0 & 0 & 0 & 0 & 0 & 0 & 0 & 1 & 0 & 0 & 0 & 0 & 0 & 0 & 0 & 0 & 0 & 0 & 0 & 0 & 0 & 0 & 0 & 0 & 0 & 0 & 0 \\
0 & 0 & 0 & 0 & 0 & 0 & 0 & 0 & 0 & 0 & 0 & 0 & 0 & 0 & 0 & 0 & 0 & 1 & 0 & 0 & 0 & 0 & 0 & 0 & 0 & 0 & 0 & 0 & 0 & 0 & 0 & 0 & 0 & 0 & 0 & 0 \\
0 & 0 & 0 & 0 & 0 & 0 & 0 & 0 & 0 & 0 & 0 & 0 & 0 & 0 & 0 & 0 & 0 & 0 & 0 & 0 & 1 & 0 & 0 & 0 & 0 & 0 & 0 & 0 & 0 & 0 & 0 & 0 & 0 & 0 & 0 & 0 \\
0 & 0 & 0 & 0 & 0 & 0 & 0 & 0 & 0 & 0 & 0 & 0 & 0 & 0 & 0 & 0 & 0 & 0 & 0 & 0 & 0 & 1 & 0 & 0 & 0 & 0 & 0 & 0 & 0 & 0 & 0 & 0 & 0 & 0 & 0 & 0 \\
0 & 0 & 0 & 0 & 0 & 0 & 0 & 0 & 0 & 0 & 0 & 0 & 0 & 0 & 0 & 0 & 0 & 0 & 0 & 0 & 0 & 0 & 1 & 0 & 0 & 0 & 0 & 0 & 0 & 0 & 0 & 0 & 0 & 0 & 0 & 0 \\
0 & 0 & 0 & 0 & 0 & 0 & 0 & 0 & 0 & 0 & 0 & 0 & 0 & 0 & 0 & 0 & 0 & 0 & 0 & 0 & 0 & 0 & 0 & 1 & 0 & 0 & 0 & 0 & 0 & 0 & 0 & 0 & 0 & 0 & 0 & 0 \\
0 & 0 & 0 & 0 & 0 & 0 & 0 & 0 & 0 & 0 & 0 & 0 & 0 & 0 & 0 & 0 & 0 & 0 & 0 & 0 & 0 & 0 & 0 & 0 & 1 & 0 & 0 & 0 & 0 & 0 & 0 & 0 & 0 & 0 & 0 & 0 \\
0 & 0 & 0 & 0 & 0 & 0 & 0 & 0 & 0 & 0 & 0 & 0 & 0 & 0 & 0 & 0 & 0 & 0 & 0 & 0 & 0 & 0 & 0 & 0 & 0 & 1 & 0 & 0 & 0 & 0 & 0 & 0 & 0 & 0 & 0 & 0 \\
0 & 0 & 0 & 0 & 0 & 0 & 0 & 0 & 0 & 0 & 0 & 0 & 0 & 0 & 0 & 0 & 0 & 0 & 0 & 0 & 0 & 0 & 0 & 0 & 0 & 0 & 0 & 0 & 1 & 0 & 0 & 0 & 0 & 0 & 0 & 0 \\
0 & 0 & 0 & 0 & 0 & 0 & 0 & 0 & 0 & 0 & 0 & 0 & 0 & 0 & 0 & 0 & 0 & 0 & 0 & 0 & 0 & 0 & 0 & 0 & 0 & 0 & 0 & 0 & 0 & 1 & 0 & 0 & 0 & 0 & 0 & 0 \\
0 & 0 & 0 & 0 & 0 & 0 & 0 & 0 & 0 & 0 & 0 & 0 & 0 & 0 & 0 & 0 & 0 & 0 & 0 & 0 & 0 & 0 & 0 & 0 & 0 & 0 & 0 & 0 & 0 & 0 & 1 & 0 & 0 & 0 & 0 & 0 \\
0 & 0 & 0 & 0 & 0 & 0 & 0 & 0 & 0 & 0 & 0 & 0 & 0 & 0 & 0 & 0 & 0 & 0 & 0 & 0 & 0 & 0 & 0 & 0 & 0 & 0 & 0 & 0 & 0 & 0 & 0 & 1 & 0 & 0 & 0 & 0 \\
0 & 0 & 0 & 0 & 0 & 0 & 0 & 0 & 0 & 0 & 0 & 0 & 0 & 0 & 0 & 0 & 0 & 0 & 0 & 0 & 0 & 0 & 0 & 0 & 0 & 0 & 0 & 0 & 0 & 0 & 0 & 0 & 0 & 0 & 1 & 0 \\
0 & 0 & 0 & 0 & 0 & 0 & 0 & 0 & 0 & 0 & 0 & 0 & 0 & 0 & 0 & 0 & 0 & 0 & 0 & 0 & 0 & 0 & 0 & 0 & 0 & 0 & 0 & 0 & 0 & 0 & 0 & 0 & 0 & 0 & 0 & 1 \\
0 & 0 & 0 & 0 & 0 & 0 & 0 & 0 & 0 & 0 & 0 & 0 & 1 & 0 & 0 & 0 & 0 & 0 & 0 & 0 & 0 & 0 & 0 & 0 & 0 & 0 & -1 & 0 & 0 & 0 & 0 & 0 & 0 & 0 & 0 & 0 \\
0 & 0 & 0 & 0 & 0 & 0 & 0 & 0 & 0 & 0 & 0 & 0 & 0 & 1 & 0 & 0 & 0 & 0 & 0 & 0 & 0 & 0 & 0 & 0 & 0 & 0 & 0 & -1 & 0 & 0 & 0 & 0 & 0 & 0 & 0 & 0 \\
0 & 0 & 0 & 0 & 0 & 0 & 0 & 0 & 0 & 0 & 0 & 0 & 0 & 0 & 0 & 0 & 0 & 0 & 1 & 0 & 0 & 0 & 0 & 0 & 0 & 0 & 0 & 0 & 0 & 0 & 0 & 0 & -1 & 0 & 0 & 0 \\
0 & 0 & 0 & 0 & 0 & 0 & 0 & 0 & 0 & 0 & 0 & 0 & 0 & 0 & 0 & 0 & 0 & 0 & 0 & 1 & 0 & 0 & 0 & 0 & 0 & 0 & 0 & 0 & 0 & 0 & 0 & 0 & 0 & -1 & 0 & 0
\end{pmatrix} \tag{S.32}
$$

S.6 R Code

The calculations were done on Windows 10 Pro (64-bit), platform: x86_64-w64-mingw32/x64 (64-bit), with R-Studio (version 1.1.463), R (version 3.5.2), lavaan (version 0.6-3), vars (version 1.5-3), and matrixcalc (version 1.0-3). The following R script was used to create all reported numerical results in the main body of the paper and the supplementary material. Before running the script, please ensure that the packages vars, matrixcalc, and lavaan are installed on the device. Please note that at the time of publication, an R package with the tentative title "causalSEM" is being developed which will be made available via the Comprehensive R Archive Network (CRAN) shortly after the publication of the paper.

```
# Please note that at the time of publication, an R package with the
# tentative title ''causalSEM'' is being developed which will be made
# available via the Comprehensive R Archive Network (CRAN)
# shortly after the publication of the paper

library(vars)
library(matrixcalc)
library(lavaan)

# PART I: DATA GENERATION -------------------------------------------
# read in data and obtain estimates of a bivariate vector autoregression
# data is taken from Figure S.1 (lines with empty circles)
insulin <- c(17, 21, 25, 37, 43, 43, 43, 40, 57, 48, 45, 43, 28, 31, 29,
             24, 23, 23, 25, 24, 24, 22, 22, 21, 22, 23, 24, 26, 31, 18)
glucose <- c(65, 75, 85, 105, 115, 125, 120, 123, 118, 110, 100, 85, 80,
             75, 70, 75, 75, 70, 70, 73, 75, 80, 78, 75, 70, 70, 68, 65,
             63, 60)
d <- data.frame(insulin = (insulin - mean(insulin)),
                glucose = (glucose - mean(glucose)))
VAR1 <- VAR(d, p = 1, type = "const")
summary(VAR1) # output contains the values of Table S.1
B <- matrix(nrow = 2, c(0.05, 0.4, -0.6, 1.2), byrow = T)
print(abs(eigen(B)$values)) # check if regularity conditions are met

# population values as in the first row of Table S.2
# (see also: Table 1 of the paper)
theta_dgp <- c(0.05, 0.4, -0.6, 1.2, 131.76, 632.94, 254.12, 20, 40, 3,
               15, 2, 35, 10)

# specification of the data generating process (dgp)
model_dgp <- "

#error terms
ex1 =~ 1.0*x1
ex2 =~ 1.0*x2
ex3 =~ 1.0*x3
ey1 =~ 1.0*y1
ey2 =~ 1.0*y2
ey3 =~ 1.0*y3
ex1 ~~ 131.76*ex1
ey1 ~~ 632.94*ey1
ex2 ~~ 20*ex2
```

```
ey2 ~~ 40*ey2
ex3 ~~ 20*ex3
ey3 ~~ 40*ey3
ex2 ~~ 3*ey2
ex3 ~~ 3*ey3
ex1 ~~ 254.12*ey1
ex1 ~~ 15*ex2
ex2 ~~ 2*ex3
ey1 ~~ 35*ey2
ey2 ~~ 10*ey3

# regressions
x2~ 0.05*x1 + 0.4*y1
y2~ -0.6*x1 + 1.2*y1
x3~ 0.05*x2 + 0.4*y2
y3~ -0.6*x2 + 1.2*y2

# zero covariance restrictions
ex1~~0*ex3
ex1~~0*ey2
ex1~~0*ey3
ey1~~0*ex2
ey1~~0*ex3
ey1~~0*ey3
ex2~~0*ey3
ey2~~0*ex3
"
# specification of the model
model_sem<-"

#error terms
ex1 =~ 1.0*x1
ex2 =~ 1.0*x2
ex3 =~ 1.0*x3
ey1 =~ 1.0*y1
ey2 =~ 1.0*y2
ey3 =~ 1.0*y3
ex1 ~~ psix1x1*ex1
ey1 ~~ psiy1y1*ey1
ex2 ~~ psix*ex2
ey2 ~~ psiy*ey2
ex3 ~~ psix*ex3
ey3 ~~ psiy*ey3
ex2 ~~ psixy*ey2
ex3 ~~ psixy*ey3
ex1 ~~ psix1y1*ey1
ex1 ~~ psix1x2*ex2
ex2 ~~ psixx*ex3
ey1 ~~ psiy1y2*ey2
ey2 ~~ psiyy*ey3

# regressions
x2 ~ cxx*x1 + cxy*y1
y2 ~ cyx*x1 + cyy*y1
x3 ~ cxx*x2 + cxy*y2
y3 ~ cyx*x2 + cyy*y2

# zero covariance restrictions
ex1 ~~ 0*ex3
ex1 ~~ 0*ey2
ex1 ~~ 0*ey3
```

```
ey1 ~~ 0*ex2
ey1 ~~ 0*ex3
ey1 ~~ 0*ey3
ex2 ~~ 0*ey3
ey2 ~~ 0*ex3
"
# sample N = 100 individuals from the population and obtain estimates
set.seed(34995)
d_100 <- simulateData(model_dgp, sample.nobs = 100, seed = 45903)
d_100 <- d_100[, c(1, 4, 2, 5, 3, 6)]
fit_100 <- lavaan(model_sem, data = d_100)

# estimated values as in the second row of Table S.2
# (see also: Table 1 of the paper)
# (sample quantities are marked with _s)

theta_s <- coef(fit_100)[c("cxx", "cxy", "cyx", "cyy", "psix1x1", "psiy1y1",
                        "psix1y1", "psix", "psiy", "psixy", "psix1x2",
                        "psixx", "psiy1y2", "psiyy")]

# further numeric values used in the main paper
x2 <- 11.54 # interventional level
ylow <- -40 # lower bound of outcome range
yup <- 80 # upper bound of outcome range

# PART II: RESULTS ON THE POPULATION LEVEL ------------
# FROM THE ILLUSTRATION SECTION -----------------------

n <- 6 # number of observed variables

# covariance matrix of error terms as stated in Equation S.5
# (see also: Equation 20 of the paper)
Psi <- matrix(nrow = n, ncol = n, 0)
Psi[1,1] <- theta_dgp[5]
Psi[3,3] <- theta_dgp[8]
Psi[5,5] <- theta_dgp[8]
Psi[2,2] <- theta_dgp[6]
Psi[4,4] <- theta_dgp[9]
Psi[6,6] <- theta_dgp[9]
Psi[1,2] <- theta_dgp[7]
Psi[2,1] <- theta_dgp[7]
Psi[1,3] <- theta_dgp[11]
Psi[3,1] <- theta_dgp[11]
Psi[2,4] <- theta_dgp[13]
Psi[4,2] <- theta_dgp[13]
Psi[3,5] <- theta_dgp[12]
Psi[5,3] <- theta_dgp[12]
Psi[4,6] <- theta_dgp[14]
Psi[6,4] <- theta_dgp[14]
Psi[3,4] <- theta_dgp[10]
Psi[4,3] <- theta_dgp[10]
Psi[5,6] <- theta_dgp[10]
Psi[6,5] <- theta_dgp[10]

is.positive.definite(Psi) # check if matrix is positive definite

# matrix of structural coefficients as stated in Equation S.4
# (see also Equation 19 of the paper)
C <- matrix(nrow = n, ncol = n, 0)
C[3,1] <- theta_dgp[1]
C[3,2] <- theta_dgp[2]
```

```
C[4,1] <- theta_dgp[3]
C[4,2] <- theta_dgp[4]
C[5,3] <- theta_dgp[1]
C[5,4] <- theta_dgp[2]
C[6,3] <- theta_dgp[3]
C[6,4] <- theta_dgp[4]

# expected values of error terms are zero
E_epsilon <- rep(0, n)

# model implied mean vector of the joint distribution
# of observed variables (see Equation 2 of the paper)
I_Cinv <- solve(diag(1, nrow = n) - C)
E_V <- I_Cinv %*% E_epsilon

# model implied covariance matrix of the joint distribution of
# observed variables as stated in Equation S.6
# (see also: Equation 2 of the paper)
Sigma_V <- I_Cinv %*% Psi %*% t(I_Cinv)

# variances and covariances as stated in Equations S.7a, S.7b, and S.7c
V_X2 <- Sigma_V[3,3]
V_Y3 <- Sigma_V[6,6]
COV_X2V3 <- Sigma_V[3,6]

# conditional variance of Y3 given X2=11.54
# as stated in Equation S.9
E_Y3condx2 <- COV_X2V3 / V_X2 * x2

# conditional variance of Y3 given X2=11.54
# as stated in Equation S.10
V_Y3condx2 <- V_Y3 - ((COV_X2V3 ^ 2) / V_X2)

# interventional mean and covariance matrix for do(x2)

# zero-one matrices according to Definition 1 of the paper
# (see also Equation 21 of the paper and Equation S.14)
ONE_I <- c(0, 0, 1, 0, 0, 0) # select interventional variables
IN <- diag(c(1, 1, 0, 1, 1, 1))

# matrices of the linear transformation as stated
# in Equation 4 of the paper
T1 <- solve(diag(1, nrow = n)-IN %*% C) %*% IN
a1 <- solve(diag(1, nrow =n )-IN %*% C) %*% ONE_I

# interventional mean as stated in Equation 6a of the paper
# (see also Equation S.13a)
E_dox2 <- a1*x2

# interventional variance as stated in Equation 6b of the paper
# (see also Equation S.13b)
V_dox2 <- T1 %*% Psi %*% t(T1)

# causal quantity gamma2 as defined in Equation 13 of the paper
# as the half-vectorized interventional covariance matrix
V_dox2_vech <- lav_matrix_vech(V_dox2)

# unconditional, conditional and interventional probabilities
# as plotted in Figure 7 of the paper
P_Y3unconditional <- (pnorm(yup, mean = E_V[6], sd = sqrt(Sigma_V[6,6])) -
                    pnorm(ylow, mean = E_V[6], sd = sqrt(Sigma_V[6,6])))
```

```
P_Y3dox2 <- (pnorm(yup, mean = E_dox2[6], sd = sqrt(V_dox2[6,6])) -
            pnorm(ylow, mean = E_dox2[6], sd = sqrt(V_dox2[6,6])))
P_Y3condX2 <- (pnorm(yup, mean = E_Y3condx2, sd = sqrt(V_Y3condx2)) -
              pnorm(ylow, mean = E_Y3condx2, sd = sqrt(V_Y3condx2)))

# unconditional, conditional and interventional intervall forecasts as stated
# in the subsection "Interventional Distribution vs. Conditional Distribution"
uncond_low <- E_V[6] - qnorm(0.975) * sqrt(Sigma_V[6,6])
uncond_up <- E_V[6] + qnorm(0.975) * sqrt(Sigma_V[6,6])
cond_low <- E_Y3condx2 - qnorm(0.975) * sqrt(V_Y3condx2)
cond_up <- E_Y3condx2 + qnorm(0.975) * sqrt(V_Y3condx2)
int_low <- E_dox2[6] - qnorm(0.975) * sqrt(V_dox2[6,6])
int_up <- E_dox2[6] + qnorm(0.975) * sqrt(V_dox2[6,6])

# interventional probability density function f(y3|do(X2=11.54))
# evaluated at y3=0
y3 <- 0
f_y3_dox2 <- dnorm(y3, mean = E_dox2[6], sd = sqrt(V_dox2[6, 6]))

# interventional probability P(-40 < Y3 < 80 | do(X2=11.54))
P_y3_dox2 <- pnorm(80, mean = E_dox2[6], sd = sqrt(V_dox2[6, 6])) -
            pnorm(-40, mean = E_dox2[6], sd = sqrt(V_dox2[6,6]))

# PART III: ESTIMATION RESULTS -----------------------
# FROM THE ILLUSTRATION SECTION -----------------------
# sample quantities are marked with _s ---------------

# extract the asymptotic covariance matrix
AV_theta_s <- vcov(fit_100)

# reorder the entries of the asymptotic covariance matrix
AV_theta_s_r <- AV_theta_s[c("cxx", "cxy", "cyx", "cyy", "psix1x1",
                           "psiy1y1", "psix1y1", "psix", "psiy", "psixy",
                           "psix1x2","psixx", "psiy1y2", "psiyy"), ]
AV_theta_s <- AV_theta_s_r[, c("cxx", "cxy", "cyx", "cyy", "psix1x1",
                             "psiy1y1", "psix1y1", "psix", "psiy", "psixy",
                             "psix1x2", "psixx", "psiy1y2", "psiyy")]

# asymptotic standard errors as displayed in Table 1
ASE_theta_s <- sqrt(diag(AV_theta_s))

# approximate z-values as displayed in Table 1
# which are computed based on rounded values
z_theta_rounded_s <- (round(round(theta_s, 2) / round(ASE_theta_s, 2), 2))

# estimated covariance matrix of error terms
# (sample quantities are marked with _s)
Psi_s <- matrix(nrow = n, ncol = n, 0)
Psi_s[1,1] <- theta_s[5]
Psi_s[3,3] <- theta_s[8]
Psi_s[5,5] <- theta_s[8]
Psi_s[2,2] <- theta_s[6]
Psi_s[4,4] <- theta_s[9]
Psi_s[6,6] <- theta_s[9]
Psi_s[1,2] <- theta_s[7]
Psi_s[2,1] <- theta_s[7]
Psi_s[1,3] <- theta_s[11]
Psi_s[3,1] <- theta_s[11]
Psi_s[2,4] <- theta_s[13]
Psi_s[4,2] <- theta_s[13]
Psi_s[3,5] <- theta_s[12]
```

```
Psi_s[5,3] <- theta_s[12]
Psi_s[4,6] <- theta_s[14]
Psi_s[6,4] <- theta_s[14]
Psi_s[3,4] <- theta_s[10]
Psi_s[4,3] <- theta_s[10]
Psi_s[5,6] <- theta_s[10]
Psi_s[6,5] <- theta_s[10]

# check if matrix is positive definite
is.positive.definite(Psi_s)

# matrix structural coefficients
# (sample quantities are marked with _s)
C_s <- matrix(nrow = n, ncol = n, 0)
C_s[3,1] <- theta_s[1]
C_s[3,2] <- theta_s[2]
C_s[4,1] <- theta_s[3]
C_s[4,2] <- theta_s[4]
C_s[5,3] <- theta_s[1]
C_s[5,4] <- theta_s[2]
C_s[6,3] <- theta_s[3]
C_s[6,4] <- theta_s[4]

# model implied covariance matrix of the joint distribution of
# observed variables as stated in Equation S.6
# see also: Equation 2 of the paper; sample quantities are marked with _s
I_Cinv_s <- solve(diag(1, nrow = n) - C_s)
Sigma_V_s <- I_Cinv_s %*% Psi_s %*% t(I_Cinv_s)

# matrices of the linear transformation as stated
# in Equation 4 of the paper (sample quantities are marked with _s)
T1_s <- solve(diag(1, nrow = n) - IN %*% C_s) %*% IN
a1_s <- solve(diag(1, nrow = n) - IN %*% C_s) %*% ONE_I

# interventional mean as stated in Equation 6a of the paper
# (see also Equation S.13a) (sample quantities are marked with _s)
E_dox2_s <- a1_s * x2

# interventional variance as stated in Equation 6b of the paper
# (see also Equation S.13b) (sample quantities are marked with _s)
V_dox2_s <- T1_s %*% Psi_s %*% t(T1_s)

# causal quantity gamma2 as defined in Equation 13 of the paper
# as the half-vectorized interventional covariance matrix
V_dox2_vech_s <- lav_matrix_vech(V_dox2_s)

# AV of the estimator of gamma1 according to Equation 17 --------------
# gamma1 is the interventional mean vector ----------------------------
# as defined in Equation 12a of the paper -----------------------------

# first factor of the Kronecker product in the Jacobian in Equation 18a
F11_s <- x2 * t(ONE_I ) %*% t(I_Cinv_s)

# second factor of the Kronecker product in the Jacobian
F12_s <- I_Cinv_s %*% IN

# entire Kronecker product inside the bracket in Equation 18a
F1_s <- kronecker(F11_s, F12_s, FUN = "*", make.dimnames = FALSE)

# Jacobian of C(theta) with respect to theta
D_C_theta <- matrix(c(0, 0, 0, 0, 0, 0, 0, 0, 0, 0, 0, 0, 0, 0,
```

```
                    0, 0, 0, 0, 0, 0, 0, 0, 0, 0, 0, 0, 0, 0,
                    1, 0, 0, 0, 0, 0, 0, 0, 0, 0, 0, 0, 0, 0,
                    0, 0, 1, 0, 0, 0, 0, 0, 0, 0, 0, 0, 0, 0,
                    0, 0, 0, 0, 0, 0, 0, 0, 0, 0, 0, 0, 0, 0,
                    0, 0, 0, 0, 0, 0, 0, 0, 0, 0, 0, 0, 0, 0,
                    0, 0, 0, 0, 0, 0, 0, 0, 0, 0, 0, 0, 0, 0,
                    0, 0, 0, 0, 0, 0, 0, 0, 0, 0, 0, 0, 0, 0,
                    0, 1, 0, 0, 0, 0, 0, 0, 0, 0, 0, 0, 0, 0,
                    0, 0, 0, 1, 0, 0, 0, 0, 0, 0, 0, 0, 0, 0,
                    0, 0, 0, 0, 0, 0, 0, 0, 0, 0, 0, 0, 0, 0,
                    0, 0, 0, 0, 0, 0, 0, 0, 0, 0, 0, 0, 0, 0,
                    0, 0, 0, 0, 0, 0, 0, 0, 0, 0, 0, 0, 0, 0,
                    0, 0, 0, 0, 0, 0, 0, 0, 0, 0, 0, 0, 0, 0,
                    0, 0, 0, 0, 0, 0, 0, 0, 0, 0, 0, 0, 0, 0,
                    0, 0, 0, 0, 0, 0, 0, 0, 0, 0, 0, 0, 0, 0,
                    1, 0, 0, 0, 0, 0, 0, 0, 0, 0, 0, 0, 0, 0,
                    0, 0, 1, 0, 0, 0, 0, 0, 0, 0, 0, 0, 0, 0,
                    0, 0, 0, 0, 0, 0, 0, 0, 0, 0, 0, 0, 0, 0,
                    0, 0, 0, 0, 0, 0, 0, 0, 0, 0, 0, 0, 0, 0,
                    0, 0, 0, 0, 0, 0, 0, 0, 0, 0, 0, 0, 0, 0,
                    0, 0, 0, 0, 0, 0, 0, 0, 0, 0, 0, 0, 0, 0,
                    0, 1, 0, 0, 0, 0, 0, 0, 0, 0, 0, 0, 0, 0,
                    0, 0, 0, 1, 0, 0, 0, 0, 0, 0, 0, 0, 0, 0,
                    0, 0, 0, 0, 0, 0, 0, 0, 0, 0, 0, 0, 0, 0,
                    0, 0, 0, 0, 0, 0, 0, 0, 0, 0, 0, 0, 0, 0,
                    0, 0, 0, 0, 0, 0, 0, 0, 0, 0, 0, 0, 0, 0,
                    0, 0, 0, 0, 0, 0, 0, 0, 0, 0, 0, 0, 0, 0,
                    0, 0, 0, 0, 0, 0, 0, 0, 0, 0, 0, 0, 0, 0,
                    0, 0, 0, 0, 0, 0, 0, 0, 0, 0, 0, 0, 0, 0,
                    0, 0, 0, 0, 0, 0, 0, 0, 0, 0, 0, 0, 0, 0,
                    0, 0, 0, 0, 0, 0, 0, 0, 0, 0, 0, 0, 0, 0,
                    0, 0, 0, 0, 0, 0, 0, 0, 0, 0, 0, 0, 0, 0,
                    0, 0, 0, 0, 0, 0, 0, 0, 0, 0, 0, 0, 0, 0,
                    0, 0, 0, 0, 0, 0, 0, 0, 0, 0, 0, 0, 0, 0,
                    0, 0, 0, 0, 0, 0, 0, 0, 0, 0, 0, 0, 0, 0)
                  , ncol = 14, nrow = 36, byrow = T)

# Jacobian of g1 with respect to theta as stated in Equation 18a
D_Edox2_s <- F1_s %*% D_C_theta

# AV of estimator of the interventional mean according to Equation 17
AV_gamma1_s <- D_Edox2_s %*% AV_theta_s %*% t(D_Edox2_s)

# ASE of the estimate of E(Y3|do(X2=11.54)) as in Table 2
ASE_gamma1_s <- sqrt(AV_gamma1_s[6, 6])

# approximate z-values as displayed in Table 2
# which are computed based on rounded values
z_gamma1_rounded_s <- round(round(E_dox2_s[6], 4) / round(ASE_gamma1_s, 4),4)

# AV of the estimator of gamma2 according to Equation 17 -------------
# gamma2 is the half-vectorized interventional covariance matrix -----
# as defined in Equation 13 of the paper ---------------------------

# define zero-one matrices
I6 <- diag(1, nrow = n) # identity matrix
K6 <- commutation.matrix(n, n) # commutation matrix

# first factor of the matrix product in G2C in Corollary 11
G2C_1_s <- (diag(1, nrow = n ^ 2) + K6)
```

```
# second factor of the matrix product in G2C in Corollary 11
G2C_2_s <- kronecker(T1_s %*% Psi_s %*% IN, I6, FUN = "*",
                   make.dimnames = FALSE)

# third factor of the matrix product in G2C in Corollary 11
I_Cdox2_inv_s <- solve(diag(1, nrow = n)-IN %*% C_s)
G2C_3_s <- kronecker(t(I_Cdox2_inv_s), T1_s, FUN = "*",
                   make.dimnames = FALSE)

# G2C in Corollary 11
G2C_s <- G2C_1_s %*% G2C_2_s %*% G2C_3_s

# first factor of the matrix product in G2Psi in Corollary 11
G2Psi_1_s <- kronecker(I_Cdox2_inv_s, I_Cdox2_inv_s, FUN = "*",
                     make.dimnames = FALSE)

# second factor of the matrix product in G2Psi in Corollary 11
G2Psi_2_s <- kronecker(IN, IN, FUN = "*", make.dimnames = FALSE)

# G2Psi in Corollary 11
G2Psi_s <- G2Psi_1_s %*% G2Psi_2_s

# Jacobian of Psi(theta) with respect to theta; we use the derivative of
# vec(PSI(theta)) and NOT the HALF-vectorized version
D_Psi_theta <- matrix(
  c(0, 0, 0, 0, 1, 0, 0, 0, 0, 0, 0, 0, 0, 0,
    0, 0, 0, 0, 0, 0, 1, 0, 0, 0, 0, 0, 0, 0,
    0, 0, 0, 0, 0, 0, 0, 0, 0, 0, 1, 0, 0, 0,
    0, 0, 0, 0, 0, 0, 0, 0, 0, 0, 0, 0, 0, 0,
    0, 0, 0, 0, 0, 0, 0, 0, 0, 0, 0, 0, 0, 0,
    0, 0, 0, 0, 0, 0, 0, 0, 0, 0, 0, 0, 0, 0,
    0, 0, 0, 0, 0, 0, 1, 0, 0, 0, 0, 0, 0, 0,
    0, 0, 0, 0, 0, 1, 0, 0, 0, 0, 0, 0, 0, 0,
    0, 0, 0, 0, 0, 0, 0, 0, 0, 0, 0, 0, 0, 0,
    0, 0, 0, 0, 0, 0, 0, 0, 0, 0, 0, 0, 1, 0,
    0, 0, 0, 0, 0, 0, 0, 0, 0, 0, 0, 0, 0, 0,
    0, 0, 0, 0, 0, 0, 0, 0, 0, 0, 0, 0, 0, 0,
    0, 0, 0, 0, 0, 0, 0, 0, 0, 0, 1, 0, 0, 0,
    0, 0, 0, 0, 0, 0, 0, 0, 0, 0, 0, 0, 0, 0,
    0, 0, 0, 0, 0, 0, 0, 1, 0, 0, 0, 0, 0, 0,
    0, 0, 0, 0, 0, 0, 0, 0, 0, 1, 0, 0, 0, 0,
    0, 0, 0, 0, 0, 0, 0, 0, 0, 0, 0, 1, 0, 0,
    0, 0, 0, 0, 0, 0, 0, 0, 0, 0, 0, 0, 0, 0,
    0, 0, 0, 0, 0, 0, 0, 0, 0, 0, 0, 0, 0, 0,
    0, 0, 0, 0, 0, 0, 0, 0, 0, 0, 0, 0, 1, 0,
    0, 0, 0, 0, 0, 0, 0, 0, 0, 1, 0, 0, 0, 0,
    0, 0, 0, 0, 0, 0, 0, 0, 1, 0, 0, 0, 0, 0,
    0, 0, 0, 0, 0, 0, 0, 0, 0, 0, 0, 0, 0, 0,
    0, 0, 0, 0, 0, 0, 0, 0, 0, 0, 0, 0, 0, 1,
    0, 0, 0, 0, 0, 0, 0, 0, 0, 0, 0, 0, 0, 0,
    0, 0, 0, 0, 0, 0, 0, 0, 0, 0, 0, 0, 0, 0,
    0, 0, 0, 0, 0, 0, 0, 0, 0, 0, 0, 1, 0, 0,
    0, 0, 0, 0, 0, 0, 0, 0, 0, 0, 0, 0, 0, 0,
    0, 0, 0, 0, 0, 0, 0, 1, 0, 0, 0, 0, 0, 0,
    0, 0, 0, 0, 0, 0, 0, 0, 0, 1, 0, 0, 0, 0,
    0, 0, 0, 0, 0, 0, 0, 0, 0, 0, 0, 0, 0, 0,
    0, 0, 0, 0, 0, 0, 0, 0, 0, 0, 0, 0, 0, 0,
    0, 0, 0, 0, 0, 0, 0, 0, 0, 0, 0, 0, 0, 0,
    0, 0, 0, 0, 0, 0, 0, 0, 0, 0, 0, 0, 0, 1,
    0, 0, 0, 0, 0, 0, 0, 0, 0, 1, 0, 0, 0, 0,
    0, 0, 0, 0, 0, 0, 0, 0, 1, 0, 0, 0, 0, 0)
```

```
  , ncol = 14, nrow = 36, byrow = T)

# Jacobian of g2 with respect to theta as stated in Equation 18b
# note that gamma2 is defined as the HALF-vectorized interventional
# covariance matrix in Equation 13
D_n <- matrixcalc::elimination.matrix(n = n)
D_Vdox2_s <- D_n %*% (G2Psi_s %*% D_Psi_theta + G2C_s %*% D_C_theta)

# AV of estimator of the half-vectorized interventional
# covariance matrix according to Equation 17
AV_gamma2_s <- D_Vdox2_s %*% AV_theta_s %*% t(D_Vdox2_s)

# ASE of the estimate of V(Y3|do(X2=11.54)) as in Table 2
# note that the half-vectorized 6x6 covariance matrix
# has (6*(6+1))/2=21 entries
ASE_gamma2_s <- sqrt(AV_gamma2_s[21,21])

# approximate z-values as of V(Y3|do(X2=11.54)) displayed in Table 2
# Note: values are rounded and computed based on rounded values;
# half-vectorized 6x6 covariance matrix has (6*(6+1))/2=21 entries
z_gamma2_rounded_s <- round(round(V_dox2_vech_s[21], 4) /
                         round(ASE_gamma2_s, 4), 4)

# AV of the estimator of gamma3 according to Equation 17 ---------------
# gamma3 is the interventional probability density function (pdf) ------
# as defined in Equation 14 of the paper -----------------------------
# NOTE: caluculations in this section of the R-script are performed for
# the univariate outcome y3 given the intervention do(X2=11.54)

# we evaluate f(y3|do(X2=11.54)) at y3=0
f_y3_dox2_s <- dnorm(y3, mean = E_dox2_s[6], sd = sqrt(V_dox2_s[6, 6]))

# G3mu in Corollary 11 (here: we have univariate outcome Y3)
G3mu_s <- (y3 - E_dox2_s[6]) * (V_dox2_s[6, 6]) ^ (-1)

# G3SIGMA in Corollary 11 (here: we have univariate outcome Y3)
G3SIGMA_s <- 1 / 2 * ((y3 - E_dox2_s[6]) ^ 2 * V_dox2_s[6, 6] ^ (-2) -
                     V_dox2_s[6, 6] ^ (-1))

# first component of rightmost vector in Equation 18c
D_Edox2_Y3_s <- D_Edox2_s[6,]

# second component of rightmost vector in Equation 18c
D_Vdox2_Y3_s <- D_Vdox2_s[21,]

# Jacobian of g3 with respect to theta as stated in Equation 18c
D_f_y3_dox2_s <- f_y3_dox2_s * matrix(c(G3mu_s, G3SIGMA_s), nrow = 1) %*%
               matrix(c(D_Edox2_Y3_s, D_Vdox2_Y3_s), nrow = 2, byrow = TRUE)

# AV of estimator of the interventional pdf according to Equation 17
AV_gamma3_s <- D_f_y3_dox2_s %*% AV_theta_s %*% t(D_f_y3_dox2_s)

# ASE of of the estimate of f(Y3|do(X2=11.54)) as in Table 2
ASE_gamma3_s <- sqrt(AV_gamma3_s)

# approximate z-values as displayed in Table 2
# which are computed based on rounded values
z_gamma3_rounded_s <- round(round(f_y3_dox2_s, 4) /
                    round(sqrt(AV_gamma3_s), 4), 4)

# AV of the estimator of gamma4 according to Equation 17 ---------------
```

```
# gamma4 is the interventional probability ----------------------------
# as defined in Equation 15 of the paper ------------------------------
# NOTE: caluculations in this section of the R-script are performed for
# the univariate outcome y3 given the intervention do(X2=11.54)

# gamma4 is the univariate interventional probability
# P(-40 < Y3 < 80 | do(X2=11.54))
P_y3_dox2_s <- pnorm(80, mean = E_dox2_s[6], sd = sqrt(V_dox2_s[6, 6])) -
             pnorm(-40, mean = E_dox2_s[6], sd = sqrt(V_dox2_s[6,6]))

# G4mu in Corollary 11
densup_s <- dnorm(yup , mean = E_dox2_s[6], sd = sqrt(V_dox2_s[6,6]))
denslow_s <- dnorm(ylow , mean = E_dox2_s[6], sd = sqrt(V_dox2_s[6,6]))
G4mu_s <- - 1 / sqrt(V_dox2_s[6,6]) * (densup_s - denslow_s)

# G4sigma in Corollary 11
zlow_s <- (ylow - E_dox2_s[6]) / sqrt(V_dox2_s[6, 6])
zup_s <- (yup - E_dox2_s[6]) / sqrt(V_dox2_s[6, 6])
G4sigma_s <- - 1 / (2 * (sqrt(V_dox2_s[6, 6])) ^ 2) *
            (densup_s * zup_s - denslow_s * zlow_s)

# Jacobian of g4 with respect to theta as stated in Equation 18d
D_P_y3_dox2_s <- matrix(c(G4mu_s, G4sigma_s), nrow = 1) %*%
               matrix(c(D_Edox2_Y3_s, D_Vdox2_Y3_s), nrow = 2, byrow = TRUE)

# AV of the estimator of the interventional probability
# according to Equation 17
AV_gamma4_s <- D_P_y3_dox2_s %*% AV_theta_s %*% t(D_P_y3_dox2_s)

# ASE of estimate of P(ylow < Y3 < yup | dox2) as in Table 2
# with ylow=-40 and yup=80
ASE_gamma4_s <- sqrt(AV_gamma4_s)

# approximate z-values as displayed in Table 2
# which are computed based on rounded values
z_gamma4_rounded_s <- round(round(P_y3_dox2_s, 4) /
                    round(sqrt(AV_gamma4_s), 4), 4)
```

S.7 Mathematica Code

The calculations were done on Windows 10 Pro (64-bit), platform: x86_64-w64-mingw32/x64 (64-bit), with Mathematica (Version Number: 11.3.0.0) (Wolfram Research Inc., 2018).

The following code is used to compute the analytic expressions stated in Section S.3.

```
(* --- SET UP THE LINEAR SEM FROM THE ILLUSTRATION SECTION --- *)

(* matrix of structural coefficients as in Equation S.4 (see also Equation 19 of the paper); since the symbol C is protected in
      Mathematica, we label the matrix as C6, where 6 is the number of observed variables*)
C6={{0,0,0,0,0,0},{0,0,0,0,0,0},{cxx,cxy,0,0,0,0},{cyx,cyy,0,0,0,0},{0,0,cxx,cxy,0,0},{0,0,cyx,cyy,0,0}};

(* covariance matrix of the error terms stated in Equation S.5 (see also: Equation 20 of the paper) *)
PSI={{psix1x1,psix1y1,psix1x2,0,0,0},{psix1y1,psiy1y1,0,psiy1y2,0,0},{psix1x2,0,psixx,psixy,psix2x3,0},{0,psiy1y2,psixy,psiyy,0,
      psiy2y3},{0,0,psix2x3,0,psixx,psixy},{0,0,0,psiy2y3,psixy,psiyy}};

(* mean vector of the error terms which is zero *)
Eepsilon={{0},{0},{0},{0},{0},{0}};
```

```
(* --- COMPUTE THE MODEL IMPLIED DISTRIBUTION OF OBSERVED VARIABLES --- *)

(* model implied mean vector of the observed variables (see Equation 2 of the paper) *)
EV=Inverse[IdentityMatrix[6]-C6].Eepsilon;

(* model implied covariance matrix of the observed variables as stated in Equation S.6 (see also: Equation 2 of the paper) *)
SigmaV=Inverse[IdentityMatrix[6]-C6].PSI.Transpose[Inverse[IdentityMatrix[6]-C6]];

(* collect terms in the model implied covariance matrix of the observed variables *)
SigmaV=Collect[SigmaV,{psix1x1,psiy1y1,psix1y1,psixx,psiyy,psixy,psix1x2,psix2x3,psiy1y2,psiy2y3}];

(* model implied variance of X2 as stated in Equation S.7a *)
VX2=SigmaV[[3,3]];

(* model implied variance of Y3 as stated in Equation S.7b *)
VY3=SigmaV[[6,6]];

(* model implied covariance of X2 and Y3 as stated in Equation S.7c *)
COVX2Y3=SigmaV[[3,6]];

(* conditional mean of Y3 given X2=x2 as stated in Equation S.9 *)
EY3condX2=COVX2Y3/VX2*x2;

(* conditional variance of Y3 given X2=x2 as stated in Equation S.10 *)
VY3condX2= VY3-(COVX2Y3^2/VX2);

(* --- CALCULATE THE INTERVENTIONAL MOMENTS FOR do(X2=x2) --- *)

(* selection matrix for the interventional variable as stated in Equation S.14 (see also Equation 21 of the paper) *)
e3={{0},{0},{1},{0},{0},{0}};

(* zero-one matrix that sets the interventional rows to zero as stated in Equation S.14 (see also Equation 21 of the paper) *)
IN={{1,0,0,0,0,0},{0,1,0,0,0,0},{0,0,0,0,0,0},{0,0,0,1,0,0},{0,0,0,0,1,0},{0,0,0,0,0,1}};

(* transformation matrix T1 as stated in Equation 4 of the paper *)
T1=Inverse[IdentityMatrix[6]-IN.C6].IN;

(* vector a1 as stated in Equation 4 of the paper *)
a1=Inverse[IdentityMatrix[6]-IN.C6].e3;

(* inverventional mean vector as stated in Equation Equation S.13a (see also Equation 6a of the paper) *)
Edox2=a1*x2;

(* inverventional covariance matrix as stated in Equation Equation S.13b (see also Equation 6b of the paper) *)

Vdox2=T1.PSI.Transpose[T1];

(* collect terms in the model implied covariance matrix of the observed variables *)

Vdox2=Collect[Vdox2,{psix1x1,psiy1y1,psix1y1,psixx,psiyy,psixy,psix1x2,psix2x3,psiy1y2,psiy2y3}];

(* interventional mean of Y3 given do(X2=x2) as stated in Equation S.15 (see also Equation 22a of the paper) *)
EY3dox2=Edox2[[6]];

(* interventional variance of Y3 given do(X2=x2) as stated in Equation S.16 (see also Equation 22b of the paper) *)
VY3dox2=Vdox2[[6,6]];
```

The following code is used to evaluate the rank of the Jacobian matrix stated in Equation (S.29) in Section S.5.

```
(* matrix of structural coefficients
 according to Equation 3.2.1 in Bekker et al. (1994);
 this matrix corresponds to the matrix I-C from the paper;
 see also Table S.3 of this electronic supplementary material *)

IminusC6={{1,0,0,0,0,0},{0,1,0,0,0,0},{-cxx,-cxy,1,0,0,0},
{-cyx,-cyy,0,1,0,0},{0,0,-cxx,-cxy,1,0},{0,0,-cyx,-cyy,0,1}};

(* Covariance matrix of error terms according to Bekker et al. (1994);
 this matrix corresponds to the matrix Psi from the paper;
 see also Table S.3 of this electronic supplementary material *)

Psi={{psix1x1,psix1y1,psix1x2,0,0,0},{psix1y1,psiy1y1,0,psiy1y2,0,0},
{psix1x2,0,psixx,psixy,psix2x3,0},{0,psiy1y2,psixy,psiyy,0,psiy2y3},
{0,0,psix2x3,0,psixx,psixy},{0,0,0,psiy2y3,psixy,psiyy}};

(* restriction matrix R_Psi;
 see Equation (S.31) of this electronic supplementary material *)

RPsi={{0,0,0,1,0,0,0,0,0,0,0,0,0,0,0,0,0,0,0,0,0,0,0,0,0,0,0,0,0,0,0,0,0,0,0,0},
{0,0,0,0,1,0,0,0,0,0,0,0,0,0,0,0,0,0,0,0,0,0,0,0,0,0,0,0,0,0,0,0,0,0,0,0},
{0,0,0,0,0,1,0,0,0,0,0,0,0,0,0,0,0,0,0,0,0,0,0,0,0,0,0,0,0,0,0,0,0,0,0,0},
{0,0,0,0,0,0,0,0,1,0,0,0,0,0,0,0,0,0,0,0,0,0,0,0,0,0,0,0,0,0,0,0,0,0,0,0},
{0,0,0,0,0,0,0,0,0,0,1,0,0,0,0,0,0,0,0,0,0,0,0,0,0,0,0,0,0,0,0,0,0,0,0,0},
{0,0,0,0,0,0,0,0,0,0,0,1,0,0,0,0,0,0,0,0,0,0,0,0,0,0,0,0,0,0,0,0,0,0,0,0},
{0,0,0,0,0,0,0,0,0,0,0,0,0,0,0,0,0,1,0,0,0,0,0,0,0,0,0,0,0,0,0,0,0,0,0,0},
{0,0,0,0,0,0,0,0,0,0,0,0,0,0,0,0,0,0,0,0,0,0,1,0,0,0,0,0,0,0,0,0,0,0,0,0},
{0,0,0,0,0,0,0,0,0,0,0,0,0,0,1,0,0,0,0,0,0,0,0,0,0,0,0,0,-1,0,0,0,0,0,0,0},
{0,0,0,0,0,0,0,0,0,0,0,0,0,0,0,0,0,0,0,0,0,1,0,0,0,0,0,0,0,0,0,0,0,0,0,-1},
{0,0,0,0,0,0,0,0,0,0,0,0,0,0,0,1,0,0,0,0,0,0,0,0,0,0,0,0,0,-1,0,0,0,0,0,0}};

Dimensions[RPsi]

(* restriction matrix R_C;
 see Equation (S.32) of this electronic supplementary material *)

RC={{1,0,0,0,0,0,0,0,0,0,0,0,0,0,0,0,0,0,0,0,0,0,0,0,0,0,0,0,0,0,0,0,0,0,0,0},
{0,1,0,0,0,0,0,0,0,0,0,0,0,0,0,0,0,0,0,0,0,0,0,0,0,0,0,0,0,0,0,0,0,0,0,0},
{0,0,1,0,0,0,0,0,0,0,0,0,0,0,0,0,0,0,0,0,0,0,0,0,0,0,0,0,0,0,0,0,0,0,0,0},
{0,0,0,1,0,0,0,0,0,0,0,0,0,0,0,0,0,0,0,0,0,0,0,0,0,0,0,0,0,0,0,0,0,0,0,0},
{0,0,0,0,1,0,0,0,0,0,0,0,0,0,0,0,0,0,0,0,0,0,0,0,0,0,0,0,0,0,0,0,0,0,0,0},
{0,0,0,0,0,1,0,0,0,0,0,0,0,0,0,0,0,0,0,0,0,0,0,0,0,0,0,0,0,0,0,0,0,0,0,0},
{0,0,0,0,0,0,1,0,0,0,0,0,0,0,0,0,0,0,0,0,0,0,0,0,0,0,0,0,0,0,0,0,0,0,0,0},
{0,0,0,0,0,0,0,1,0,0,0,0,0,0,0,0,0,0,0,0,0,0,0,0,0,0,0,0,0,0,0,0,0,0,0,0},
{0,0,0,0,0,0,0,0,1,0,0,0,0,0,0,0,0,0,0,0,0,0,0,0,0,0,0,0,0,0,0,0,0,0,0,0},
{0,0,0,0,0,0,0,0,0,1,0,0,0,0,0,0,0,0,0,0,0,0,0,0,0,0,0,0,0,0,0,0,0,0,0,0},
{0,0,0,0,0,0,0,0,0,0,1,0,0,0,0,0,0,0,0,0,0,0,0,0,0,0,0,0,0,0,0,0,0,0,0,0},
{0,0,0,0,0,0,0,0,0,0,0,1,0,0,0,0,0,0,0,0,0,0,0,0,0,0,0,0,0,0,0,0,0,0,0,0},
{0,0,0,0,0,0,0,0,0,0,0,0,0,0,1,0,0,0,0,0,0,0,0,0,0,0,0,0,0,0,0,0,0,0,0,0},
{0,0,0,0,0,0,0,0,0,0,0,0,0,0,0,1,0,0,0,0,0,0,0,0,0,0,0,0,0,0,0,0,0,0,0,0},
{0,0,0,0,0,0,0,0,0,0,0,0,0,0,0,0,1,0,0,0,0,0,0,0,0,0,0,0,0,0,0,0,0,0,0,0},
{0,0,0,0,0,0,0,0,0,0,0,0,0,0,0,0,0,1,0,0,0,0,0,0,0,0,0,0,0,0,0,0,0,0,0,0},
{0,0,0,0,0,0,0,0,0,0,0,0,0,0,0,0,0,0,0,0,1,0,0,0,0,0,0,0,0,0,0,0,0,0,0,0},
{0,0,0,0,0,0,0,0,0,0,0,0,0,0,0,0,0,0,0,0,0,1,0,0,0,0,0,0,0,0,0,0,0,0,0,0},
{0,0,0,0,0,0,0,0,0,0,0,0,0,0,0,0,0,0,0,0,0,0,1,0,0,0,0,0,0,0,0,0,0,0,0,0},
{0,0,0,0,0,0,0,0,0,0,0,0,0,0,0,0,0,0,0,0,0,0,0,1,0,0,0,0,0,0,0,0,0,0,0,0},
{0,0,0,0,0,0,0,0,0,0,0,0,0,0,0,0,0,0,0,0,0,0,0,0,1,0,0,0,0,0,0,0,0,0,0,0},
{0,0,0,0,0,0,0,0,0,0,0,0,0,0,0,0,0,0,0,0,0,0,0,0,0,1,0,0,0,0,0,0,0,0,0,0},
```

```
{0,0,0,0,0,0,0,0,0,0,0,0,0,0,0,0,0,0,0,0,0,0,0,0,0,0,0,0,1,0,0,0,0,0,0,0},
{0,0,0,0,0,0,0,0,0,0,0,0,0,0,0,0,0,0,0,0,0,0,0,0,0,0,0,0,0,1,0,0,0,0,0,0},
{0,0,0,0,0,0,0,0,0,0,0,0,0,0,0,0,0,0,0,0,0,0,0,0,0,0,0,0,0,0,1,0,0,0,0,0},
{0,0,0,0,0,0,0,0,0,0,0,0,0,0,0,0,0,0,0,0,0,0,0,0,0,0,0,0,0,0,0,1,0,0,0,0},
{0,0,0,0,0,0,0,0,0,0,0,0,0,0,0,0,0,0,0,0,0,0,0,0,0,0,0,0,0,0,0,0,0,0,1,0},
{0,0,0,0,0,0,0,0,0,0,0,0,0,0,0,0,0,0,0,0,0,0,0,0,0,0,0,0,0,0,0,0,0,0,0,1},
{0,0,0,0,0,0,0,0,0,0,0,0,1,0,0,0,0,0,0,0,0,0,0,0,0,0,-1,0,0,0,0,0,0,0,0,0},
{0,0,0,0,0,0,0,0,0,0,0,0,0,1,0,0,0,0,0,0,0,0,0,0,0,0,0,-1,0,0,0,0,0,0,0,0},
{0,0,0,0,0,0,0,0,0,0,0,0,0,0,0,0,0,0,1,0,0,0,0,0,0,0,0,0,0,0,0,0,-1,0,0,0},
{0,0,0,0,0,0,0,0,0,0,0,0,0,0,0,0,0,0,0,1,0,0,0,0,0,0,0,0,0,0,0,0,0,-1,0,0}};

Dimensions[RC]

(* commutation matrix for m=n=6, see Equation 1.5.1 in Bekker et al. (1994) *)

K6={{1,0,0,0,0,0,0,0,0,0,0,0,0,0,0,0,0,0,0,0,0,0,0,0,0,0,0,0,0,0,0,0,0,0,0,0},
{0,0,0,0,0,0,1,0,0,0,0,0,0,0,0,0,0,0,0,0,0,0,0,0,0,0,0,0,0,0,0,0,0,0,0,0},
{0,0,0,0,0,0,0,0,0,0,0,0,1,0,0,0,0,0,0,0,0,0,0,0,0,0,0,0,0,0,0,0,0,0,0,0},
{0,0,0,0,0,0,0,0,0,0,0,0,0,0,0,0,0,0,1,0,0,0,0,0,0,0,0,0,0,0,0,0,0,0,0,0},
{0,0,0,0,0,0,0,0,0,0,0,0,0,0,0,0,0,0,0,0,0,0,0,0,1,0,0,0,0,0,0,0,0,0,0,0},
{0,0,0,0,0,0,0,0,0,0,0,0,0,0,0,0,0,0,0,0,0,0,0,0,0,0,0,0,0,0,1,0,0,0,0,0},
{0,1,0,0,0,0,0,0,0,0,0,0,0,0,0,0,0,0,0,0,0,0,0,0,0,0,0,0,0,0,0,0,0,0,0,0},
{0,0,0,0,0,0,0,1,0,0,0,0,0,0,0,0,0,0,0,0,0,0,0,0,0,0,0,0,0,0,0,0,0,0,0,0},
{0,0,0,0,0,0,0,0,0,0,0,0,0,1,0,0,0,0,0,0,0,0,0,0,0,0,0,0,0,0,0,0,0,0,0,0},
{0,0,0,0,0,0,0,0,0,0,0,0,0,0,0,0,0,0,0,1,0,0,0,0,0,0,0,0,0,0,0,0,0,0,0,0},
{0,0,0,0,0,0,0,0,0,0,0,0,0,0,0,0,0,0,0,0,0,0,0,0,0,1,0,0,0,0,0,0,0,0,0,0},
{0,0,0,0,0,0,0,0,0,0,0,0,0,0,0,0,0,0,0,0,0,0,0,0,0,0,0,0,0,0,0,1,0,0,0,0},
{0,0,1,0,0,0,0,0,0,0,0,0,0,0,0,0,0,0,0,0,0,0,0,0,0,0,0,0,0,0,0,0,0,0,0,0},
{0,0,0,0,0,0,0,0,1,0,0,0,0,0,0,0,0,0,0,0,0,0,0,0,0,0,0,0,0,0,0,0,0,0,0,0},
{0,0,0,0,0,0,0,0,0,0,0,0,0,0,1,0,0,0,0,0,0,0,0,0,0,0,0,0,0,0,0,0,0,0,0,0},
{0,0,0,0,0,0,0,0,0,0,0,0,0,0,0,0,0,0,0,0,1,0,0,0,0,0,0,0,0,0,0,0,0,0,0,0},
{0,0,0,0,0,0,0,0,0,0,0,0,0,0,0,0,0,0,0,0,0,0,0,0,0,0,1,0,0,0,0,0,0,0,0,0},
{0,0,0,0,0,0,0,0,0,0,0,0,0,0,0,0,0,0,0,0,0,0,0,0,0,0,0,0,0,0,0,0,1,0,0,0},
{0,0,0,1,0,0,0,0,0,0,0,0,0,0,0,0,0,0,0,0,0,0,0,0,0,0,0,0,0,0,0,0,0,0,0,0},
{0,0,0,0,0,0,0,0,0,1,0,0,0,0,0,0,0,0,0,0,0,0,0,0,0,0,0,0,0,0,0,0,0,0,0,0},
{0,0,0,0,0,0,0,0,0,0,0,0,0,0,0,1,0,0,0,0,0,0,0,0,0,0,0,0,0,0,0,0,0,0,0,0},
{0,0,0,0,0,0,0,0,0,0,0,0,0,0,0,0,0,0,0,0,0,1,0,0,0,0,0,0,0,0,0,0,0,0,0,0},
{0,0,0,0,0,0,0,0,0,0,0,0,0,0,0,0,0,0,0,0,0,0,0,0,0,0,0,1,0,0,0,0,0,0,0,0},
{0,0,0,0,0,0,0,0,0,0,0,0,0,0,0,0,0,0,0,0,0,0,0,0,0,0,0,0,0,0,0,0,0,1,0,0},
{0,0,0,0,1,0,0,0,0,0,0,0,0,0,0,0,0,0,0,0,0,0,0,0,0,0,0,0,0,0,0,0,0,0,0,0},
{0,0,0,0,0,0,0,0,0,0,1,0,0,0,0,0,0,0,0,0,0,0,0,0,0,0,0,0,0,0,0,0,0,0,0,0},
{0,0,0,0,0,0,0,0,0,0,0,0,0,0,0,0,1,0,0,0,0,0,0,0,0,0,0,0,0,0,0,0,0,0,0,0},
{0,0,0,0,0,0,0,0,0,0,0,0,0,0,0,0,0,0,0,0,0,0,1,0,0,0,0,0,0,0,0,0,0,0,0,0},
{0,0,0,0,0,0,0,0,0,0,0,0,0,0,0,0,0,0,0,0,0,0,0,0,0,0,0,0,1,0,0,0,0,0,0,0},
{0,0,0,0,0,0,0,0,0,0,0,0,0,0,0,0,0,0,0,0,0,0,0,0,0,0,0,0,0,0,0,0,0,0,1,0},
{0,0,0,0,0,1,0,0,0,0,0,0,0,0,0,0,0,0,0,0,0,0,0,0,0,0,0,0,0,0,0,0,0,0,0,0},
{0,0,0,0,0,0,0,0,0,0,0,1,0,0,0,0,0,0,0,0,0,0,0,0,0,0,0,0,0,0,0,0,0,0,0,0},
{0,0,0,0,0,0,0,0,0,0,0,0,0,0,0,0,0,1,0,0,0,0,0,0,0,0,0,0,0,0,0,0,0,0,0,0},
{0,0,0,0,0,0,0,0,0,0,0,0,0,0,0,0,0,0,0,0,0,0,0,1,0,0,0,0,0,0,0,0,0,0,0,0},
{0,0,0,0,0,0,0,0,0,0,0,0,0,0,0,0,0,0,0,0,0,0,0,0,0,0,0,0,0,1,0,0,0,0,0,0},
{0,0,0,0,0,0,0,0,0,0,0,0,0,0,0,0,0,0,0,0,0,0,0,0,0,0,0,0,0,0,0,0,0,0,0,1}};

(* M-matrix;
 see Section 1.5 (below Equation 1.5.1) in Bekker et al. (1994) for p=6 *)

M6=1/2*(IdentityMatrix[36]+K6);

(* Kronecker product in the first entry of the matrix on the right-hand side
 of Equation 3.3.6 in Bekker et al. (1994);
 see also Equation (S.29) of this electronic supplementary material *)
```

```
FPsi=KroneckerProduct[IdentityMatrix[6],Psi];

(* First entry of the matrix on the right-hand side
 of Equation 3.3.6 in Bekker et al. (1994);
 see also Equation (S.29) of this electronic supplementary material *)

J1=2*RPsi.M6.FPsi;

(* Kronecker product in the second entry of the matrix on the right-hand side
 of Equation 3.3.6 in Bekker et al. (1994);
 see also Equation (S.29) of this electronic supplementary material *)

FC=KroneckerProduct[IdentityMatrix[6],Transpose[IminusC6]];

(* Second entry of the matrix on the right-hand side
 of Equation 3.3.6 in Bekker et al. (1994);
 see also Equation (S.29) of this electronic supplementary material *)

J2=RC.FC;

(* Join first and second entry of the matrix on the right-hand side
 of Equation 3.3.6 in Bekker et al. (1994) to obtain the Jacobian matrix;
 see also Equation (S.29) of this electronic supplementary material *)

J=Join[J1,J2];

(* Rank evaluation of the Jacobian matrix *)

MatrixRank[J]
NullSpace[J]
Dimensions[J]
```

References

Bekker, P. A., Merckens, A., & Wansbeek, T. J. (1994). *Identification, equivalent models, and computer algebra.* San Diego, CA: Academic Press.

Bollen, K. A. (1987). Total, direct, and indirect effects in structural equation models. *Sociological Methodology*, *17*, 37–69. https://doi.org/10.2307/271028

Bollen, K. A. (1989). *Structural equations with latent variables.* New York, NY: John Wiley & Sons. https://doi.org/10.1002/9781118619179

Hamilton, J. (1994). *Time series analysis.* Princeton, NJ: Princeton University Press.

Ito, K., Wada, T., Makimura, H., Matsuoka, A., Maruyama, H., & Saruta, T. (1998). Vector autoregressive modeling analysis of frequently sampled oral glucose tolerance test results. *The Keio Journal of Medicine*, *47*(1), 28–36. https://doi.org/10.2302/kjm.47.28

Lütkepohl, H. (2005). *New introduction to multiple time series analysis.* Berlin, Germany: Springer-Verlag. https://doi.org/10.1007/978-3-540-27752-1

Pfaff, B. (2008). VAR, SVAR and SVEC models: Implementation within R package vars. *Journal of Statistical Software*, *27*(4). https://doi.org/10.18637/jss.v027.i04

R Core Team. (2019). R: A language and environment for statistical computing [Computer software manual]. Vienna, Austria. Retrieved from https://www.R-project.org/

Rao, C. (1973). *Linear statistical inference and its applications* (2nd ed.). New York, NY: Wiley.

Rosseel, Y. (2012). lavaan: An R package for structural equation modeling. *Journal of Statistical Software*, *48*(2), 1–36. https://doi.org/10.18637/jss.v048.i02

Wolfram Research Inc. (2018). Mathematica, Version 11.3 [Computer software manual]. Champaign, IL. Retrieved from https://www.wolfram.com/mathematica
